# Synthesis of Citrifurans A and D through Assembly of the Natural Products Citrinin and Gregatin A

**DOI:** 10.1021/acs.orglett.5c02665

**Published:** 2025-07-22

**Authors:** Yuto Goto, Ayaka Machiya, Taichi Okumura, Yusuke Oka, Junya Ito, Osamu Ishibashi, Yoshihito Shiono, Yasuhiro Meguro, Kiyotaka Nakagawa, Shigefumi Kuwahara, Masaru Enomoto

**Affiliations:** † Graduate School of Agricultural Science, 13101 Tohoku University, 468-1 Aramaki Aza-Aoba, Aoba-ku, Sendai 980-8572, Japan; ‡ Faculty of Agriculture, Tohoku University, 468-1 Aramaki Aza-Aoba, Aoba-ku, Sendai 980-8572, Japan; § Faculty of Agriculture, Yamagata University, Tsuruoka, Yamagata 997-8555, Japan

## Abstract

The synthesis of citrifuran A, a structurally unique natural heterodimer, was accomplished in a bioinspired manner by a thermal [4 + 2] cycloaddition between citrinin and gregatin A, the former of which was synthesized via a Diels–Alder-initiated aromatization reaction and the latter via a Cu-mediated 5-*endo* cyclization–bromination as the key steps, respectively. On the other hand, an acid/base-promoted cycloaddition of citrinin with gregatin A followed by hydrolysis of the resulting intermediate furnished citrifuran D.

## Graphical abstract

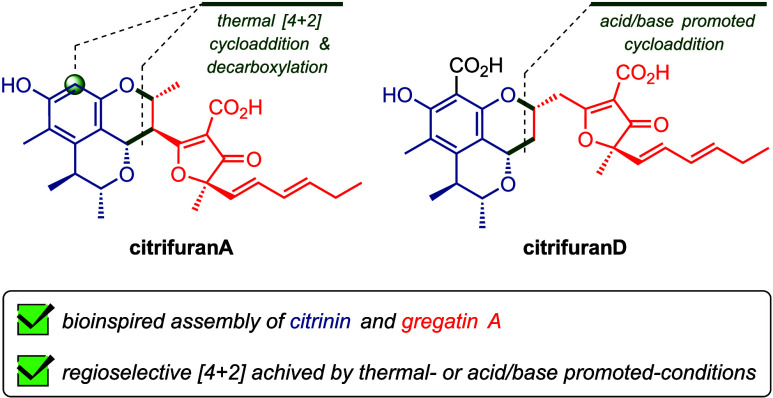

In the course of searching for novel bioactive substances from fungal resources, Kong et al. isolated novel heterodimeric polyketides, citrifurans A (**1**) and D (**2**) (Figure 1),^1,2^ along with their biosynthetically related compounds, from the extracts of a solid culture of sp. discovered in the centipede intestine. They determined the structures of these secondary metabolites, including the absolute configuration, based on NMR experiments, X-ray crystallography, and electronic circular dichroism (ECD) spectral analysis and reported moderate inhibitory activities of **1** against lipopolysaccharide (LPS)-induced NO production in RAW 264.7 macrophages. Citrifurans A (**1**) and D (**2**) feature unique tetracyclic frameworks composed of azaphilone (blue) and furanone (red) moieties. Therefore, we presumed that **1** might form through the formal [4 + 2] cycloaddition of decarboxycitrinin (**3**) with the keto form of nivefuranone A (**4**) in nature, whereas **2** would be generated from citrinin (**5**) and the enol form of **4**. Citrinin (**5**)^3,4^ is a well-known mycotoxin produced by several fungal strains in the genera, such as , , and , and various heterodimeric natural products with pharmacologically important biological profiles, such as antitumor, antimicrobial, and antioxidative activities, have been reported for its related compounds.^5^ Despite their intriguing biological properties and considerable structural diversity, only a few citrinin-related dimers have been successfully synthesized.^6^ Herein, we describe the synthesis of citrifurans A (**1**) and D (**2**) by combining citrinin (**5**) and gregatin A (**6**),^7,8^ which is the methyl ester of **4**.

**Figure 1:**
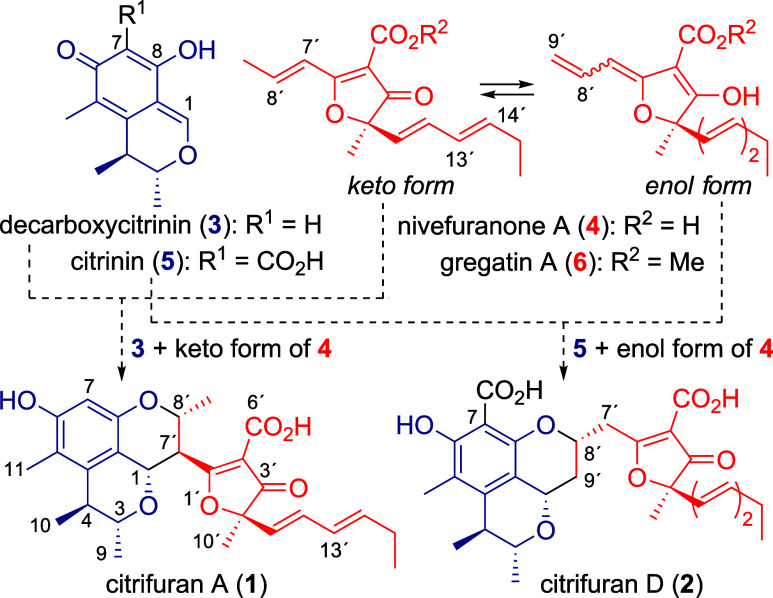
Structures of citrifurans A (**1**) and D (**2**), decarboxycitrinin (**3**), nivefuranone A (**4**), citrinin (**5**), and gregatin A (**6**) and their plausible biosynthetic pathways.

Our retrosynthetic analysis of **1** and **2** is shown in Scheme 1. Based on several analogous precedents,^6c,9^ we expected that cycloaddition yielding **1** could take place stereoselectively by simply mixing **3** and **4** under appropriate conditions. This is because **1** should be formed via transition state **TS1**, which was considered to have the least repulsion between each substituent at the C2′, C3, and C4 positions. On the other hand, **2** was assumed to be available through a sequential Michael addition/cyclization between **5** and the enol form of **4**, although the stereocontrol might be difficult, unlike in the case of **1**. Furanone **4** would be preparable by a several-step sequence including the 5-*endo*-*dig* cyclization of **7**. Ynone **7** would be accessible by the nucleophilic acyl substitution of Weinreb’s amide **8** with alkynylmetal reagent **9**, the former of which would readily be available from known dioxolanone **10**.^10^ For the synthesis of the azaphilone segments, we planned to obtain **3** and **5** from stoloniferol B (**11**)^11^ by reducing lactone and subsequent dehydration of the resulting lactol. To construct the benzene ring of **11** by the Diels–Alder-initiated aromatization reaction,^12^ **11** was retrosynthetically traced back to known diene **13** ^13^ and dienophile **14**, the latter of which should readily be preparable from known chiral δ-lactone **15**.^14^

**Scheme 1:**
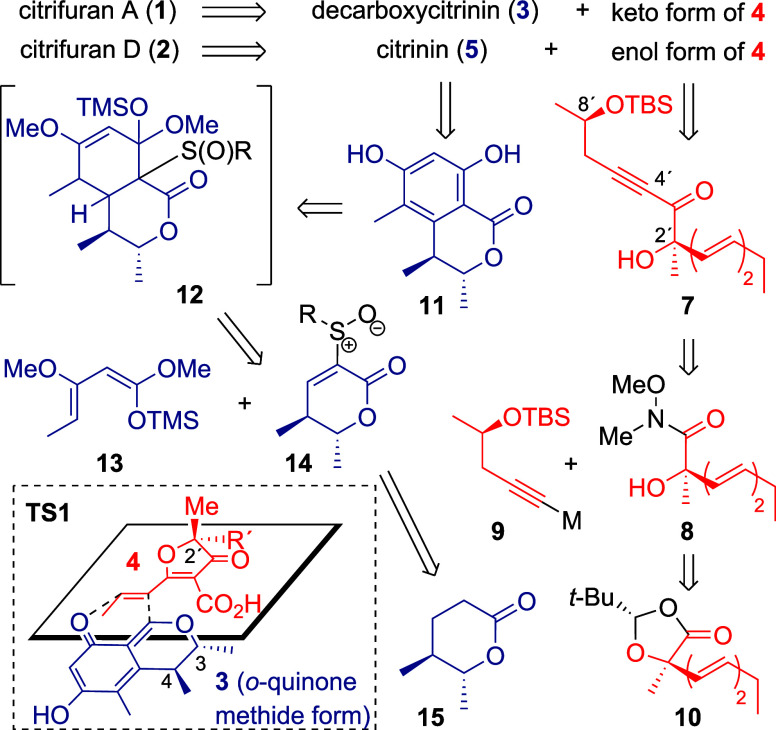
Retrosynthetic Analysis of **1** and **2**

Our synthesis of the furanone segment commenced with conversion of **10** to **8** via two conventional steps (Scheme 2A).^10,15^ Weinreb’s amide **8** then reacted with the alkynyl lithium species, which was generated *in situ* by treating **16** ^16^ with *n*-BuLi at −78 °C, to afford **7**. Next, we investigated the key 5-*endo*-*dig* cyclization to construct the 3-furanone core. The desired transformation could be effected in 78% yield by treating **7** with NIS in the presence of a catalytic amount of PPh_3_ in 1,2-DCE.^17^ However, this reaction isomerized the C13′–C14′ double bond, providing the iodide congener of **17** as an inseparable mixture of *E*/*Z* isomers (88:12). This problem was overcome by subjecting **7** to Cu-mediated cyclization–bromination^18^ to furnish **17** as a single geometrical isomer. Notably, the *n*-hexane/DMF biphasic system effectively prevented byproduct formation, including the dimer of **17**. Installing a methoxycarbonyl group at the C4′ position was realized by adding *n*-BuLi to a suspension of **17** in *n*-hexane at −85 °C, followed by trapping the resulting dissolved anion with NCCO_2_Me to furnish **18**. Although Brückner’s pioneering work^10^ demonstrated the route to **6** from **18**, there remained room for improvement in the yield. Fortunately, exposure of **18** to TMSCl/MeCN^19^ afforded **6** and gregatin D (**19**)^7,10^ in isolated yields of 68 and 30%, respectively; the latter could be converted to the former in 91% yield under the same reaction conditions. The final step was the conversion of **6** into **4**, but all attempts were unsuccessful. Therefore, the furanone segment in the assembly reaction was changed from **4** to **6**.

**Scheme 2:**
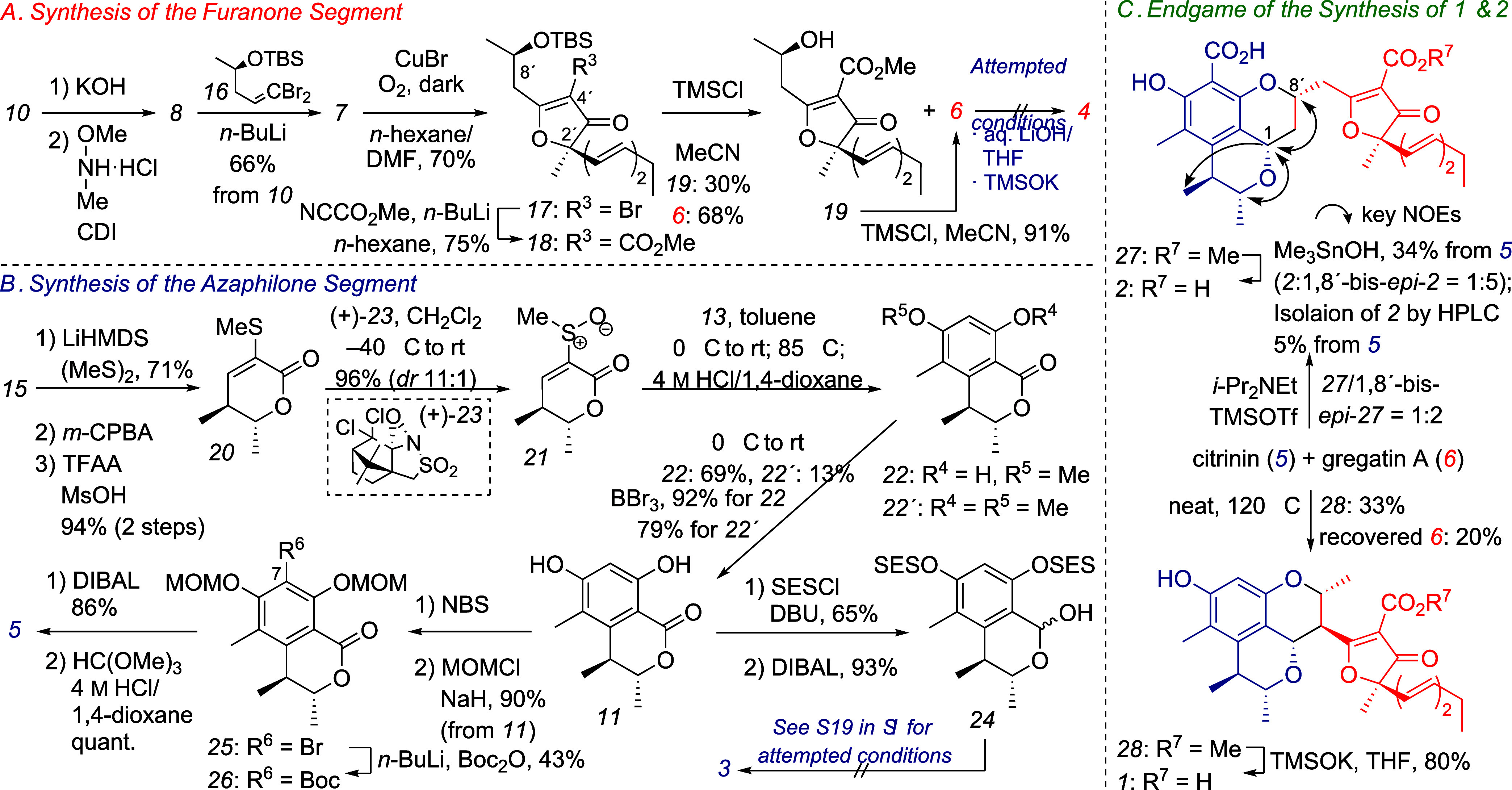
Synthetic Scheme of **1** and **2**

We next set about the preparation of azaphilone segments **3** and **5** (Scheme 2B). First, **15** was transformed into **20** by a three-step sequence, including installation of the double bond via a Pummerer rearrangement-type oxidation^20^ of intermediary sulfoxide. Diastereoselective oxidation of sulfide **20** was essential since one of the two epimers of **21** was found to preferentially undergo the Diels–Alder reaction with **13** (see page S18 in the Supporting Information). The Diels–Alder-initiated aromatization reaction was eventually achieved in 82% total yield of **22** and **22′** using a 11:1 epimeric mixture of **21** prepared by treating **20** with (+)-**23** ^21^ at −40 °C. Removal of the methyl groups of **22** and **22′** with BBr_3_ proceeded smoothly to afford **11**. Next, we attempted to convert **11** into **3** via 2-(trimethylsilyl)­ethanesulfonyl (SES)-protected lactol **24**. However, subjecting **24** to various deprotection conditions resulted in the formation of a complex mixture (see page S19 in the Supporting Information). Faced with the difficulties in preparing **3**, we decided to synthesize **1** by combining **6** with **5** instead of **3**, followed by the decarboxylation of the resulting product. Thus, a *tert*-butoxy carbonyl (Boc) group was installed at the C7 position via the halogen–lithium exchange of bromide **25** derived from **11**.^22^ Finally, DIBAL reduction of **26** followed by treatment of the resulting lactol with HC­(OMe)_3_ and 4 M HCl/dioxane^23^ completed the synthesis of **5**.

Next, the assembly and subsequent elaboration of **5** and **6** into **1** and **2** was explored (Scheme 2C). A solution of **5** and **6** in THF was treated with i-Pr_2_NEt and TMSOTf in attempts to synthesize **2** via sequential Michael addition/cyclization. Expectedly, the desired reaction proceeded, but the product obtained in ca. 67% yield was an inseparable diastereomeric mixture including **27** and 1,8′-bis-*epi*-**27** as the major components in a ratio of 1:2. Therefore, the mixture was used without further purification. The final stage of the synthesis of **2** was challenging. The conversion of the diastereomeric mixtures of **27** into **2** was attempted under several conditions, such as TMSOK/THF^24^ and aqueous LiOH/THF; however, none gave **2**, and the substrate decomposed, probably due to the retro-Michael reaction. This challenging task was achieved by subjecting the mixture to hydrolysis conditions using Me_3_SnOH,^25^ furnishing a 1:5 diastereomeric mixture of **2** and 1,8′-bis-*epi*-**2** in 34% over two steps, from which **2** was isolated by HPLC purification (5% from **5**). The desired diastereomer **27** appeared to decompose more quickly than 1,8′-bis-*epi*-**27** under the hydrolysis conditions. Next, we addressed the synthesis of compound **1**. According to the analogous precedent,^6c^ we first refluxed a solution of **5** and **6** in toluene. Delightedly, not only the cycloaddition of both segments but also the decarboxylation at the C7 position proceeded in one pot, affording **28**, albeit in a trace yield, along with 76% of recovered **6**. Optimized assembly of both segments was accomplished by heating a mixture of **5** and **6** under solvent-free (neat) conditions at 120 °C for 19 h, which provided **28** in 33% yield as the major diastereomer along with a trace amount of a mixture of other diastereomers. Finally, the removal of the methyl group at the ester functionality of **28** with TMSOK^24^ completed the synthesis of **1**.

In summary, the synthesis of citrifurans A (**1**) and D (**2**) was accomplished in a bioinspired manner by the thermal [4 + 2] cycloaddition between citrinin (**5**) and gregatin A (**6**) for **1** and the acid/base-promoted cycloaddition of both segments for **2**. Synthetic studies of some related heterodimeric natural products are also in progress and will be reported later.

## Supplementary Material



## Data Availability

The data underlying this study are available in the published article and its Supporting Information.

## Notes

Experimental procedures, characterization data, and NMR spectra (PDF)
