# Supplementary material for: Synthesis of Citrifurans A and D through Assembly of the Natural Products Citrinin and Gregatin A
Source: Org Lett. 2025 Jul 22;27(30):8349–52. doi: 10.1021/acs.orglett.5c02665 (PMC12322954; doi:10.1021/acs.orglett.5c02665)
Supplement: Supplementary file 1 [file ol5c02665_si_001.pdf]

Supporting Information

Synthesis of Citrifurans A and D through Assembly of the Natural Products Citrinin and Gregatin A

Yuto Goto,[†] Ayaka Machiya,[†] Taichi Okumura,[‡] Yusuke Oka,[†] Junya Ito,[†] Osamu Ishibashi,[†] Yoshihito Shiono,[¶] Yasuhiro Meguro,[†] Kiyotaka Nakagawa,[†] Shigefumi Kuwahara,[†] Masaru Enomoto[†*]

[†] Graduate School of Agricultural Science, Tohoku University, 468-1 Aramaki Aza-Aoba, Aoba-ku, Sendai 980-8572, Japan, [‡]Faculty of Agriculture, Tohoku University, 468-1 Aramaki Aza-Aoba, Aoba-ku, Sendai 980-8572, Japan, [¶]Faculty of Agriculture, Yamagata University, Tsuruoka, Yamagata 997-8555, Japan

Email : masaru.enomoto.a2@tohoku.ac.jp

Table of contents

1. General Information--S1
2. Experimental Procedures and Characterization data---S2
3. Determination of the Enantiomeric Excess of **10** ---S18
4. Influence of the diastereomeric ratio of sulfoxide **21** on the total yield of **22** and **22´** (Table S1) ---------------------S18
5. Attempted conversion of lactol **24** into decarboxycitrinin (**3**) or citrifuran A (**1**) ---S19
6. Comparison of the ^{1}H and ^{13}C NMR spectral data of synthetic **2** with those reported for natural citrifuran D ---------S20
7. Comparison of the ^{1}H and ^{13}C NMR spectral data of synthetic **1** with those reported for natural citrifuran A ---------S21
8. NMR Spectra-- S22

1. General Information

IR spectra were recorded by a Jasco FT/IR-4100 spectrometer using an ATR (ZnSe) attachment. NMR spectra were recorded by a Varian 600TT spectrometer (600 MHz for ^{1}H and 150 MHz for ^{13}C) unless otherwise stated. Chemical shifts (δ) were referenced to the residual solvent peaks as the internal standard (CD_2Cl_2: δ_H = 5.32, δ_C = 53.84; $CDCl_3$: δ_H = 7.26, δ_C = 77.02; benzene-d_6: δ_H = 7.15, δ_C = 128.00; DMSO-d_6: δ_H = 2.50, δ_C = 39.52). Structural assignments were made with additional information from gHSQC and gHMBC experiments. Optical rotation values were measured with a Jasco P-2200 polarimeter. Mass spectra were obtained with SCIEX X500R QTOF quadrupole time-of-flight mass spectrometer operated in the ESI mode. Melting points were determined with a Yanaco MP-J3 apparatus and are uncorrected. Merck silica gel 60 (63–200 μm) or Kanto Chemical silica gel 60N (spherical neutral, 63–210 μm or 40–50 μm) was used for column chromatography unless otherwise stated. Preparative thin-layer chromatography (TLC) was conducted using Merck silica gel 60 F254 plates (0.5 mm thick) or Merck silica gel 60 RP-18 F254s plates (0.25 mm thick) and analytical TLC was performed using Merck silica gel 60 F254 plates (0.25 mm thick). Preparative HPLC was carried out using a SHMADZU LC-6AD pump with a COSMOSIL Packed Column 5C18-MS-II (5 μm, 20×250 mm) and a SHMADZU SPD-20A detector. Solvents for reactions were distilled prior to use: THF, Et_2O and *n*-hexane from Na and benzophenone; CH_2Cl_2, DMF, and MeCN from CaH_2. Toluene was dried with 4Å molecular sieves (pellets) prior to use. All air- or moisture-sensitive reactions were conducted under a nitrogen atmosphere unless otherwise stated.

2. *Experimental Procedures and Characterization data*

(2*S*,5*S*)-2-(*tert*-butyl)-5-methyl-1,3-dioxolan-4-one (SI-1)

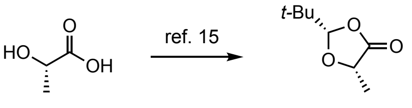

L-lactic acid SI-1

Compound **SI-1** was prepared from L-lactic acid by following the literature procedure cited in reference 15 in the text, affording **SI-1** as a mixture of diastereomers (*dr* 11:1, based on ^{1}H NMR measurement). After distillation to remove non-volatile by-products, evaporated compounds were crystallized from a solution of *n*-pentane/Et_2O (20:1, 2 mL per 1 g of **SI-1**) by gradually cooling to –80 °C to give **SI-1** (single diastereomer based on ^{1}H NMR measurement) as a colorless oil. The analytical data of **SI-1** were identical with those reported in the reference 15.

Specific Rotation: $[\alpha]_D^{24}$ +46.1 (*c* 0.995, $CHCl_3$).

^{1}H NMR (600 MHz, $CDCl_3$, ppm): δ = 5.14 (d, *J* = 1.2 Hz, 1H), 4.36 (dq, *J* = 1.2, 6.6 Hz, 1H), 1.48 (d, *J* = 6.6 Hz, 3H), 0.97 (s, 9H)

(2*S*,5*R*)-2-(*tert*-butyl)-5-((1*E*,3*E*)-hexa-1,3-dien-1-yl)-5-methyl-1,3-dioxolan-4-one (10)

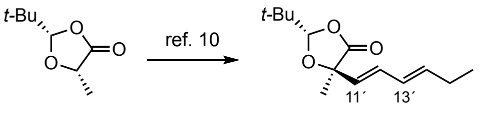

SI-1 10

SI-1 was transformed into dioxolanone **10** by following the literature procedure cited in reference 10 in the text, furnishing **10** as a mixture of geometrical isomers (*E,E*/11′*E*,13′*Z* = 6:1, based on ^{1}H NMR measurement). The undesired geometrical isomer of **10** could be separated by repeated SiO_2–$AgNO_3$ column chromatography [Kanto Chemical silica gel 60N (40–50 μm) impregnated with 10% (w/w) of silver nitrate, *n*-pentane/Et_2O= 60:1], delivering **10** as a single geometrical isomer (based on ^{1}H NMR measurement) as a colorless oil. The analytical data of **10** were identical with those reported in the reference 10.

Specific Rotation: $[\alpha]_D^{24}$ +112 (*c* 0.765, MeOH)

^{1}H NMR (600 MHz, $CDCl_3$, ppm): δ = 6.30 (dd, *J* = 15.6, 10.5 Hz, 1H), 6.02 (dd, *J* = 15.0, 10.5 Hz, 1H), 5.84 (dt, *J* = 15.0, 6.6 Hz, 1H), 5.52 (d, *J* = 15.6 Hz, 1H), 5.14 (s, 1H), 2.12 (m, 2H), 1.52 (s, 3H), 1.01 (t, *J* = 7.5 Hz, 3H), 0.98 (s, 9H)

^{13}C NMR (150 MHz, $CDCl_3$, ppm): δ = 173.9, 139.4, 131.8, 127.5, 125.9, 107.6, 80.0, 34.1, 25.7, 23.7, 23.4, 13.3

IR (ATR): ν_{max} 2965 (w), 1800 (vs), 1149 (w), 992 (w) cm^{-1}

HRMS (ESI) *m*/*z*: $[M+Na]^+$ Calcd for $C_{14}H_{22}NaO_3$ 261.1467; Found 261.1460

(*R*)-*tert*-butyl((5,5-dibromopent-4-en-2-yl)oxy)dimethylsilane (16)

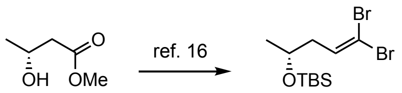

SI-2 16

Dibromoalkene **16** was prepared from **SI-2** by following the literature procedure cited in reference 16 in the text. **16** was obtained as a colorless oil. The analytical data of **16** were identical with those reported in the reference 16

Specific Rotation: $[\alpha]_D^{24}$ −2.7 (*c* 0.980, $CHCl_3$)

^{1}H NMR (600 MHz, $CDCl_3$, ppm): δ = 6.46 (t, *J* = 7.5 Hz, 1H), 3.92 (m, 1H), 2.26–2.18 (m, 2H), 1.15 (d, *J* = 6.0 Hz, 3H), 0.89 (s, 9H), 0.059 (s, 3H), 0.056 (s, 3H)

(2*R*,7*R*,8*E*,10*E*)-2-((*tert*-butyldimethylsilyl)oxy)-7-hydroxy-7-methyltrideca-8,10-dien-4-yn-6-one (7)

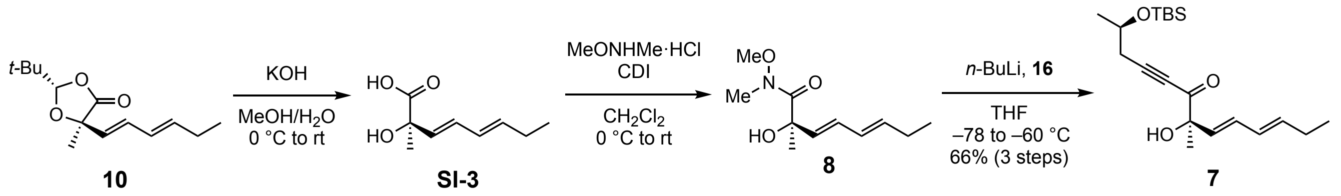

To a stirred suspension of **10** (3.95 g, 16.6 mmol) in H_2O (44 mL) was added dropwise a solution of KOH (3.71 g, 66.2 mmol, 4.0 equiv) in MeOH (132 mL) at 0 °C. After 15 min of stirring at the same temperature, the mixture was allowed to warm to room temperature and stirred for 4 h. The mixture was cooled to 0 °C and quenched with satd aq NH_4Cl (200 mL), and then MeOH was removed in vacuo. The resulting residue was diluted with EtOAc (300 mL), and then acidified using 12 M HCl until the pH reached to 2–3. The layers were separated and extracted with EtOAc. The combined organic layers were washed with brine, dried (Na_2SO_4), and concentrated in vacuo. The resulting crude carboxylic acid **SI-3** was used directly for the next reaction without further purification.

To a stirred solution of the crude carboxylic acid **SI-3** obtained above in CH_2Cl_2 (170 mL) was added portionwise carbonyldiimidazole (5.37 g, 33.1 mmol, 2.0 equiv) at 0 °C. The mixture was warmed to room temperature and stirred for 20 min, and then *N*,*O*-dimethylhydroxylamine hydrochloride (3.15 g, 32.3 mmol, 1.95 equiv) was added to this mixture. After 19 h of stirring at the same temperature, the mixture was quenched with satd aq $NaHCO_3$ (150 mL) and the layers were separated and extracted with EtOAc. The combined organic layers were washed with satd aq NH_4Cl and brine, dried (Na_2SO_4), and concentrated in vacuo. The residue was purified by SiO_2 column chromatography (*n*-hexane/EtOAc = 3:1) to give 2.85 g of **8** as a pale yellow oil (containing a small amount of an inseparable impurity, based on ^1H NMR analysis). The Weinreb's amide **8** was used for the next reaction without further purification.

To a stirred solution of dibromolefine **16** (14.7 g, 41.0 mmol, 3.1 equiv) in THF (165 mL) was added dropwise *n*-BuLi (1.6 M in hexane, 49.1 mL, 78.6 mmol, 5.86 equiv) at −78 °C. After 1 h of stirring, a solution of the Weinreb's amide **8** (2.85 g, ca. 13.4 mmol, 1.0 equiv) obtained above in THF (68 mL) was added to the mixture. After another 1.3 h of stirring at the same temperature, the mixture was allowed to warm gradually and stirred at −65 °C for 15.7 h and at −60 °C for 21 h. The mixture was cooled again to −78 °C and quenched with satd aq NH_4Cl (200 mL), and the layers were separated and extracted with Et_2O. The combined organic layers were washed with brine, dried (Na_2SO_4), and concentrated in vacuo. The residue was purified by SiO_2 column chromatography (*n*-hexane/EtOAc = 16:1) to give 3.85 g (66%) of ynone **7** as a pale yellow oil.

Specific Rotation: $[\alpha]_D^{25}$ +61.0 (*c* 0.960, $CHCl_3$)

^{1}H NMR (600 MHz, $CDCl_3$, ppm): δ = 6.38 (dd, *J* = 15.6, 10.2 Hz, 1H), 6.05 (dd, *J* = 15.0, 10.2 Hz, 1H), 5.80 (dt, *J* = 15.0, 6.6 Hz, 1H), 5.70 (d, *J* = 15.6 Hz, 1H), 4.05 (m, 1H), 3.81 (s, 1H), 2.59–2.52 (m, 2H), 2.11 (m, 2H), 1.54 (s, 3H), 1.26 (d, *J* = 6.0 Hz, 3H), 1.00 (t, *J* = 7.5 Hz, 3H), 0.89 (s, 9H), 0.08 (s, 6H)

^{13}C NMR (150 MHz, $CDCl_3$, ppm): δ=189.1, 138.4, 131.6, 130.3, 128.1, 98.6, 79.6, 78.9, 66.5, 30.1, 25.73, 25.70, 25.3, 23.5, 18.0, 13.3, –4.7, –4.9

IR (ATR): ν_{max} 3491 (br), 2960 (s), 2214 (m), 1672 (s) cm^{-1}

HRMS (ESI) *m*/*z*: $[M+Na]^+$ Calcd for $C_{20}H_{34}NaO_3Si$ 373.2169; Found 373.2168

(*R*)-4-bromo-5-((*R*)-2-((*tert*-butyldimethylsilyl)oxy)propyl)-2-((1*E*,3*E*)-hexa-1,3-dien-1-yl)-2-methylfuran-3(2*H*)-one (17)

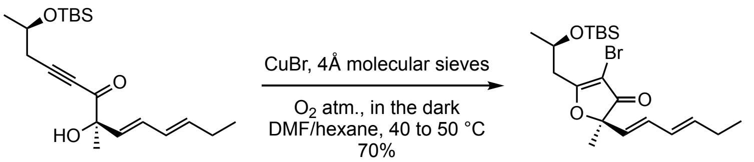

7 **17**

(*All procedures were performed in the dark.*) A solution of CuBr (19.1 mg, 133 μmol, 3.0 equiv) and 4Å molecular sieves (powder, 23.9 mg) in DMF (1.48 mL) was evacuated, and then filled with oxygen gas (repeated for 5 times). To this mixture was added a solution of ynone **7** (15.6 mg, 44.5 μmol) in *n*-hexane (2.96 mL) at room temperature. After 20 min of stirring at the same temperature, the mixture was heated using ChemiStation™ PPM-5512A at 40 °C for 35.3 h and at 50 °C for 13.7 h. After confirming almost all the starting material were consumed, the resulting mixture was filtered through a pad of Celite® and washed with EtOAc. The combined filtrates were washed with satd aq NH_4Cl, water and brine, dried (Na_2SO_4), and concentrated in vacuo. The residue was purified by preparative TLC (Merck silica gel 60 F254 plates, *n*-hexane/EtOAc = 16:1) to give 13.3 mg (70%) of bromide **17** as a white solid.

Mp: 41.6–42.4 °C

Specific Rotation: $[\alpha]_D^{25}$ +32.9 (*c* 0.570, $CHCl_3$)

^{1}H NMR (600 MHz, $CDCl_3$, ppm): δ = 6.27 (dd, *J* = 15.6, 10.2 Hz, 1H), 5.97 (dd, *J* = 15.0, 10.2 Hz, 1H), 5.81 (dt, *J* = 15.0, 6.6 Hz, 1H), 5.52 (d, *J* = 15.6 Hz, 1H), 4.30 (m, 1H), 2.91 (dd, *J* = 13.2, 6.6 Hz, 1H), 2.71 (dd, *J* = 13.2, 6.3 Hz, 1H), 2.10 (m, 2H), 1.53 (s, 3H), 1.25 (d, *J* = 6.6 Hz, 3H), 0.99 (t, *J* = 7.5 Hz, 3H), 0.87 (s, 9H), 0.08 (s, 3H), 0.05 (s, 3H)

^{13}C NMR (150 MHz, $CDCl_3$, ppm): δ = 197.9, 185.2, 139.5, 131.8, 127.7, 125.8, 96.3, 90.1, 66.1, 40.2, 25.74, 25.70, 24.0, 22.5, 18.0, 13.3, –4.4, –4.8

IR (ATR): ν_{max} 2960 (s), 2930 (s), 1719 (vs), 1595 (s) cm^{-1}

HRMS (ESI) *m*/*z*: $[M+Na]^+$ Calcd for $C_{20}H_{33}BrNaO_3Si$ 451.1275, 453.1255; Found 451.1275, 453.1256

methyl (*R*)-2-((*R*)-2-((*tert*-butyldimethylsilyl)oxy)propyl)-5-((1*E*,3*E*)-hexa-1,3-dien-1-yl)-5-methyl-4-oxo-4,5-dihydrofuran-3-carboxylate (18)

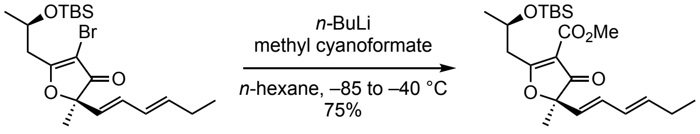

17 **18**

To a stirred suspension of bromide **17** (176 mg, 0.411 mmol) in hexane (10.3 mL) was added dropwise *n*-BuLi (1.53 M in hexane, 314 μL, 0.480 mmol, 1.17 equiv) at −85 °C under argon atmosphere. After stirring the mixture until all the precipitate was fully dissolved (around 8–9 min), this mixture was added to methyl cyanoformate [325 μL, 4.11 mmol, 10 equiv; stored with 4Å molecular sieves (pellets)] one portion. The resulting suspension was stirred at the same temperature for 2 h, then allowed to warm −40 °C before the mixture was quenched with satd aq NH_4Cl (5 mL) and diluted with EtOAc. The layers were separated and extracted with EtOAc. The combined organic layers were washed with brine, dried (Na_2SO_4), and concentrated in vacuo. The residue was purified by SiO_2 column chromatography (*n*-hexane/EtOAc = 10:1) to give 125 mg (75%) of ester **18** as a pale yellow oil.

Specific Rotation: $[\alpha]_D^{24}$ +71.7 (*c* 0.825, $CHCl_3$)

^{1}H NMR (600 MHz, $CDCl_3$, ppm): δ = 6.27 (dd, *J* = 15.6, 10.5 Hz, 1H), 5.95 (dd, *J* = 15.0, 10.5 Hz, 1H), 5.80 (dt, *J* = 15.0, 6.3 Hz, 1H), 5.52 (d, *J* = 15.6 Hz, 1H), 4.35 (m, 1H), 3.82 (s, 3H), 3.31 (dd, *J* = 13.2, 6.6 Hz, 1H), 3.04 (dd, *J* = 13.2,

6.0 Hz, 1H), 2.09 (m, 2H), 1.52 (s, 3H), 1.24 (d, J = 6.6 Hz, 3H), 0.98 (t, J = 7.5 Hz, 3H), 0.84 (s, 9H), 0.06 (s, 3H), 0.02 (s, 3H)

^{1}H NMR (600 MHz, benzene-d_6, ppm): δ = 6.42 (dd, J = 15.6, 10.8 Hz, 1H), 5.83 (dd, J = 15.3, 10.8 Hz, 1H), 5.55 (dt, J = 15.3, 6.6 Hz, 1H), 5.54 (d, J = 15.6 Hz, 1H), 4.31 (m, 1H), 3.50 (s, 3H), 3.30 (dd, J = 12.9, 6.3 Hz, 1H), 2.87 (dd, J = 12.9, 6.3 Hz, 1H), 1.85 (m, 2H), 1.37 (s, 3H), 1.11 (d, J = 6.0 Hz, 3H), 0.94 (s, 9H), 0.82 (t, J = 7.5 Hz, 3H), 0.08 (s, 3H), 0.06 (s, 3H)

^{13}C NMR (150 MHz, $CDCl_3$, ppm): δ = 198.3, 195.9, 163.2, 139.6, 132.1, 127.7, 125.4, 107.5, 91.3, 66.4, 51.6, 41.0, 25.72, 25.69, 24.1, 22.5, 17.9, 13.3, −4.5, −4.8

^{13}C NMR (150 MHz, benzene-d_6, ppm): δ = 196.5, 195.0, 163.6, 139.0, 132.0, 128.4, 126.7, 108.2, 91.0, 66.8, 51.0, 41.0, 25.94, 25.90, 24.0, 22.6, 18.1, 13.4, −4.5, −4.7

IR (ATR): ν_{max} 2958 (s), 2931 (s), 1714 (vs), 1589 (s) cm^{-1}

HRMS (ESI) m/z: $[M+Na]^+$ Calcd for $C_{22}H_{36}NaO_5Si$ 431.2224; Found 431.2227

gregatin A (6) and gregatin D (19)

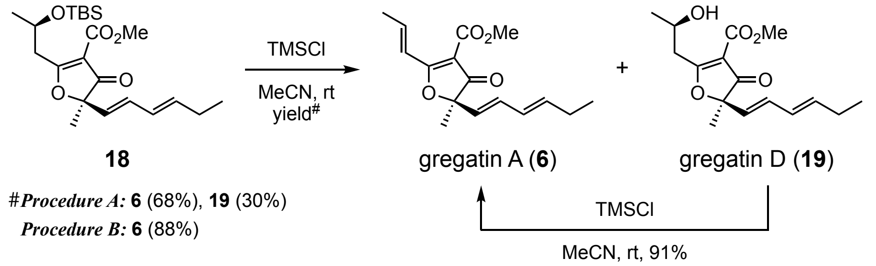

Procedure A: To a stirred suspension of silyl ether **18** (9.4 mg, 23 μmol) in MeCN (460 μL) was added dropwise TMSCl (4.4 μL, 35 μmol, 1.5 equiv) at room temperature. After 4 h of stirring at the same temperature, additional TMSCl (5.8 μL, 48 μmol, 2.0 equiv) was added to this mixture. The resulting mixture was stirred at the same temperature for 15.5 more hours, poured into a pH 7 phosphate buffer (ca. 10 mL) and then dilution with EtOAc. The layers were separated and extracted with EtOAc. The combined organic layers were washed with brine, dried (Na_2SO_4), and concentrated in vacuo. The residue was purified by preparative TLC (Merck silica gel 60 F254 plates, n-hexane/EtOAc = 2:1) to give 4.3 mg (68%) of gregatin A (**6**) as a white solid tother with 2.0 mg (30%) of gregatin D (**19**) as a colorless oil.

Procedure B: To a stirred suspension of silyl ether **18** (107 mg, 0.261 mmol) in MeCN (5.2 mL) was added dropwise TMSCl (99.0 μL, 0.780 mmol, 3.0 equiv) at room temperature. The resulting mixture was stirred at the same temperature for 29 h, during which time 99.0 μL (0.780 mmol, 3.0 equiv) and 60.6 μL (0.520 mmol, 2.0 equiv) of additional TMSCl were added to bring the elimination reaction to completion. The reaction mixture was poured into a pH 7 phosphate buffer (ca. 30 mL) and then diluted with EtOAc. The layers were separated and extracted with EtOAc. The combined organic layers were washed with brine, dried (Na_2SO_4), and concentrated in vacuo. The residue was purified by SiO_2 column chromatography (n-hexane/EtOAc = 7:1 to 4:1) to give 63.4 mg (88%) of gregatin A (**6**) as a white solid.

Conversion of gregatin D to gregatin A: To a stirred suspension of gregatin D (**19**) (4.7 mg, 16 μmol) in MeCN (320 μL) was added dropwise TMSCl (8.1 μL, 64 μmol, 4.0 equiv) at room temperature. After 22.3 h of stirring the solution at the same temperature, additional TMSCl (8.1 μL, 64 μmol, 4.0 equiv) was added to the mixture. The resulting mixture was stirred at the same temperature for another 4.8 h, poured into a pH 7 phosphate buffer (ca. 10 mL) and then diluted with EtOAc. The layers were separated and extracted with EtOAc. The combined organic layers were washed with brine, dried

(Na_2SO_4), and concentrated in vacuo. The residue was purified by preparative TLC (Merck silica gel 60 F254 plates, *n*-hexane/EtOAc = 2:1) to give 4.0 mg (91%) of gregatin A (**6**) as a white solid.

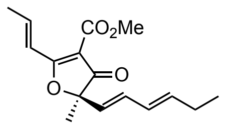

gregatin A (**6**)

Mp: 70.0–70.4 °C

Specific Rotation: $[\alpha]_D^{25}$ −170 (*c* 0.45, $CHCl_3$)

^{1}H NMR (600 MHz, $CDCl_3$, ppm): δ = 7.33 (dq, J = 15.6, 1.2 Hz, 1H), 7.20 (dq, J = 15.6, 6.9 Hz, 1H), 6.26 (dd, J = 15.6, 10.5 Hz, 1H), 5.97 (dd, J = 15.6, 10.5 Hz, 1H), 5.80 (dt, J = 15.6, 6.3 Hz, 1H), 5.56 (d, J = 15.6 Hz, 1H), 3.83 (s, 3H), 2.09 (m, 2H), 2.06 (dd, J = 6.9, 1.2 Hz, 3H), 1.54 (s, 3H), 0.98 (t, J = 7.5 Hz, 3H)

^{1}H NMR (600 MHz, benzene-d_6, ppm): δ = 7.53 (dq, J = 15.6, 1.8 Hz, 1H), 6.78 (dq, J = 15.6, 6.9 Hz, 1H), 6.40 (dd, J = 15.6, 10.8 Hz, 1H), 5.82 (dd, J = 15.0, 10.8 Hz, 1H), 5.56 (d, J = 15.6 Hz, 1H), 5.49 (dt, J = 15.0, 6.6 Hz, 1H), 3.53 (s, 3H), 1.84 (m, 2H), 1.38 (s, 3H), 1.35 (dd, J = 6.9, 1.8 Hz, 3H), 0.81 (t, J = 7.5 Hz, 3H)

^{13}C NMR (150 MHz, $CDCl_3$, ppm): δ = 198.4, 185.3, 163.5, 144.9, 139.3, 131.5, 127.7, 126.1, 120.7, 103.6, 90.5, 51.7, 25.7, 22.5, 19.4, 13.3

^{13}C NMR (150 MHz, benzene-d_6, ppm): δ = 196.7, 184.7, 163.8, 143.3, 138.7, 131.4, 128.4, 127.4, 121.2, 104.6, 90.2, 51.0, 25.9, 22.5, 18.7, 13.4

IR (ATR): ν_{max} 2966 (w), 1707 (vs), 1645 (s), 1556 (s) cm^{-1}

HRMS (ESI) *m/z*: $[M+Na]^+$ Calcd for $C_{16}H_{20}NaO_4$ 299.1254; Found 299.1255

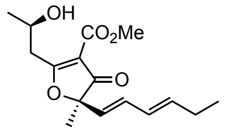

gregatin D (**19**)

Specific Rotation: $[\alpha]_D^{24}$ +100 (*c* 0.24, $CHCl_3$)

^{1}H NMR (600 MHz, $CDCl_3$, ppm): δ = 6.30 (dd, J = 15.3, 10.2 Hz, 1H), 5.97 (dd, J = 15.0, 10.2 Hz, 1H), 5.81 (dt, J = 15.0, 6.6 Hz, 1H), 5.54 (d, J = 15.3 Hz, 1H), 4.31 (m, 1H), 3.84 (s, 3H), 3.20 (dd, J = 13.2, 7.8 Hz, 1H), 3.16 (dd, J = 13.2, 4.5 Hz, 1H), 2.47 (d, J = 5.4 Hz, 1H), 2.09 (m, 2H), 1.54 (s, 3H), 1.35 (d, J = 6.6 Hz, 3H), 0.98 (t, J = 7.2 Hz, 3H)

^{13}C NMR (150 MHz, $CDCl_3$, ppm): δ = 197.9, 195.9, 164.2, 139.8, 132.1, 127.6, 125.2, 107.7, 91.8, 66.3, 51.9, 40.3, 25.7, 24.0, 22.4, 13.3

IR (ATR): ν_{max} 3470 (br), 2971 (w), 1711 (vs), 1583 (s) cm^{-1}

HRMS (ESI) *m/z*: $[M+Na]^+$ Calcd for $C_{16}H_{22}NaO_5$ 317.1359; Found 317.1355

(5*S*,6*R*)-5,6-dimethyltetrahydro-2*H*-pyran-2-one (15)

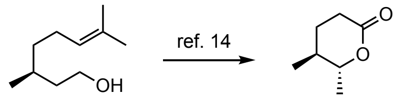

(–)-β-citronellol **15**

Known lactone **15** was prepared from (−)-β-citronellol (>92% *ee,* purchased from Tokyo Chemical Industry Co., Ltd.) by following the literature procedure cited in reference 14 in the text. **15** was obtained as a colorless oil. The analytical data of **15** were identical with those reported in the reference 14.

Specific Rotation: $[\alpha]_D^{21}$ +26.9 (*c* 0.745, $CHCl_3$).

^{1}H NMR (600 MHz, $CDCl_3$, ppm): δ = 4.06 (dq, *J* = 9.6, 6.6 Hz, 1H), 2.63 (ddd, *J* = 18.0, 6.9, 3.9 Hz, 1H), 2.49 (ddd, *J* = 18.0, 9.9, 7.2 Hz, 1H), 1.90 (m, 1H), 1.67–1.51 (m, 2H), 1.37 (d, *J* = 6.6 Hz, 3H), 1.00 (d, *J* = 6.6, 3H)

(5*S*,6*R*)-5,6-dimethyl-3-(methylthio)tetrahydro-2*H*-pyran-2-one (SI-4)

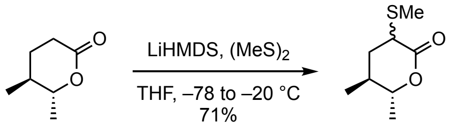

To a stirred solution of LiHMDS (1.3 M in THF, 20.4 mL, 26.5 mmol, 2.0 equiv) was added dropwise a solution of lactone **15** (1.72 g, 13.4 mmol) in THF (135 mL) at −78 °C. After 30 min of stirring at the same temperature, a solution of dimethyl disulfide (2.61 mL, 29.5 mmol, 2.2 equiv) in THF (30 mL) was added dropwise to the mixture. The resulting mixture was stirred at the same temperature for 20 min, then allowed to warm to −20 °C before the mixture was quenched with satd aq NH_4Cl (100 mL) and diluted with Et_2O. The layers were separated and extracted with Et_2O. The combined organic layers were washed with brine, dried (Na_2SO_4), and concentrated in vacuo. The resulting residue was purified by SiO_2 column chromatography (*n*-hexane/EtOAc = 7:2) to give 1.65 g (71%) of sulfide **SI-4** as a mixture of diastereomers (*dr* 6:5 based on ^{1}H NMR measurement; separable, stereochemistry undetermined) as a pale yellow oil.

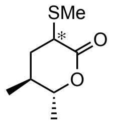

SI-4a (major diasteromer)

Specific Rotation: $[\alpha]_D^{23}$ −45 (*c* 0.48, $CHCl_3$)

^{1}H NMR (600 MHz, $CDCl_3$, ppm): δ = 4.04 (dq, *J* = 9.6, 6.6 Hz, 1H), 3.42 (m, 1H), 2.28 (s, 3H), 2.01–1.91 (m, 3H), 1.39 (d, *J* = 6.6 Hz, 3H), 0.98 (d, *J* = 6.6 Hz, 3H)

^{13}C NMR (150 MHz, $CDCl_3$, ppm): δ = 169.9, 83.2, 43.3, 34.9, 31.6, 19.8, 17.3, 16.2

IR (ATR): ν_{max} 2979 (w), 2925 (w), 1725 (s), 1105 (m) cm^{-1}

HRMS (ESI) *m*/*z*: $[M+Na]^+$ Calcd for $C_8H_{14}NaO_2S$ 197.0607; Found 197.0605

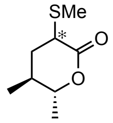

SI-4b (minor diasteromer)

Specific Rotation: $[\alpha]_D^{23}$ +110 (*c* 0.400, $CHCl_3$)

^{1}H NMR (600 MHz, $CDCl_3$, ppm): δ = 4.32 (dq, *J* = 10.2, 6.0 Hz, 1H), 3.43 (dd, *J* = 8.4, 6.6 Hz, 1H), 2.39 (ddd, *J* = 14.4, 8.4, 6.0 Hz, 1H), 2.25 (s, 3H), 1.70 (m, 1H), 1.45 (ddd, *J* = 14.4, 10.8, 6.6 Hz, 1H), 1.35 (d, *J* = 6.0 Hz, 3H), 0.98 (d, *J* = 6.6 Hz, 3H)

^{13}C NMR (150 MHz, $CDCl_3$, ppm): δ = 170.0, 80.8, 42.4, 34.94, 34.91, 19.4, 17.2, 15.8

IR (ATR): ν_{max} 2978 (w), 2922 (w), 1728 (s), 1102 (m) cm^{-1}

HRMS (ESI) *m/z*: $[M+Na]^+$ Calcd for $C_8H_{14}NaO_2S$ 197.0607; Found 197.0608

(5*S*,6*R*)-5,6-dimethyl-3-(methylthio)-5,6-dihydro-2*H*-pyran-2-one (20)

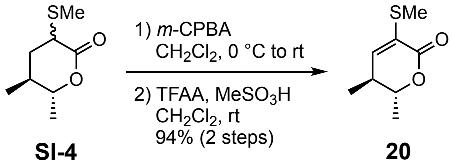

SI-4 *dr* 6:5 → **20**

To a stirred solution of sulfide **SI-4** (1.60 g, 9.18 mmol, a 6:5 diastereomeric mixture) in CH_2Cl_2 (91 mL) was added portionwise *m*-CPBA [68.5% (w/w), 2.20 g, 8.73 mmol, 0.95 equiv] at 0 °C. After 10 min of stirring at the same temperature, the mixture was allowed to warm to room temperature. The mixture was stirred at the same temperature for 25 min before the mixture was quenched with satd aq $NaHCO_3$ (50 mL) and 10% (w/w) aq Na_2SO_3 (50 mL). The layers were separated and extracted with CH_2Cl_2. The combined organic layers were washed with brine, dried (Na_2SO_4), and concentrated in vacuo. The resulting crude intermediary sulfoxide was used directly for the next reaction without further purification.

To a stirred solution of the sulfoxide obtained above in CH_2Cl_2 (91 mL) were added dropwise TFAA (3.20 mL, 23.0 mmol 2.5 equiv) and methanesulfonic acid (120 μL, 1.85 mmol 0.2 equiv) successively at room temperature. The resulting mixture was stirred at the same temperature for 25 min, then cooled to 0 °C before the mixture was quenched with satd aq $NaHCO_3$ (80 mL). The layers were separated and extracted with CH_2Cl_2. The combined organic layers were washed with brine, dried (Na_2SO_4), and concentrated in vacuo. The residue was purified by SiO_2 column chromatography (*n*-hexane/EtOAc = 12:1) to give 1.49 g (94%) of sulfide **20** as a pale pink solid.

Mp: 68.5–69.3 °C

Specific Rotation: $[\alpha]_D^{21}$ −52.0 (*c* 1.20, $CHCl_3$)

^{1}H NMR (600 MHz, $CDCl_3$, ppm): δ = 6.04 (d, *J* = 2.4 Hz, 1H), 4.17 (dq, *J* = 10.2, 6.6 Hz, 1H), 2.49 (ddq, *J* = 10.2, 2.4, 7.2 Hz, 1H), 2.25 (s, 3H), 1.41 (d, *J* = 6.6 Hz, 3H), 1.13 (d, *J* = 7.2 Hz, 3H)

^{13}C NMR (150 MHz, $CDCl_3$, ppm): δ = 162.8, 138.6, 130.0, 80.4, 36.4, 18.9, 16.7, 14.3

IR (ATR): ν_{max} 3734 (w), 2979 (w), 1716 (vs), 1224 (m) cm^{-1}

HRMS (ESI) *m/z*: $[M+Na]^+$ Calcd for $C_8H_{12}NaO_2S$ 195.0450; Found 195.0446

(5*S*,6*R*)-5,6-dimethyl-3-(methylsulfinyl)-5,6-dihydro-2*H*-pyran-2-one (21)

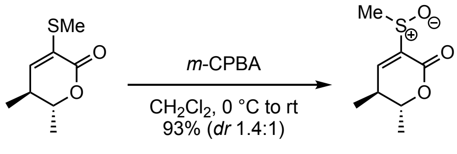

20 → **21**

Procedure A: To a stirred solution of sulfide **20** (14.4 mg, 83.6 μmol) in CH_2Cl_2 (560 μL) was added portionwise *m*-CPBA [68.5% (w/w), 20.5 mg, 79.6 μmol, 0.97 equiv] at 0 °C. After 5 min of stirring at the same temperature, this mixture was allowed to warm to room temperature. The resulting mixture was stirred at the same temperature for 20 min before the mixture was quenched with satd aq $NaHCO_3$ (1 mL) and 10% (w/w) aq Na_2SO_3 (1 mL). The layers were separated and extracted with CH_2Cl_2. The combined organic layers were washed with brine, dried (Na_2SO_4), and concentrated in vacuo.

The residue was purified by preparative TLC (Merck silica gel 60 F254 plates, *n*-hexane/EtOAc = 1:2) to give 14.6 mg (93%) of an inseparable 1.4:1 diastereomeric mixture of **21** (based on ^{1}H NMR measurement) as a colorless oil.

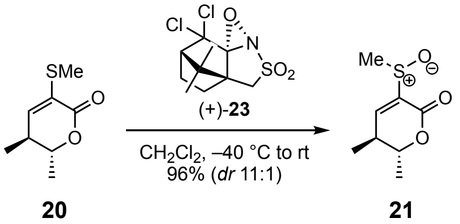

Procedure B: To a stirred solution of sulfide **20** (697 mg, 4.05 mmol) in CH_2Cl_2 (13.5 mL) was added dropwise a solution of (+)-(8,8-dichlorocamphorylsulfonyl)oxaziridine [(+)-**23**, 1.45 g, 4.86 mmol, 1.2 equiv] in CH_2Cl_2 (5 mL) at –40 °C. After 13.4 h of stirring at the same temperature, the mixture was allowed to warm gradually and stirred at –20 °C for 4 h, at 0 °C for 2 h and at room temperature for 4.8 h. The reaction mixture was directly purified by SiO_2 column chromatography (the reaction mixture was loaded onto SiO_2 packed in column with CH_2Cl_2 and left to stand for 1 h, and then eluted with *n*-hexane/EtOAc = 1:1 to 0:100) to give 732 mg (96%) of an inseparable 11:1 diastereomeric mixture of **21** (based on ^{1}H NMR measurement) as a colorless oil.

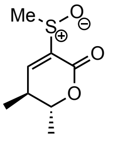

21 (*dr* 11:1)

Specific Rotation: $[\alpha]_D^{22}$ +24.8 (*c* 1.31, $CHCl_3$)

^{1}H NMR (600 MHz, CD_2Cl_2, ppm): δ = 7.28 (d, J = 2.4 Hz, 1/12H), 7.19 (d, J = 1.8 Hz, 11/12H), 4.30 (dq, J = 9.6, 6.0 Hz, 1/12H), 4.25 (dq, J = 10.2, 6.0 Hz, 11/12H), 2.77 (s, 1/12×3H), 2.71 (s, 11/12×3H), 2.68–2.63 (m, 1H), 1.46–1.44 (d, J = 6.0 Hz, 3H), 1.24–1.21 (d, J = 7.8 Hz, 3H)

^{13}C NMR (150 MHz, CD_2Cl_2, ppm): δ = 161.3, 151.3, 138.0, 81.1, 40.8, 36.6, 19.0, 16.0

IR (ATR): ν_{max} 2981 (w), 1713 (vs), 1209 (m), 1060 (m) cm^{-1}

HRMS (ESI) *m/z*: [M+Na]$^+$ Calcd for $C_8H_{12}NaO_3S$ 211.0399; Found 211.0400

(3*R*,4*S*)-8-hydroxy-6-methoxy-3,4,5-trimethylisochroman-1-one (22) and (3*R*,4*S*)-6,8-dimethoxy-3,4,5-trimethylisochroman-1-one (22´)

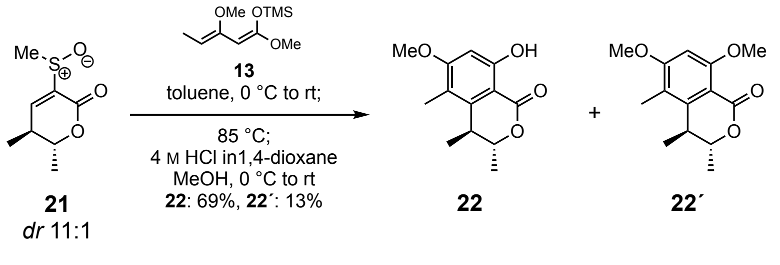

To a stirred solution of methyl sulfoxide **21** (376 mg, 2.00 mmol, *dr* 11:1) in toluene (10 mL) was added dropwise diene **13** (940 μL, 4.00 mmol, 2.0 equiv) at 0 °C. After 30 min of stirring at the same temperature, the mixture was allowed to warm to room temperature. The resulting mixture was stirred at room temperature for 39 h, during which time 470 μL (2.00 mmol, 1.0 equiv) of additional diene **13** was added twice to bring the Diels–Alder reaction to completion. The reaction mixture was diluted with toluene (3.0 mL) and heated using oil bath at 85 °C for 1.5 h. The resulting mixture was

cooled to 0 °C before 4 M HCl in 1,4-dioxane (500 μL, 2.00 mmol, 1.0 equiv) and MeOH (121 μL, 2.99 mmol, 1.5 equiv) was added to the mixture. After 2 h of stirring at room temperature, the mixture was added water (20 mL), and the layers were separated and extracted with EtOAc. The combined organic layers were washed with brine, dried (Na_2SO_4), and concentrated in vacuo. The residue was purified by SiO_2 column chromatography (*n*-hexane/EtOAc =8:1 to 1:4) to give 324 mg (69%) of **22** as a white solid and 63.5 mg (13%) of **22´** as a white solid.

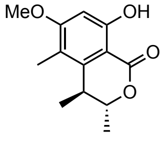

22

Mp: 92.5–93.2 °C

Specific Rotation: $[\alpha]_D^{23}$ +96 (*c* 0.48, $CHCl_3$)

^{1}H NMR (600 MHz, $CDCl_3$, ppm): δ = 11.47 (s, 1H), 6.36 (s, 1H), 4.67 (seemingly dq, J = 0.6, 6.6 Hz, 1H), 3.85 (s, 3H), 2.97 (seemingly dq, J = 0.6, 7.2 Hz, 1H), 2.05 (s, 3H), 1.32 (d, J = 6.6 Hz, 3H), 1.29 (d, J = 7.2 Hz, 3H)

^{13}C NMR (150 MHz, $CDCl_3$, ppm): δ = 168.7, 164.4, 162.9, 141.6, 115.2, 99.4, 97.3, 80.0, 55.7, 34.7, 20.0, 19.7, 10.0

IR (ATR): ν_{max} 3649 (m), 1661 (s), 1238 (m), 1156 (m) cm^{-1}

HRMS (ESI) *m*/*z*: $[M+Na]^+$ Calcd for $C_{13}H_{16}NaO_4$ 259.0941; Found 259.0938

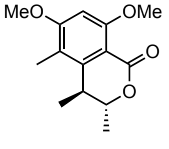

22´

Mp: 124.4–125.0 °C

Specific Rotation: $[\alpha]_D^{23}$ +180 (*c* 0.45, $CHCl_3$)

^{1}H NMR (600 MHz, $CDCl_3$, ppm): δ = 6.39 (s, 1H), 4.55 (q, J = 6.6 Hz, 1H), 3.94 (s, 3H), 3.90 (s, 3H), 2.96 (q, J = 7.2 Hz, 1H), 2.09 (s, 3H), 1.31 (d, J = 7.2 Hz, 3H), 1.23 (d, J = 6.6 Hz, 3H)

^{13}C NMR (150 MHz, $CDCl_3$, ppm): δ = 162.5, 161.6, 161.3, 144.4, 115.3, 105.0, 93.7, 77.7, 56.1, 55.5, 35.5, 19.9, 19.2, 10.1

IR (ATR): ν_{max} 2977 (w), 1715 (s), 1144 (m), 1103 (m) cm^{-1}

HRMS (ESI) *m*/*z*: $[M+Na]^+$ Calcd for $C_{14}H_{18}NaO_4$ 273.1097; Found 273.1101

stoloniferol B (11)

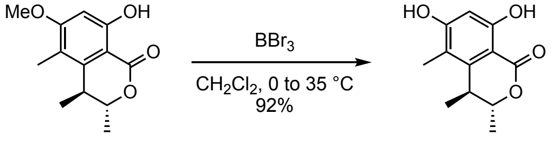

22 stoloniferol B (**11**)

***Conversion of* 22 *into* 11**: To a stirred solution of **22** (100 mg, 0.425 mmol) in CH_2Cl_2 (4.3 mL) was added dropwise boron tribromide (1.0 M in CH_2Cl_2, 2.12 mL, 2.12 mmol, 5.0 equiv) at 0 °C. After 10 min of stirring at the same temperature, the mixture was heated using ChemiStation™ PPM-5512A at 35°C for 10.5 h. The resulting mixture was cooled to 0 °C before the mixture was quenched with satd aq $NaHCO_3$ (5 mL). The layers were separated and extracted with CH_2Cl_2. The combined organic layers were washed with brine, dried (Na_2SO_4), and concentrated in vacuo. The residue was purified by

SiO_2 column chromatography (*n*-hexane/EtOAc = 3:1) to give 87.1 mg (92%) of stoloniferol B (**11**) as a pale yellow solid.

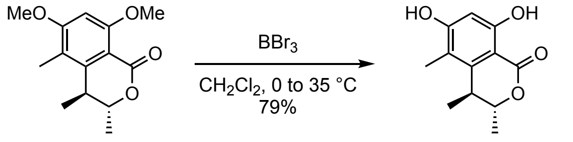

22´ stoloniferol B (**11**)

***Conversion of* 22´ *into* 11**: To a stirred solution of **22´** (63.5 mg, 0.254 mmol) in CH_2Cl_2 (2.5 mL) was added dropwise boron tribromide (1.0 M in CH_2Cl_2, 2.03 mL, 2.03 mmol, 8.0 equiv) at 0 °C. After 10 min of stirring at the same temperature, the mixture was heated using ChemiStation™ PPM-5512A at 35°C for 9.5 h. The resulting mixture was cooled to 0 °C before the mixture was quenched with satd aq $NaHCO_3$ (3 mL). The layers were separated and extracted with CH_2Cl_2. The combined organic layers were washed with brine, dried (Na_2SO_4), and concentrated in vacuo. The residue was purified by SiO_2 column chromatography (*n*-hexane/EtOAc = 15:1 to 1:3) to give 44.5 mg (79%) of stoloniferol B (**11**) as a pale yellow solid.

Mp: 214.0–214.8 °C

Specific Rotation: $[\alpha]_D^{23}$ +120 (*c* 0.43, $CHCl_3$)

^{1}H NMR (600 MHz, $CDCl_3$, ppm): δ = 11.36 (s, 1H), 6.29 (s, 1H), 5.50 (s, 1H), 4.68 (seemingly dq, *J* = 0.6, 6.9 Hz, 1H), 2.97 (q, *J* = 7.2 Hz, 1H), 2.10 (s, 3H), 1.33 (d, *J* = 6.9 Hz, 3H), 1.31 (d, *J* = 7.2 Hz, 3H)

^{13}C NMR (150 MHz, $CDCl_3$, ppm): δ = 168.7, 162.3, 161.1, 143.0, 113.5, 101.4, 100.2, 80.1, 34.8, 20.0, 19.7, 9.9

IR (ATR): ν_{max} 3274 (br), 2978 (w), 1647 (s), 1395 (m) cm^{-1}

HRMS (ESI) *m*/*z*: $[M+Na]^+$ Calcd for $C_{12}H_{14}NaO_4$ 245.0784; Found 245.0787

(3*R*,4*S*)-1-hydroxy-3,4,5-trimethylisochromane-6,8-diyl bis(2-(trimethylsilyl)ethane-1-sulfonate) (24)

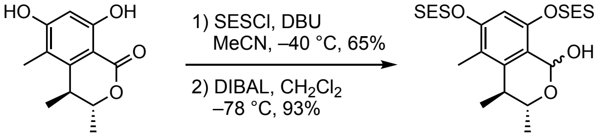

stoloniferol B (**11**) **24**

To a stirred solution of stoloniferol B (**11**) (99.6 mg, 0.448 mmol) in MeCN (4.5 mL) were added successively DBU (161 μL, 1.12 mmol, 2.5 equiv) and 2-(trimethylsilyl)ethanesulfonyl chloride (230 μL, 1.17 mmol, 2.6 equiv) at −40 °C. After 1 h of stirring at the same temperature, the mixture was quenched with satd aq NH_4Cl (3 mL). The layers were separated and extracted with CH_2Cl_2. The combined organic layers were washed with brine, dried (Na_2SO_4), and concentrated in vacuo. The residue was purified by SiO_2 column chromatography (*n*-hexane/EtOAc = 7:2) to give 160 mg (65%) of the bis-sulfonate as a white amorphas solid.

To a stirred solution of the bis-sulfonate obtained above (160 mg, 0.290 mmol) in CH_2Cl_2 (2.9 mL) was added dropwise DIBAL (1.0 M in hexane, 580 μL, 0.580 mmol, 2.0 equiv) at −78 °C. After 30 min of stirring at the same temperature, the mixture was quenched with satd aq Rochelle salt (6 mL) and warmed to room temperature. After vigorously stirring the resulting mixture at the same temperature until it completely separated into two layers, the layers were then separated and extracted with EtOAc. The combined organic layers were washed with brine, dried (Na_2SO_4), and concentrated in vacuo to give 149 mg (93%) of an inseparable 7:3 diastereomeric mixture of lactol **24** (based on ^{1}H NMR measurement) as a pale yellow wax.

Specific Rotation: $[\alpha]_D^{27}$ −21.8 (*c* 0.635, $CHCl_3$)

^{1}H NMR (600 MHz, $CDCl_3$, ppm): δ = 7.27 (s, 3/10H), 7.20 (s, 7/10H), 6.14 (d, *J* = 3.6 Hz, 7/10H), 6.12 (d, *J* = 4.2 Hz,

3/10H), 4.24 (m, 7/10H), 4.17 (q, J = 6.9 Hz, 3/10H), 3.39–3.27 (m, 47/10H), 3.13 (d, J = 4.2 Hz, 3/10H), 2.79 (q, J = 7.2 Hz, 3/10H), 2.75 (m, 7/10H), 2.30 (s, 3H), 1.38 (d, J = 7.2 Hz, 3/10×3H), 1.36 (d, J = 6.6 Hz, 7/10×3H), 1.27 (d, J = 6.6 Hz, 7/10×3H), 1.25–1.17 (m, 49/10H), 0.10 (s, 9H), 0.08 (s, 7/10×9H), 0.07 (s, 3/10×9H)

^{13}C NMR (150 MHz, $CDCl_3$, ppm): δ = 147.8, 143.7, 142.1, 129.8, 127.3, 114.4, 87.5, 69.9, 48.4, 48.2, 36.5, 20.2, 19.2, 13.4, 10.4, 10.3, –2.0 (2C)

IR (ATR): ν_{max} 2953 (m), 1364 (s), 1180 (s), 835 (s) cm^{-1}

HRMS (ESI) *m*/*z*: [M+Na]$^+$ Calcd for $C_{22}H_{40}NaO_8S_2Si_2$ 575.1595; Found 575.1593

(3*R*,4*S*)-7-bromo-6,8-bis(methoxymethoxy)-3,4,5-trimethylisochroman-1-one (25)

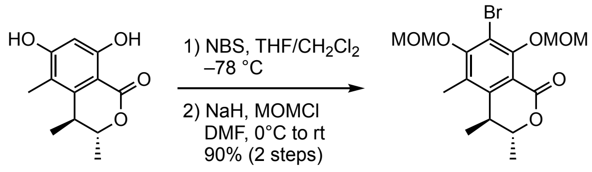

stoloniferol B (**11**) **25**

To a stirred solution of stoloniferol B (**11**) (19.5 mg, 87.7 μmol) in CH_2Cl_2 (880 μL) was added dropwise a solution of *N*-bromosuccinimide (17.2 mg, 96.6 μmol, 1.1 equiv) in THF (880 μL) at −78 °C. After 50 min of stirring at the same temperature, the mixture was quenched with 10% (w/w) aq $Na_2S_2O_3$ (1 mL) and diluted with CH_2Cl_2. The layers were separated and extracted with CH_2Cl_2. The combined organic layers were washed with brine, dried (Na_2SO_4), and concentrated in vacuo. The resulting crude bromide was used directly for the next reaction without further purification.

To a stirred suspension of NaH (60% mineral oil dispersion, 10.5 mg, 263 μmol, 3.0 equiv) in DMF (440 μL) was added dropwise a solution of the intermediary bromide obtained above in DMF (880 μL) at 0 °C. After 30 min of stirring the solution at the same temperature, MOMCl (16.5 μL, 219 μmol, 2.5 equiv) was added dropwise to the mixture. After 15 min of stirring at the same temperature, the mixture was allowed to warm to room temperature and stirred for 15 min. The resulting mixture was cooled to 0 °C before the mixture was quenched with satd aq NH_4Cl (3 mL). The layers were separated and extracted with EtOAc. The combined organic layers were washed with brine, dried (Na_2SO_4), and concentrated in vacuo. The residue was purified by SiO_2 column chromatography (*n*-hexane/EtOAc = 2:1) to give 30.8 mg (90%) of bis-MOM ether **25** as a colorless oil.

Specific Rotation: $[\alpha]_D^{24}$ +136 (*c* 0.855, $CHCl_3$)

^{1}H NMR (600 MHz, $CDCl_3$, ppm): δ = 5.18 (d, J = 6.0 Hz, 1H), 5.16 (d, J = 6.3 Hz, 1H), 5.12 (d, J = 6.3 Hz, 1H), 5.10 (d, J = 6.0 Hz, 1H), 4.62 (seemingly dq, J = 0.9, 6.6 Hz, 1H), 3.66 (s, 3H), 3.65 (s, 3H), 2.96 (seemingly dq, J = 0.9, 7.2 Hz, 1H), 2.30 (s, 3H), 1.34 (d, J = 7.2 Hz, 3H), 1.27 (d, J = 6.6 Hz, 3H)

^{13}C NMR (150 MHz, $CDCl_3$, ppm): δ = 160.6, 158.5, 155.3, 143.1, 126.8, 115.6, 114.0, 101.2, 100.1, 78.3, 58.3, 58.1, 35.9, 19.9, 18.9, 12.4

IR (ATR): ν_{max} 2978 (m), 1722 (s), 1160 (m), 928 (m) cm^{-1}

HRMS (ESI) *m*/*z*: [M+Na]$^+$ Calcd for $C_{16}H_{21}BrNaO_6$ 411.0414, 413.0394; Found 411.0401, 413.0381

tert-butyl (3R,4S)-6,8-bis(methoxymethoxy)-3,4,5-trimethyl-1-oxoisochromane-7-carboxylate (26)

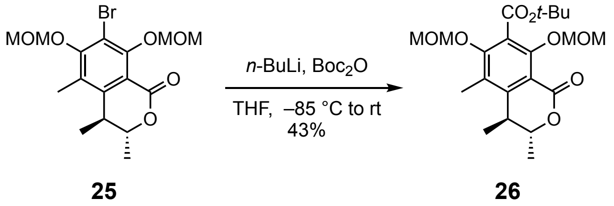

To a stirred solution of bromide **25** (11.3 mg, 29.0 μmol) in THF (970 μL) was added dropwise *n*-BuLi (1.34 M in hexane, 22.8 μL, 30.6 μmol, 1.05 equiv) at –85 °C under argon atmosphere. After 15 min of stirring the solution at the same temperature, a solution of Boc_2O (8.70 μL, 37.8 μmol, 1.3 equiv) in THF (37 μL) was added dropwise to the mixture. After another 30 min of stirring at the same temperature, the mixture was allowed to warm to room temperature. The resulting mixture was quenched with satd aq NH_4Cl (1 mL). The layers were separated and extracted with EtOAc. The combined organic layers were washed with water and brine, dried (Na_2SO_4), and concentrated in vacuo. The residue was purified by preparative TLC (Merck silica gel 60 F254 plates, *n*-hexane/EtOAc = 7:5) to give 5.1 mg (43%) of *tert*-butyl ester **26** as a colorless oil.

Specific Rotation: $[\alpha]_D^{25}$ +127 (*c* 0.550, $CHCl_3$)

^{1}H NMR (600 MHz, $CDCl_3$, ppm): δ = 5.14 (d, *J* = 5.4 Hz, 1H), 5.10 (d, *J* = 5.4 Hz, 1H), 5.07 (m, 2H), 4.61 (q, *J* = 6.6 Hz, 1H), 3.59 (s, 3H), 3.53 (s, 3H), 2.97 (q, *J* = 7.2 Hz, 1H), 2.25 (s, 3H), 1.59 (s, 9H), 1.33 (d, *J* = 7.2 Hz, 3H), 1.28 (d, *J* = 6.6 Hz, 3H)

^{13}C NMR (150 MHz, $CDCl_3$, ppm): δ = 164.8, 160.9, 157.2, 154.4, 144.8, 126.0, 125.5, 114.5, 101.4, 100.8, 83.0, 78.3, 58.1, 57.8, 36.0, 28.1, 19.9, 18.9, 11.8

IR (ATR): ν_{max} 2978 (w), 1721 (m), 1228 (w), 1159 (s) cm^{-1}

HRMS (ESI) *m/z*: $[M+Na]^+$ Calcd for $C_{21}H_{30}NaO_8$ 433.1833; Found 433.1830

tert-butyl (3R,4S)-1-hydroxy-6,8-bis(methoxymethoxy)-3,4,5-trimethylisochromane-7-carboxylate (SI-5)

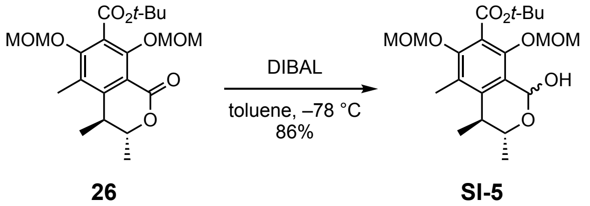

To a stirred suspension of lactone **26** (39.1 mg, 95.3 μmol) in toluene (3.0 mL) was added dropwise DIBAL (1.0 M in hexane, 220 μL, 220 μmol, 2.3 equiv) at –78 °C under argon atmosphere. After 25 min of stirring at the same temperature, the mixture was quenched with satd aq Rochelle salt (3 mL) and warmed to room temperature. After vigorously stirring the resulting mixture at the same temperature until it completely separated into two layers, the layers were then separated and extracted with EtOAc. The combined organic layers were washed with brine, dried (Na_2SO_4), and concentrated in vacuo. The residue was purified by SiO_2 column chromatography (toluene/EtOAc = 5:1) to give 33.6 mg (86%) of an inseparable 4:1 diastereomeric mixture of lactol **SI-5** (based on ^{1}H NMR measurement) as a colorless oil.

Specific Rotation: $[\alpha]_D^{22}$ −15 (*c* 0.49, $CHCl_3$)

^{1}H NMR (600 MHz, $CDCl_3$, ppm): δ = 6.16 (d, *J* = 2.7 Hz, 4/5H), 6.14 (d, *J* = 2.4 Hz, 1/5H), 5.11–5.06 (m, 2H), 5.03–4.99 (m, 2H), 4.25 (m, 4/5H), 4.14 (q, *J* = 7.2 Hz, 1/5H), 3.99 (d, *J* = 2.7 Hz, 4/5H), 3.79 (d, *J* = 2.4 Hz, 1/5H), 3.59 (s, 3H), 3.58 (s, 3H), 2.74–2.68 (m, 1H), 2.24 (s, 3H), 1.59 (s, 1/5×9H), 1.58 (s, 4/5×9H), 1.37 (d, *J* = 7.2 Hz, 1/5×3H), 1.34 (d, *J* = 6.6 Hz, 4/5×3H), 1.25 (d, *J* = 6.6 Hz, 4/5×3H), 1.19 (d, *J* = 7.2 Hz, 1/5×3H)

^{13}C NMR (150 MHz, $CDCl_3$, ppm): δ = 165.6, 153.3, 150.0, 141.1, 127.2, 126.6, 123.1, 101.1, 100.5, 87.8, 82.5, 69.9, 57.7, 57.6, 36.7, 28.1, 20.5, 19.3, 13.1

IR (ATR): ν_{max} 3456 (br), 2978 (m), 1722 (m), 1156 (s) cm^{-1}

HRMS (ESI) *m/z*: $[M+Na]^+$ Calcd for $C_{21}H_{32}NaO_8$ 435.1989; Found 435.1989

citrinin (5)

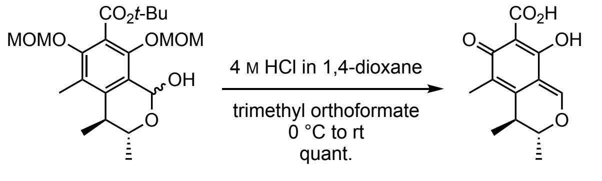

To a stirred solution of lactol **SI-5** (32.4 mg, 78.5 μmol, *dr* 4:1) in trimethyl orthoformate (860 μL, 7.86 mmol, 100 equiv) was added dropwise 4 M HCl in dioxane (1.96 mL, 7.84 mmol, 100 equiv) at 0 °C, and then allowed to warm to room temperature (color of the reaction mixture changed to bright yellow). The resulting mixture was stirred at the same temperature until the TLC displayed full consumption of the starting material (around 2.5 h). After diluting the reaction mixture with toluene (ca. 10 mL), it was transferred to a round-bottom flask, and nitrogen gas was purged for 7 minutes to eliminate HCl. Following the confirmation that the pH of the residue became 7, the solvents were removed under reduced pressure to give crude citrinin (**5**) (containing a trace amount of an impurity based on ^{1}H NMR measurement), to which MeOH and *n*-hexane were added. After the layers were separated, the *n*-hexane layer was extracted with MeOH until it became completely colorless. The combined MeOH layers were washed once with *n*-hexane and then concentrated in vacuo to give 19.7 mg (quant) of pure citrinin (**5**) as a lemon yellow solid.

Mp: 160.8–162.2 °C (dec.)

Specific Rotation: $[\alpha]_D^{22}$ +19.7 (*c* 0.500, $CHCl_3$)

^{1}H NMR (600 MHz, $CDCl_3$, ppm): δ = 15.87 (s, 1H), 15.11 (s, 1H), 8.23 (s, 1H), 4.77 (q, J = 6.6 Hz, 1H), 2.98 (q, J = 7.2 Hz, 1H), 2.02 (s, 3H), 1.34 (d, J = 6.6 Hz, 3H), 1.23 (d, J = 7.2 Hz, 3H)

^{13}C NMR (150 MHz, $CDCl_3$, ppm): δ = 183.8, 177.2, 174.5, 162.8, 139.0, 123.1, 107.4, 100.3, 81.7, 34.6, 18.5, 18.2, 9.5

IR (ATR): ν_{max} 2975 (w), 2931 (w), 1629 (vs), 1508 (vs) cm^{-1}

HRMS (ESI) *m/z*: $[M+Na]^+$ Calcd for $C_{13}H_{14}NaO_5$ 273.0733; Found 273.0736

citrifuran D (2)

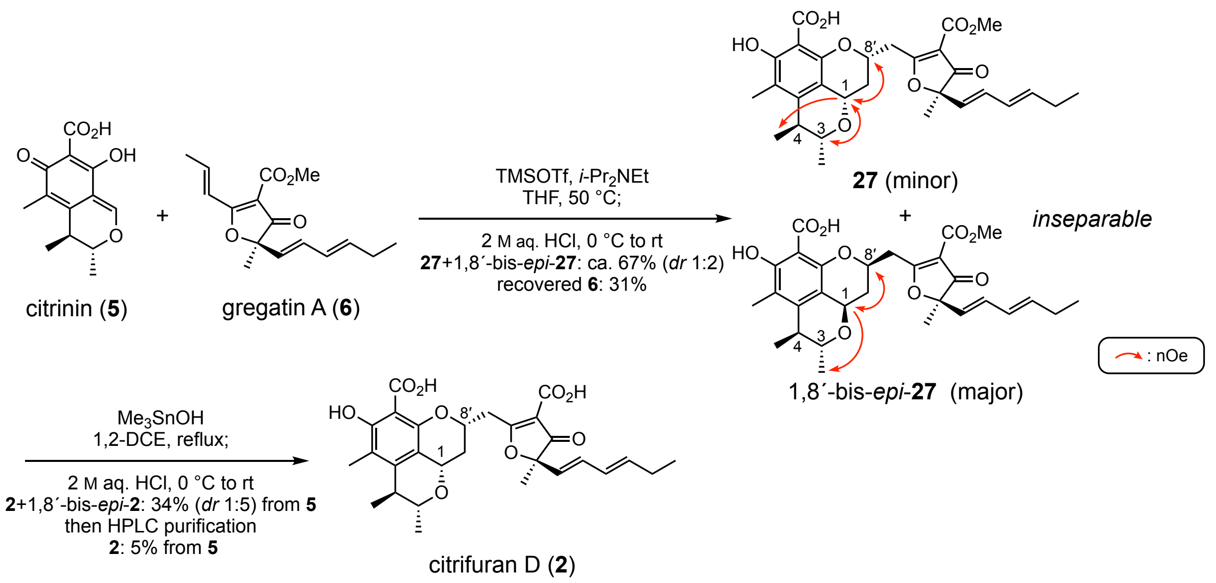

To a stirred solution of citrinin (**5**) (5.3 mg, 22 μmol) and gregatin A (**6**) (5.9 mg, 21 μmol) in THF (430 μL) were added diisopropylethylamine (19 μL, 110 μmol, 5.0 equiv) and trimethylsilyl trifluoromethanesulfonate (9.6 μL, 53 μmol, 2.5 equiv) at 0 °C. After 10 min of stirring at the same temperature, the mixture was heated using ChemiStation™ PPM-5512A at 50 °C for 12.5 h. The resulting mixture was cooled to 0 °C before the mixture was quenched with 2 M aq HCl (500 μL). The layers were separated and extracted with EtOAc. The combined organic layers were washed with brine, dried (Na_2SO_4), and concentrated in vacuo. The residue was purified by preparative TLC (Merck silica gel 60 RP-18 F254s plates, MeOH/H_2O/TFA = 88:11:0.3) to give 7.6 mg (ca. 67%) of an inseparable diastereomeric mixture containing **27** and 1,8-bis-*epi*-**27** as a major components in a ration of 1:2 (based on ^{1}H NMR measurement) as a pale orange solid together with 2.8 mg (31%) of recovered gregatin A (**6**).

To a stirred solution of the mixture of the diastereomers of **27** obtained above (7.6 mg, ca. 14 μmol) in 1,2-DCE (750 μL) was added Me_3SnOH (130 mg, 720 μmol, 50 equiv) at room temperature. The mixture was heated using ChemiStation™ PPM-5512A at 90 °C for 24 h. The resulting mixture was cooled to 0 °C before the mixture was quenched with 2 M aq HCl (750 μL). The layers were separated and extracted with EtOAc. The combined organic layers were washed with brine, dried (Na_2SO_4), and concentrated in vacuo. The residue was roughly purified by preparative TLC (Merck silica gel 60 RP-18 F254s plates, MeOH/H_2O = 7:1) to give 3.8 mg (34%, 2 steps from **5**) of a 1:5 diastereomeric mixture of citrifuran D (**2**) and 1,8´-bis-*epi*-citrifuran D (based on ^{1}H NMR measurement) as a pale red solid. From this mixture, citrifuran D (**2**) was isolated by sequential preparative HPLC (MeOH/H_2O/HCO_2H = 80:20:0.1) followed by preparative TLC (Merck silica gel 60 RP-18 F254s plates, MeOH/H_2O/TFA = 86:14:0.2), providing 0.6 mg (5% isolated yield; 2 steps from **5**) of citrifuran D (**2**) as a white amorphas solid.

Specific Rotation: $[\alpha]_D^{23}$ –60 (*c* 0.05, MeOH)

^{1}H NMR (600 MHz, $CDCl_3$, ppm): δ = 12.43 (s, 1H), 6.23 (dd, *J* = 15.6, 10.5 Hz, 1H), 5.95 (dd, *J* = 15.0, 10.5 Hz, 1H), 5.74 (dt, *J* = 15.0, 6.6 Hz, 1H), 5.52 (d, *J* = 15.6 Hz, 1H), 4.88 (m, 1H), 4.58 (dd, *J* = 11.4, 6.0 Hz, 1H), 3.83 (dd, *J* = 14.4, 3.0 Hz, 1H), 3.67 (m, 1H), 3.49 (dd, *J* = 14.4, 9.0 Hz, 1H), 2.84 (m, 1H), 2.53 (dd, *J* = 12.6, 6.0 Hz, 1H), 2.13 (s, 3H), 2.09 (m, 2H), 1.89 (seemingly q; interpreted as ddd, *J* = 12.6, 11.4 Hz, 1H), 1.67 (s, 3H), 1.36 (d, *J* = 6.0 Hz, 3H), 1.20 (d, *J* = 7.2 Hz, 3H), 0.98 (t, *J* = 7.2 Hz, 3H)

^{13}C NMR (150 MHz, $CDCl_3$, ppm): δ = 203.3, 193.8, 170.7, 163.4 (br), 161.7, 148.3, 145.9, 141.6, 134.1, 127.0, 122.5, 118.8, 111.6, 106.8 (br), 97.5, 94.6, 78.8, 74.7, 68.5, 38.0, 36.3, 33.5, 25.7, 22.1, 21.5, 19.5, 13.1, 11.1

IR (ATR): ν_{max} 3262 (br), 2926 (vs), 1687 (s), 1593 (m) cm^{-1}

HRMS (ESI) *m/z*: [M–H]$^-$ Calcd for $C_{28}H_{31}O_9$ 511.1974; Found 511.1976

methyl (*R*)-5-((1*E*,3*E*)-hexa-1,3-dien-1-yl)-2-((2*R*,3*R*,3a*S*,5*R*,6*S*)-8-hydroxy-2,5,6,7-tetramethyl-3,3a,5,6-tetrahydro-2*H*-pyrano[2,3,4-*de*]chromen-3-yl)-5-methyl-4-oxo-4,5-dihydrofuran-3-carboxylate (28)

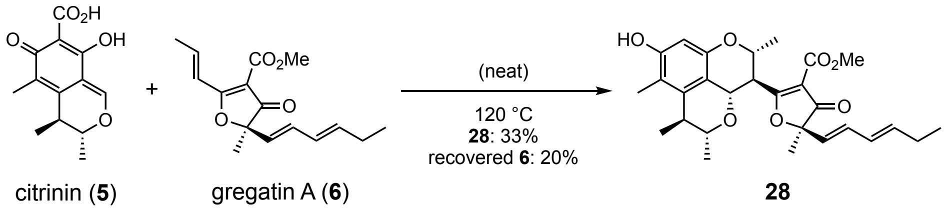

Citrinin (**5**) (12.4 mg, 49.5 μmol, 1.0 equiv) and gregatin A (**6**) (13.7 mg, 49.6 μmol, 1.0 equiv) were dissolved in EtOAc, and then concentrated in vacuo. The resulting residue was heated using oil bath at 120 °C for 4 h. After cooling to room temperature, the resulting dark brown oil was dissolved again in EtOAc and concentrated in vacuo, then heated again using oil bath at 120 °C for an additional 15 h. After cooling to room temperature, the residue was purified by preparative TLC (Merck silica gel 60 F254 plates, *n*-hexane/EtOAc = 2:1) to give 7.9 mg (33%) of cycloadduct **28** as a colorless wax together with 2.8 mg (20%) of recovered gregatin A (**6**).

Specific Rotation: $[\alpha]_D^{24}$ –38 (*c* 0.18, MeOH)

^{1}H NMR (600 MHz, $CDCl_3$, ppm): δ = 6.27 (dd, *J* = 15.6, 10.8 Hz, 1H), 6.21 (s, 1H), 5.99 (dd, *J* = 15.0, 10.8 Hz, 1H), 5.82 (dt, *J* = 15.0, 6.6 Hz, 1H), 5.55 (d, *J* = 15.6 Hz, 1H), 5.00 (brs, 1H, phenolic O*H*), 4.89 (d, *J* = 10.8 Hz, 1H), 4.45 (brs, 1H), 4.13 (brs, 1H), 3.80 (s, 3H), 3.56 (m, 1H), 2.78 (m, 1H), 2.11 (m, 2H), 2.10 (s, 3H), 1.57 (s, 3H), 1.39 (d, *J* = 6.0 Hz, 3H), 1.25 (d, *J* = 6.0 Hz, 3H), 1.19 (d, *J* = 7.2 Hz, 3H), 1.00 (t, *J* = 7.5 Hz, 3H)

^{13}C NMR (150 MHz, $CDCl_3$, ppm): δ = 198.3, 195.0, 162.8, 154.6, 150.6, 139.8, 138.9, 132.2, 127.6, 124.9, 114.6, 112.5, 99.8, 91.3, 79.0, 72.6, 72.2, 51.8, 46.5 (br), 37.7, 25.7, 22.6, 21.2, 19.5, 18.9, 13.2, 11.0

IR (ATR): ν_{max} 3403 (br), 2974 (m), 1712 (s), 1588 (s) cm^{-1}

HRMS (ESI) *m/z*: [M+Na]$^+$ Calcd for $C_{28}H_{34}NaO_7$ 505.2197; Found 505.2197

citrifuran A (1)

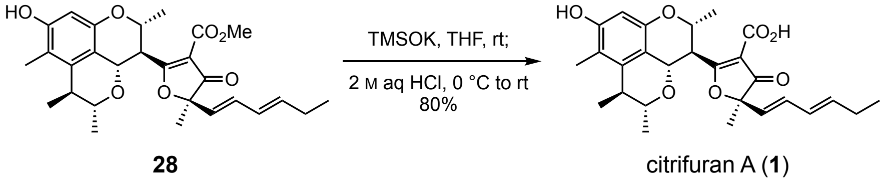

To a stirred solution of cycloadduct **28** (3.6 mg, 7.5 μmol) in THF (200 μL) was added TMSOK (2.9 mg, 23 μmol, 3.0 equiv) at room temperature. After 4 h of stirring at the same temperature, the mixture was added 2 M aq HCl (200 μL) at 0 °C, and then allowed to warm to room temperature. After 45 min of stirring at the same temperature, the layers were separated and extracted with EtOAc. The combined organic layers were washed with brine, dried (Na_2SO_4), and concentrated in vacuo. The residue was purified by preparative TLC (Merck silica gel 60 RP-18 F254s plates, MeOH/H_2O = 13:2) to give 2.8 mg (80%) of citrifuran A (**1**) as a pale yellow solid.

Mp: 92.5–94.3 °C (dec.)

Specific Rotation: $[\alpha]_D^{24}$ –35 (*c* 0.14, MeOH)

^{1}H NMR (600 MHz, $CDCl_3$, ppm): δ = 6.31 (dd, *J* = 15.6, 10.5 Hz, 1H), 6.20 (s, 1H), 6.01 (dd, *J* = 15.0, 10.5 Hz, 1H), 5.89 (dt, *J* = 15.0, 6.3 Hz, 1H), 5.53 (d, *J* = 15.6 Hz, 1H), 4.95 (brs, 2H), 4.43 (brs, 1H), 4.29 (brs, 1H), 3.60 (m, 1H), 2.78

(m, 1H), 2.13 (m, 2H), 2.10 (s, 3H), 1.65 (s, 3H), 1.37 (d, J = 6.0 Hz, 3H), 1.24 (d, J = 6.6 Hz, 3H), 1.19 (d, J = 7.2 Hz, 3H), 1.01 (t, J = 7.5 Hz, 3H)

^{1}H NMR (600 MHz, 30 °C DMSO-d_6, ppm): δ = 9.31 (s, 1H), 6.30 (dd, J = 15.3, 10.2 Hz, 1H), 6.18 (s, 1H), 6.08 (dd, J = 15.0, 10.2 Hz, 1H), 5.91 (dt, J = 15.0, 6.3 Hz, 1H), 5.61 (d, J = 15.3 Hz, 1H), 4.86 (d, J = 10.8 Hz, 1H), 4.47 (brdq, J = 9.3, 6.0 Hz, 1H), 3.95 (brs, 1H), 3.53 (m, 1H), 2.71 (m, 1H), 2.08 (m, 2H), 1.98 (s, 3H), 1.46 (s, 3H), 1.28 (d, J = 6.0 Hz, 3H), 1.19 (d, J = 6.0 Hz, 3H), 1.13 (d, J = 6.6 Hz, 3H), 0.96 (t, J = 7.5 Hz, 3H)

^{13}C NMR (150 MHz, 30 °C, DMSO-d_6, ppm): δ = 197.6, 193.8, 163.1, 156.0, 149.8, 139.1, 138.3, 131.9, 127.7, 125.5, 114.6, 110.9, 109.9 (br), 98.9, 90.4, 78.4, 71.9, 71.5, 46.1 (br), 37.2, 25.0, 21.6, 21.1, 19.2, 18.5, 13.1, 11.0

IR (ATR): ν_{max} 3384 (br), 2975 (m), 1734 (m), 1589 (s) cm^{-1}

HRMS (ESI) *m*/*z*: $[M+Na]^+$ Calcd for $C_{27}H_{32}NaO_7$ 491.2040; Found 491.2039

3. *Determination of the Enantiomeric Excess of* **10**

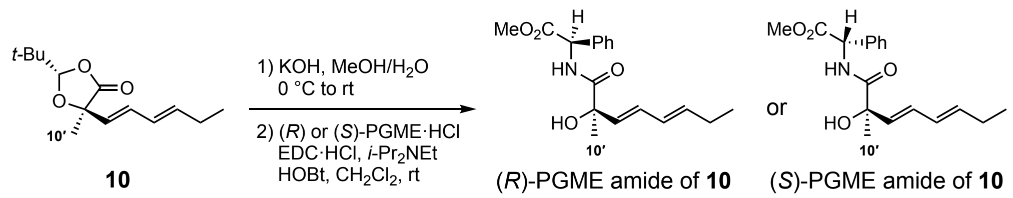

After hydrolysis of **10**, the resulting α-hydroxy carboxylic acid was converted into the corresponding (*R*)- and (*S*)- PGME amides by condensation with (*R*)- and (*S*)-PGME·HCl, respectively, in CH_2Cl_2 in the presence of EDC·HCl, *i*-Pr_2NEt and HOBt. By comparing the peak areas of the ^{1}H NMR signals for 10′-CH_3 (1.55/1.54 and 1.50 ppm), the enantiomeric excess (*ee*) of **10** was evaluated to be 96%. For the ^{1}H NMR spectra of (*R*)- and (*S*)- PGME amides of **10**, see p.p. S74–S77.

4. *Influence of the diastereomeric ratio of sulfoxide* **21** *on the total yield of* **22** *and* **22′**

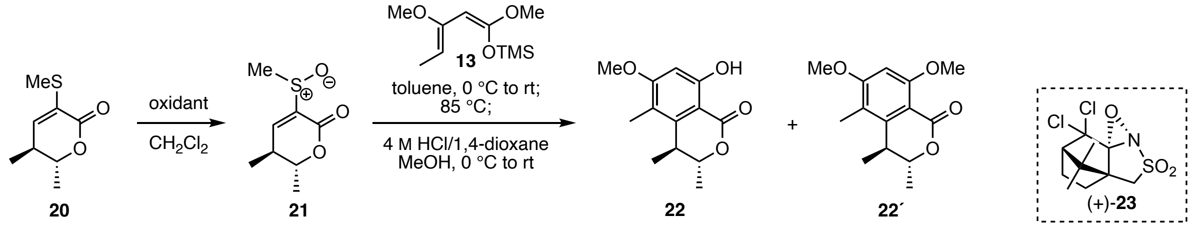

Table S1

entry	Oxidation of sulfide 20#				Diels–Alder-initiated aromatization reaction
	oxidant	temperatures	yield of **21** [%]	*dr* of **21**	total yield of **22** & **22′** [%]
1	*m*-CPBA	0 °C to rt	93	1.4:1	45
2	(+)-**23**	0 °C to rt	86	3.7:1	68
3	(+)-**23**	–40 °C to rt	96	11:1	82

#All reactions were performed in CH_2Cl_2.

We initially subjected sulfoxide **21**, which was obtained as a 1.4:1 epimeric mixture at the sulfur atom via the *m*-CPBA oxidation of **20**, to the Diels–Alder-initiated aromatization. However, the total yield of **22** and **22′** was moderate (45%, Table S1). After some unsuccessful experiments, we found that one epimer of **21** was recovered unchanged after the reaction although its stereochemistry was undetermined. This result would suggest that one of the two epimers of **21** preferentially underwent the Diels–Alder reaction with diene **13**. In fact, the yield of the aromatization reaction increased to 68% when a 3.7:1 epimeric mixture of **21**, which was obtained by oxidation of **20** with chiral oxaziridine (+)-**23** at 0 °C, was used for the Diels–Alder reaction. This transformation was eventually achieved in 82% total yield of **22** and **22′** using a 11:1 epimeric mixture of **21** prepared upon exposure of **20** to (+)-**23** in CH_2Cl_2 at –40 °C.

*5. Attempted conversion of lactol **24** into decarboxycitrinin (**3**) or citrifuran A (**1**)*

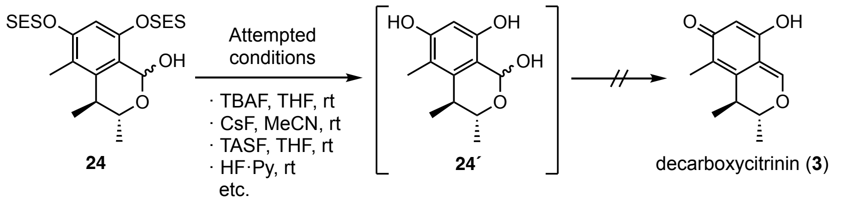

We attempted conversion of **24** into decarboxycitrinin (**3**). However, subjection of **24** to various deprotection conditions such as TBAF/THF, CsF/MeCN, TASF/THF, and HF·Py resulted in the formation of a complex mixture. Monitoring the reaction by measuring ^{1}H NMR spectra suggested that deprotection of the two 2-(trimethylsilyl)ethanesulfonyl (SES) groups likely proceeded but the reaction became messy during the subsequent dehydration of the resulting intermediate **24´** to **3**.

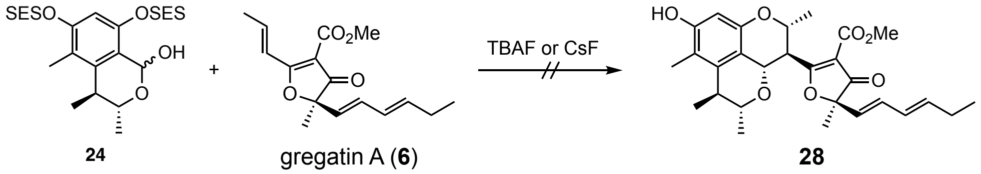

Attempts to obtain cycloadduct **28** by treating a mixture of **24** and gregatin A (**6**) with TBAF or CsF were also unsuccessful, resulting in the decomposition both substrates.

6. *Comparison of the ^1H and ^{13}C NMR spectral data of synthetic **2** with those reported for natural citrifuran D*

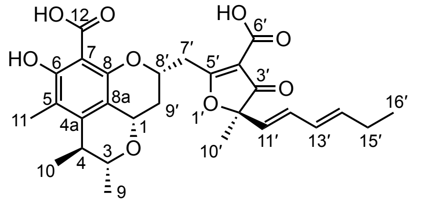

position	δ_H (mult, *J* in Hz)		δ_C	
	**Synthetic** (600 MHz)	**Isolated** (500 MHz)[a]	**Synthetic** (150 MHz)	**Isolated** (125 MHz)[a]
1	4.58 (dd, 11.4, 6.0)	4.56 (dd, 12.7, 5.9)	68.5	68.6
3	3.67 (m)	3.67 (m)	78.8	78.9
4	2.84 (m)	2.83 (m)	38.0	38.1
4a	-	-	145.9	146.0
5	-	-	118.8	118.8
6	-	-	161.7	161.9
7	-	-	97.5	97.8
8	-	-	148.3	148.5
8a	-	-	111.8	111.8
9	1.36 (d, 6.0)	1.34 (d, 6.1)	21.5	21.7
10	1.20 (d, 7.2)	1.19 (d, 7.0)	19.5	19.6
11	2.13 (s)	2.12 (s)	11.1	11.2
12	-	-	170.7	170.9
2′	-	-	94.6	94.4
3′	-	-	203.3	202.8
4′	-	-	106.8	107.3
5′	-	-	193.8	193.8
6′	-	-	163.4	163.6
7′	3.49 (dd, 14.4, 9.0)	3.50 (dd, 14.3, 8.5)	36.3	36.4
	3.83 (dd, 14.4, 3.0)	3.78 (brd, 14.3)		
8′	4.8 (m)	4.87 (m)	74.7	74.9
9′	1.89 (ddd, 12.6, 11.4)	1.87 (q, 12.7)	33.5	33.7
	2.53 (dd, 12.6, 6.0)	2.50 (dd, 12.7, 5.9)		
10′	1.67 (s)	1.65 (s)	22.1	22.2
11′	5.52 (d, 15.6)	5.51 (d, 15.5)	122.5	123.0
12′	6.23 (dd, 15.6, 10.3)	6.25 (dd, 15.5, 10.3)	134.1	134.0
13′	5.95 (dd, 15.0, 10.5)	5.93 (dd, 15.5, 10.3)	127.0	127.2
14′	5.74 (dt, 15.0, 6.6)	5.71 (dt, 15.5, 6.6)	141.6	141.4
15′	2.09 (m)	2.07 (m)	25.7	25.8
16′	0.98 (t, 7.2)	0.96 (t, 7.5)	13.1	13.2
6-OH	12.43 (s)			

[a] Chemical shifts reported by Kong *et al.* for natural citrifuran D.[1)]

7. *Comparison of the ^{1}H and ^{13}C NMR spectral data of synthetic 1 with those reported for natural citrifuran A*

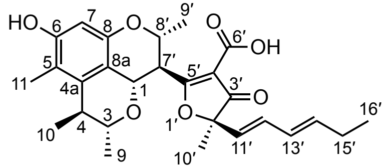

position	δ_H (mult, *J* in Hz)[a]		δ_H (mult, *J* in Hz)[b]		δ_C[b]	
	**Synthetic** (600 MHz)	**Isolated** (600 MHz)[c]	**Synthetic** (600 MHz)	**Isolated** (500 MHz)[c]	**Synthetic** (150 MHz)	**Isolated** (125 MHz)[c]
1	4.95 (brs)	4.98 (brs)	4.86 (d, 10.8)	4.86 (d, 10.7)	71.5	71.6
3	3.60 (m)	3.60 (m)	3.53 (m)	3.53 (m)	78.4	78.5
4	2.78 (m)	2.78 (m)	2.71 (m)	2.70 (m)	37.2	37.2
4a	-	-	-	-	138.3	138.3
5	-	-	-	-	114.6	114.6
6	-	-	-	-	156.0	156.1
7	6.20 (s)	6.21 (s)	6.18 (s)	6.18 (s)	98.9	98.9
8	-	-	-	-	149.8	149.8
8a	-	-	-	-	110.9	111.0
9	1.24 (d, 6.6)	1.23 (d, 6.2)	1.19 (d, 6.0)	1.19 (d, 6.8)	21.1	21.2
10	1.19 (d, 7.2)	1.19 (d, 6.9)	1.13 (d, 6.6)	1.12 (d, 6.8)	19.2	19.2
11	2.10 (s)	2.09 (s)	1.98 (s)	1.98 (s)	11.0	11.1
2′	-	-	-	-	90.4	90.6
3′	-	-	-	-	197.6	197.6
4′	-	-	-	-	109.9	109.8
5′	-	-	-	-	193.8	194.0
6′	-	-	-	-	163.1	163.2
7′	4.29 (brs)	4.27 (brs)	3.95 (brs)	3.95 (brs)	46.1	46.1
8′	4.43 (brs)	4.45 (brs)	4.47 (brdq, 9.3, 6.0)	4.46 (dq, 12.6, 6.2)	71.9	71.9
9′	1.37 (d, 6.0)	1.37 (d, 6.1)	1.28 (d, 6.0)	1.28 (d, 6.2)	18.5	18.5
10′	1.65 (s)	1.65 (s)	1.46 (s)	1.46 (s)	21.6	21.6
11′	5.53 (d, 15.6)	5.53 (d, 15.5)	5.61 (d, 15.3)	5.62 (d, 15.4)	125.5	125.5
12′	6.31 (dd, 15.6, 10.5)	6.32 (dd, 15.5, 10.3)	6.30 (dd, 15.3, 10.2)	6.30 (dd, 15.4, 10.4)	131.9	132.0
13′	6.01 (dd, 15.0, 10.5)	6.01 (dd, 15.5, 10.3)	6.08 (dd, 15.0, 10.2)	6.08 (dd, 15.4, 10.4)	127.7	127.8
14′	5.89 (dt, 15.0, 6.3)	5.87 (dt, 15.5, 6.5)	5.91 (dt, 15.0, 6.3)	5.91 (dt, 15.4, 6.5)	139.1	139.2
15′	2.13 (m)	2.13 (m)	2.08 (m)	2.08 (m)	25.0	25.1
16′	1.01 (t, 7.5)	1.01 (t, 7.4)	0.96 (t, 7.5)	0.96 (t, 7.4)	13.1	13.2
6-OH			9.31 (s)	9.35 (s)		

[a] Chemical shifts of citrifuran A in $CDCl_3$, [b] Chemical shifts of citrifuran A in DMSO-d_6, [c] Chemical shifts reported by Kong *et al.* for natural citrifuran A.[1)]

8. NMR Spectra

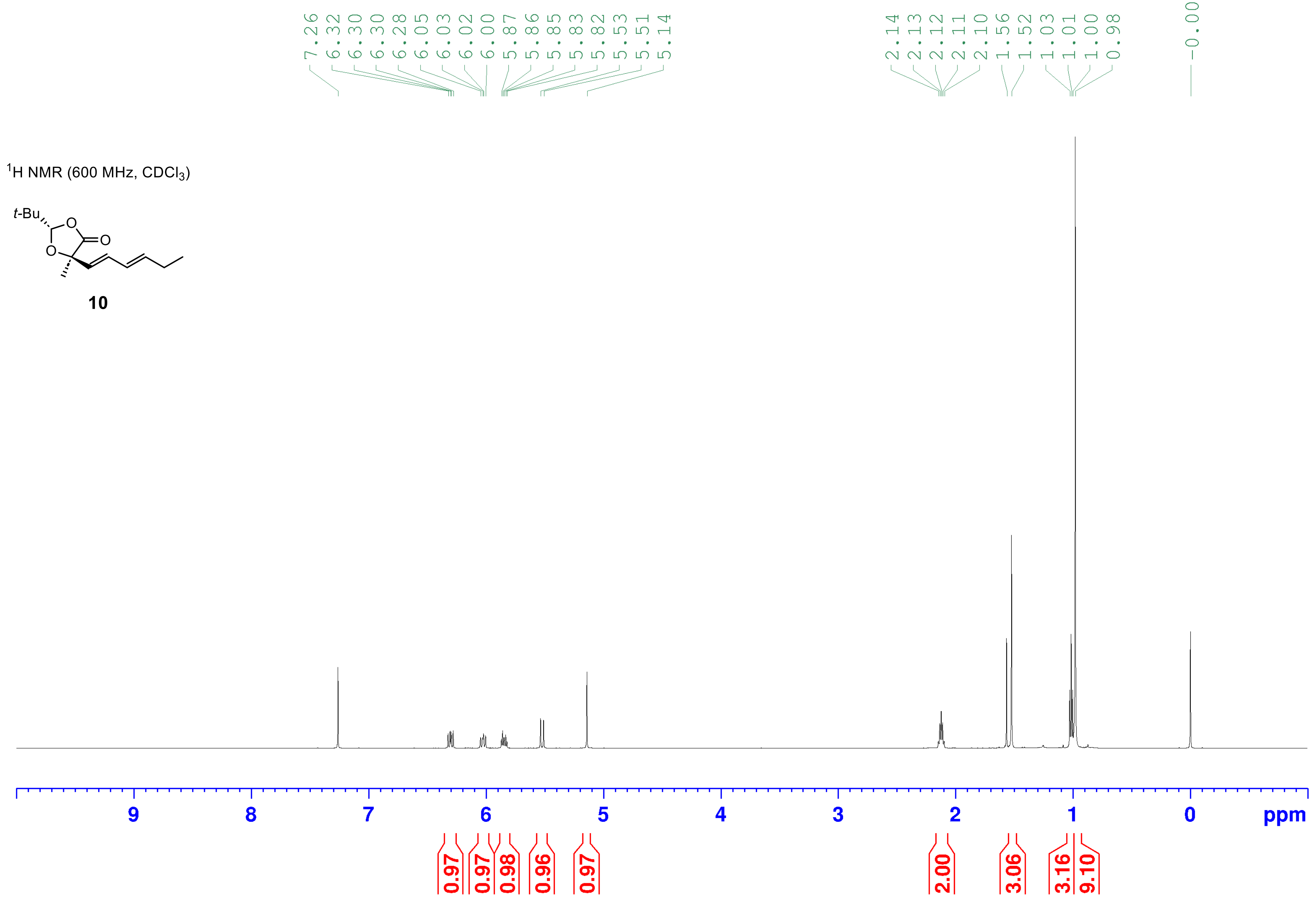

^{13}C NMR (150 MHz, $CDCl_3$)

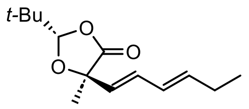

10

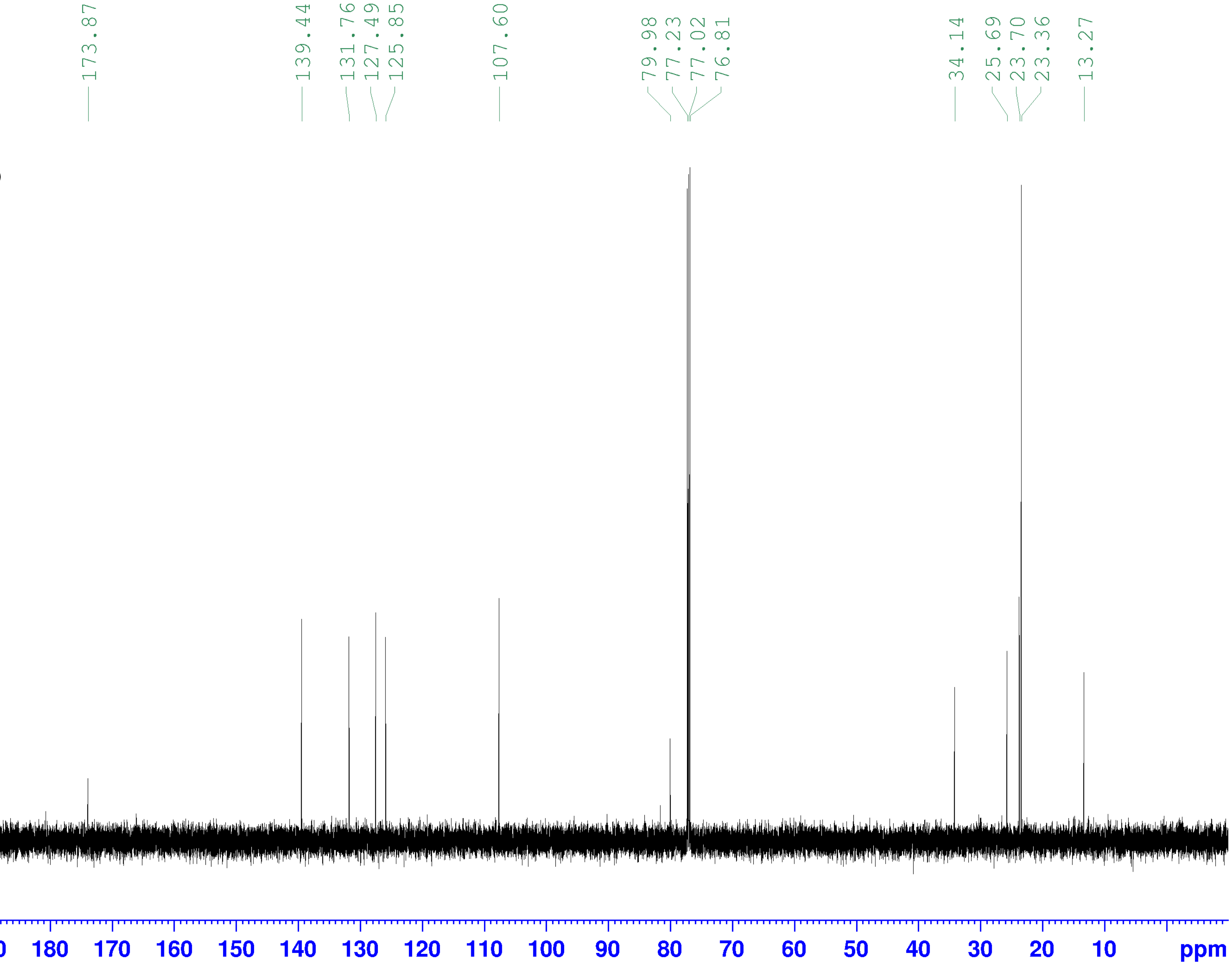

^{1}H NMR (600 MHz, $CDCl_3$)

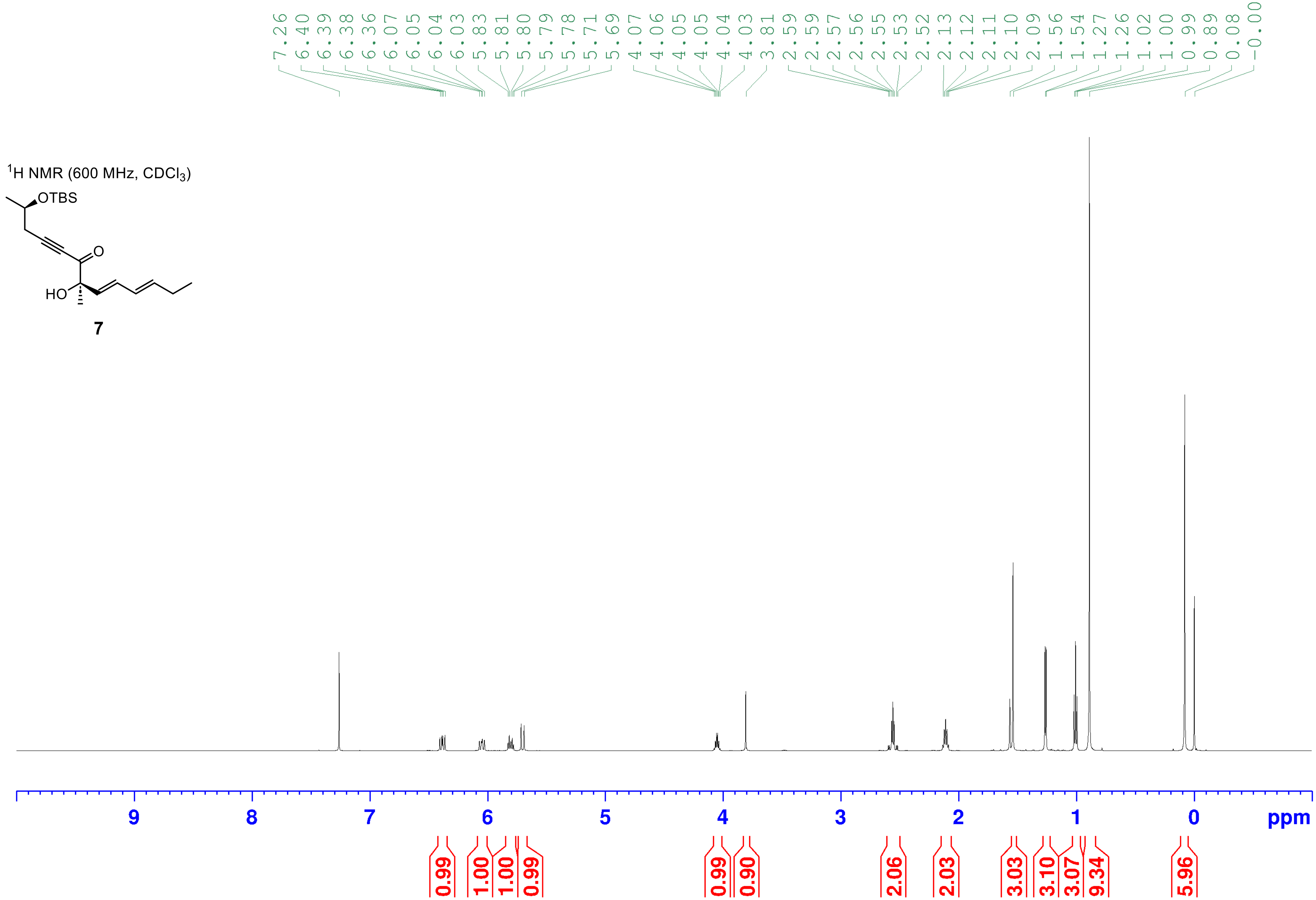

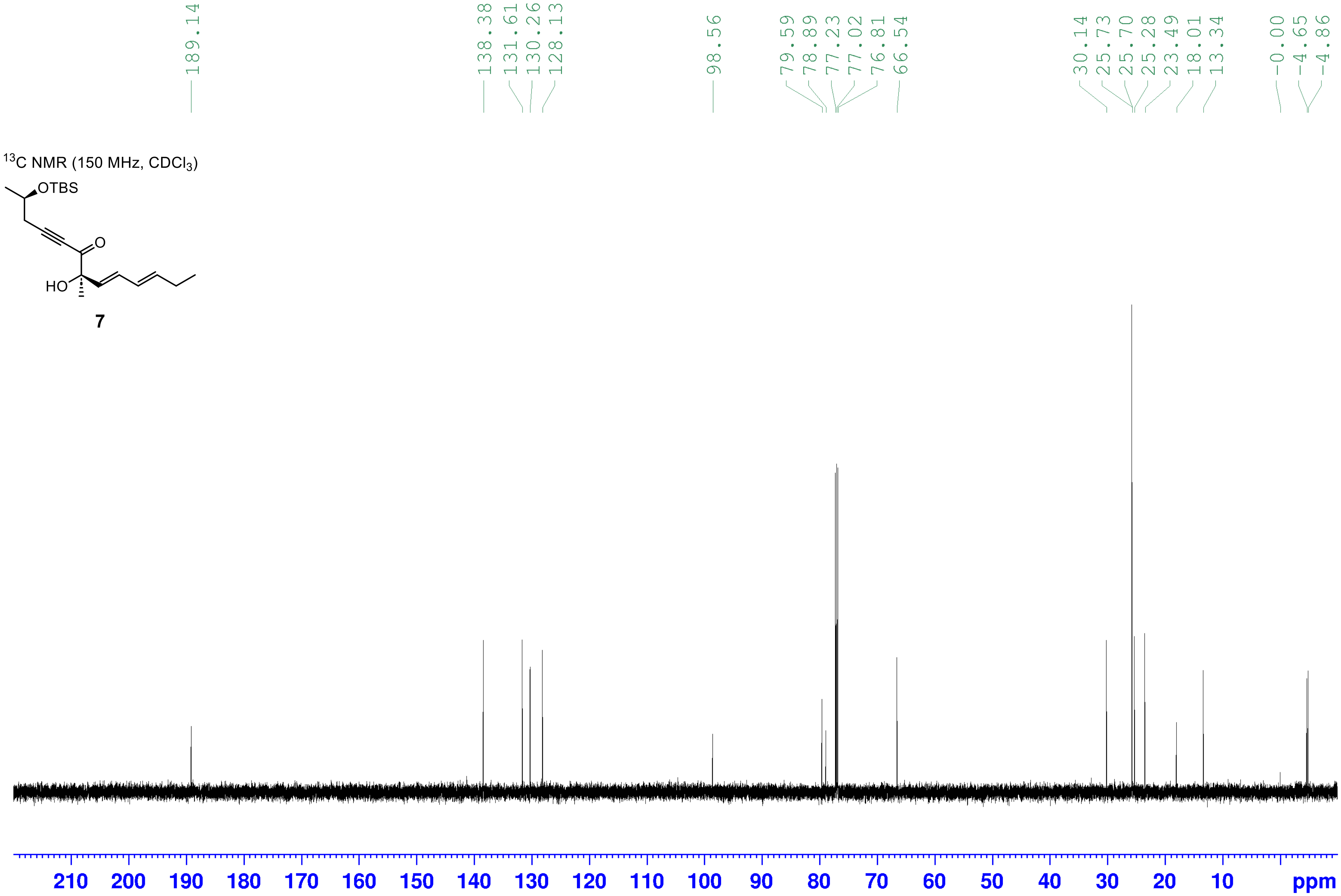
189.14
138.38
131.61
130.26
128.13
98.56
79.59
78.89
77.23
77.02
76.81
66.54
30.14
25.73
25.70
25.28
23.49
18.01
13.34
-0.00
-4.65
-4.86
^{13}C NMR (150 MHz, CDCl$_3$)
OTBS
O
HO
7
210 200 190 180 170 160 150 140 130 120 110 100 90 80 70 60 50 40 30 20 10 ppm

^{1}H NMR (600 MHz, $CDCl_3$)

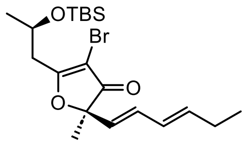

17

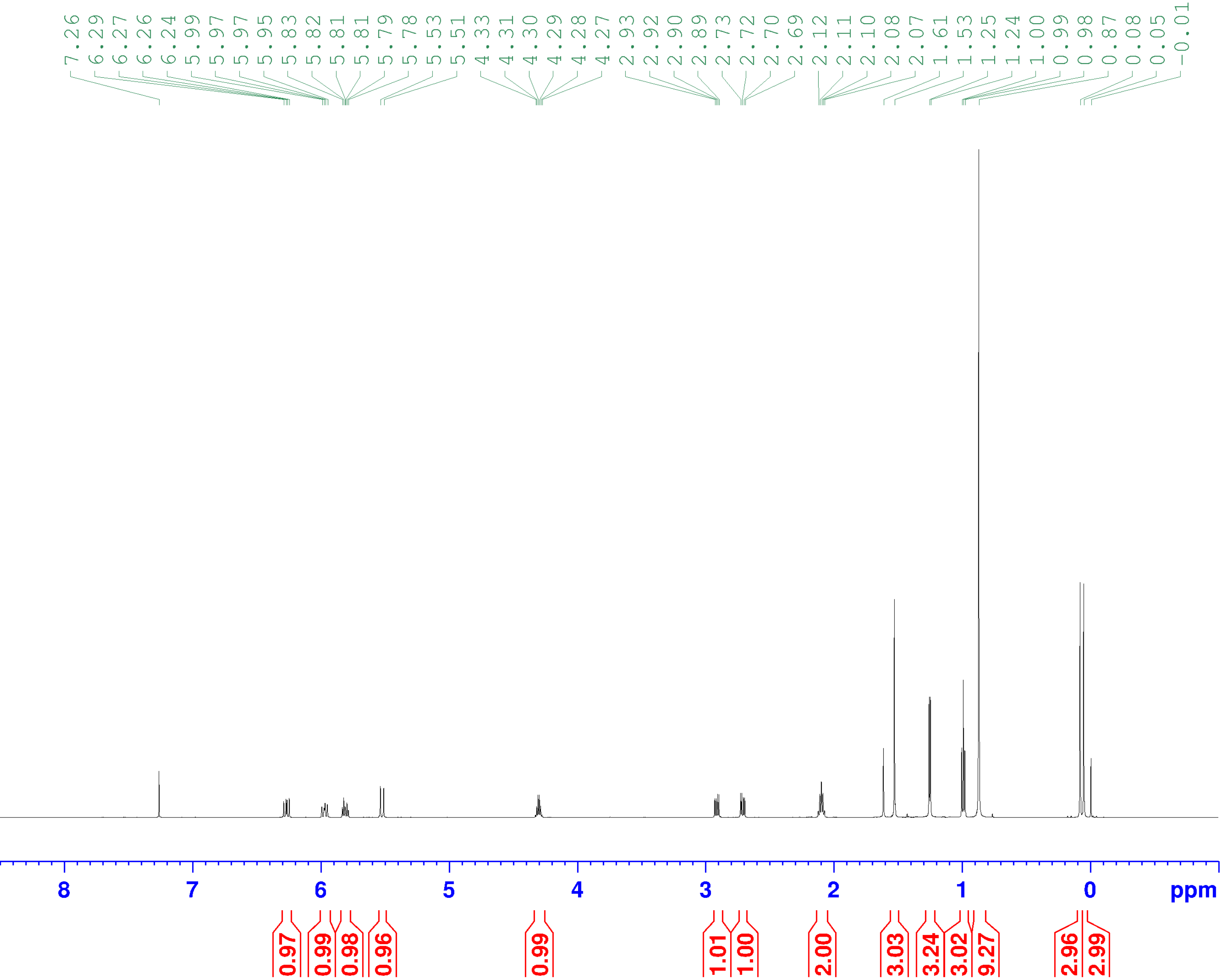

^{13}C NMR (150 MHz, $CDCl_3$)

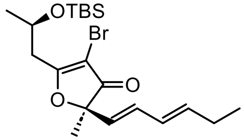

17

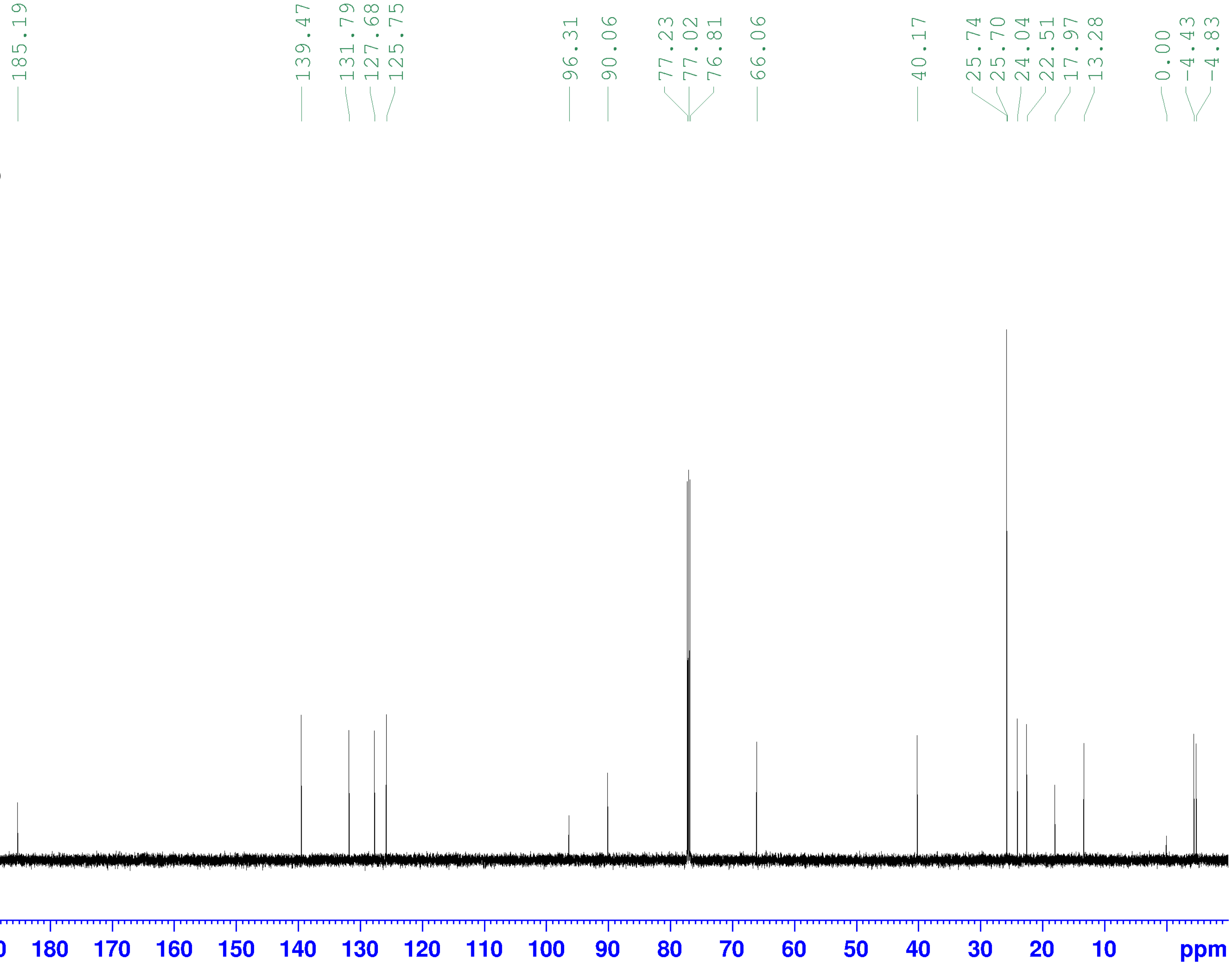

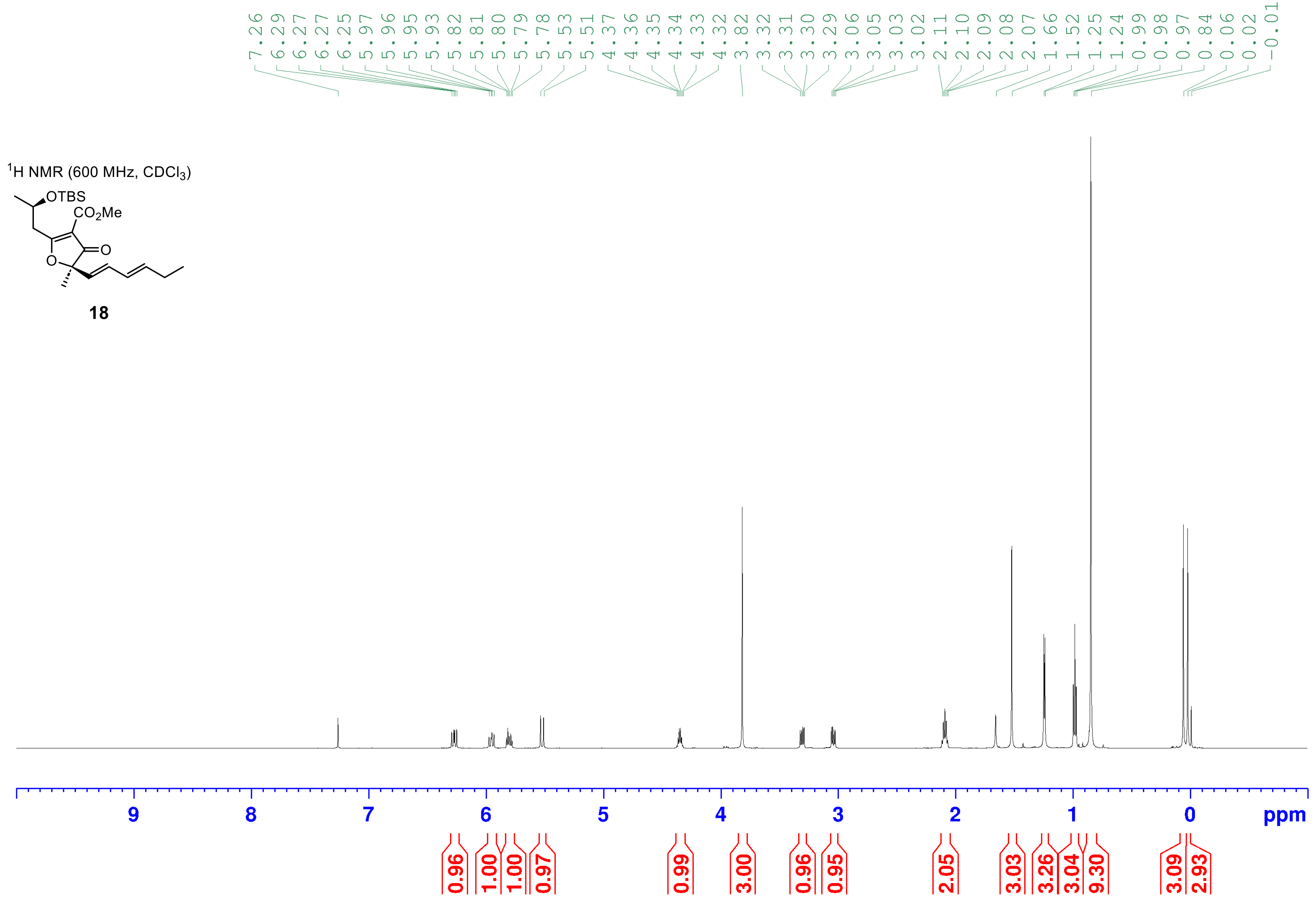

1H NMR (600 MHz, CDCl3)
OTBS
CO2Me
O
O
18
7.26
6.29
6.27
6.27
6.25
5.97
5.96
5.95
5.93
5.82
5.81
5.80
5.79
5.78
5.53
5.51
4.37
4.36
4.35
4.34
4.33
4.32
3.82
3.32
3.31
3.30
3.29
3.06
3.05
3.03
3.02
2.11
2.10
2.09
2.08
2.07
1.66
1.52
1.25
1.24
0.99
0.98
0.97
0.84
0.06
0.02
-0.01
0.96
1.00
1.00
0.97
0.99
3.00
0.96
0.95
2.05
3.03
3.26
3.04
9.30
3.09
2.93
9
8
7
6
5
4
3
2
1
0
ppm

^{13}C NMR (150 MHz, $CDCl_3$)

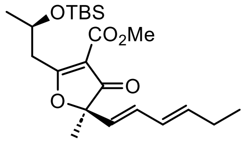

18

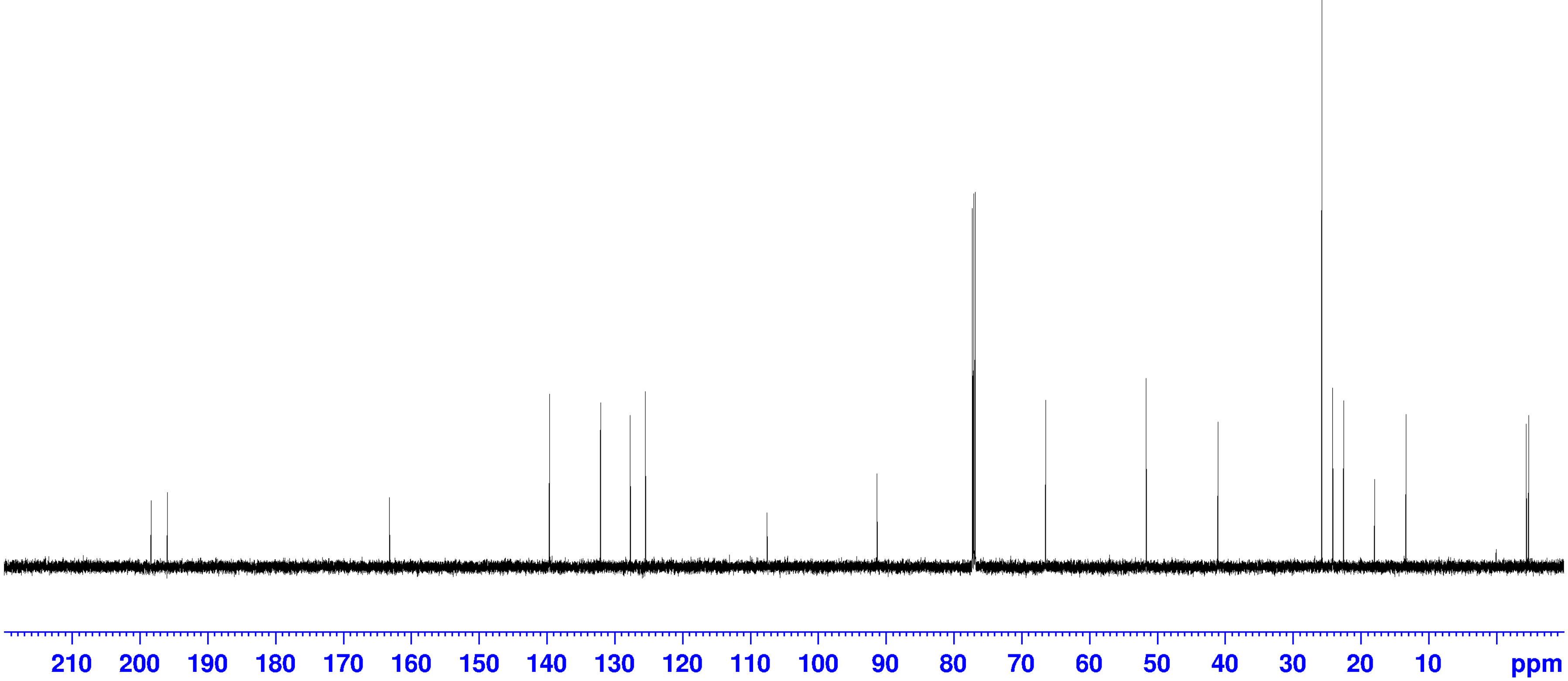

^{1}H NMR (600 MHz, benzene-d_6)

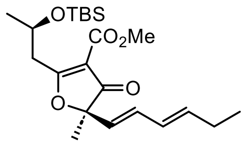

18

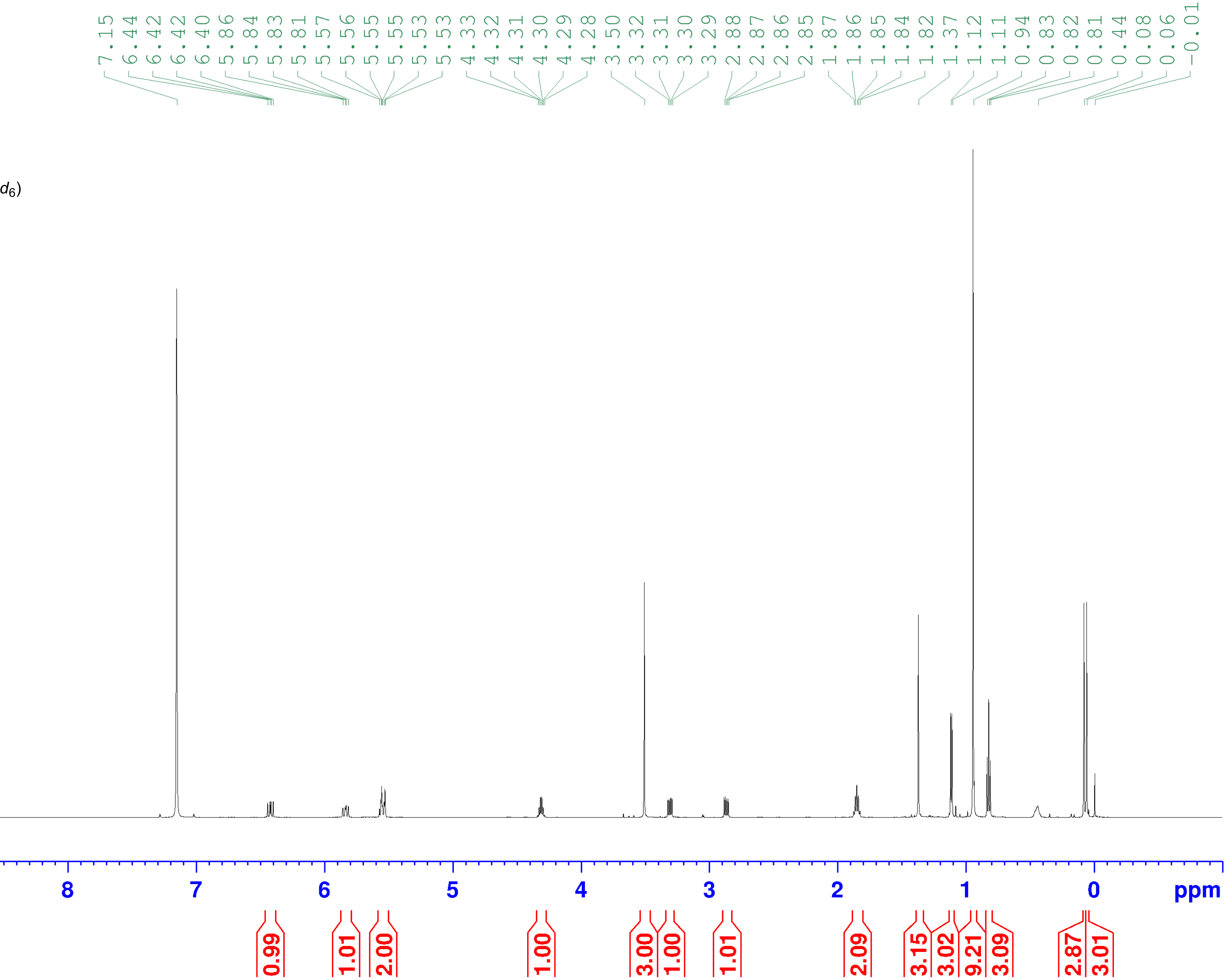

^{13}C NMR (150 MHz, benzene-d_6)

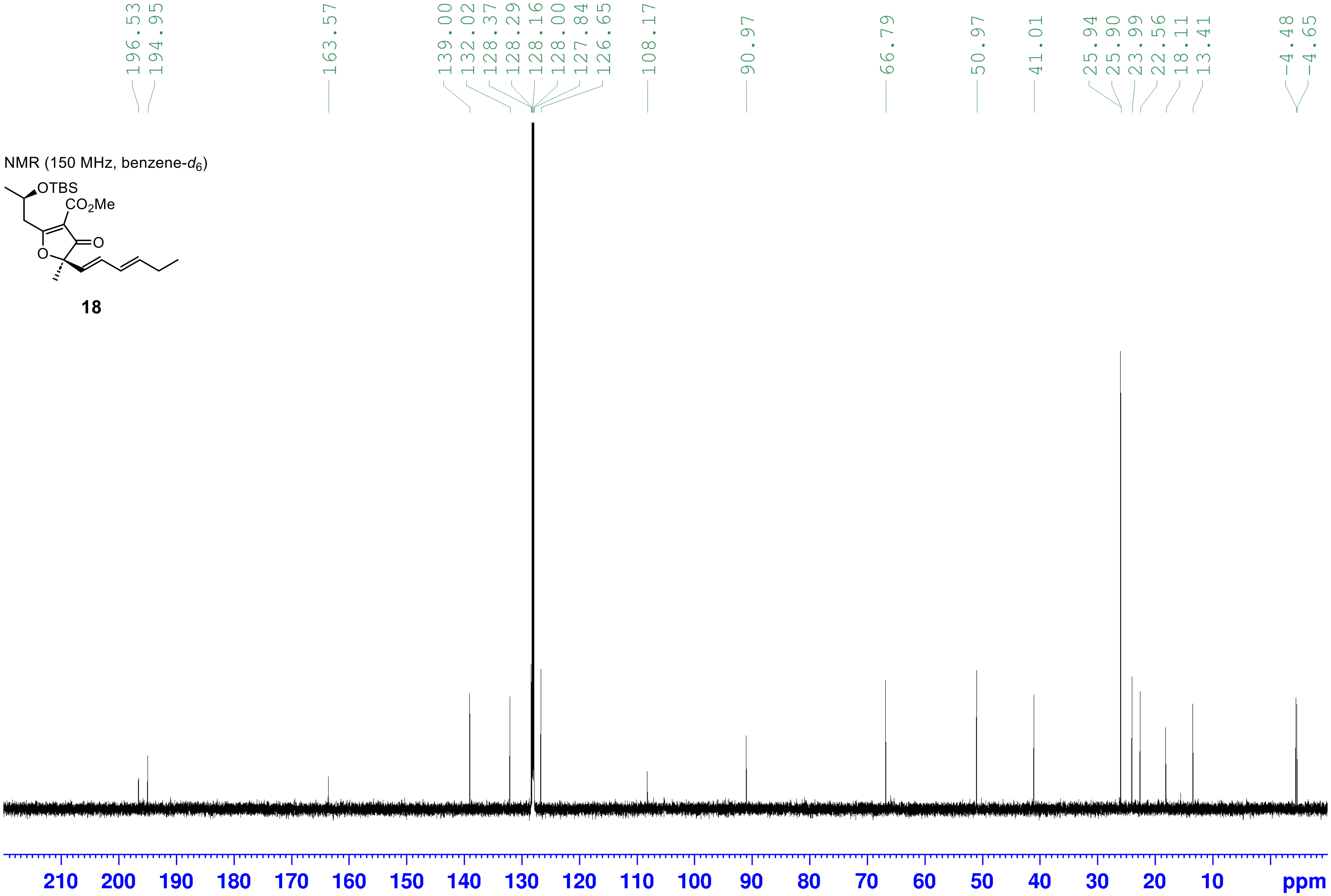

^{1}H NMR (600 MHz, $CDCl_3$)

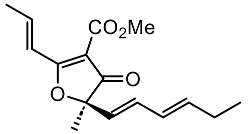

gregatin A (**6**)

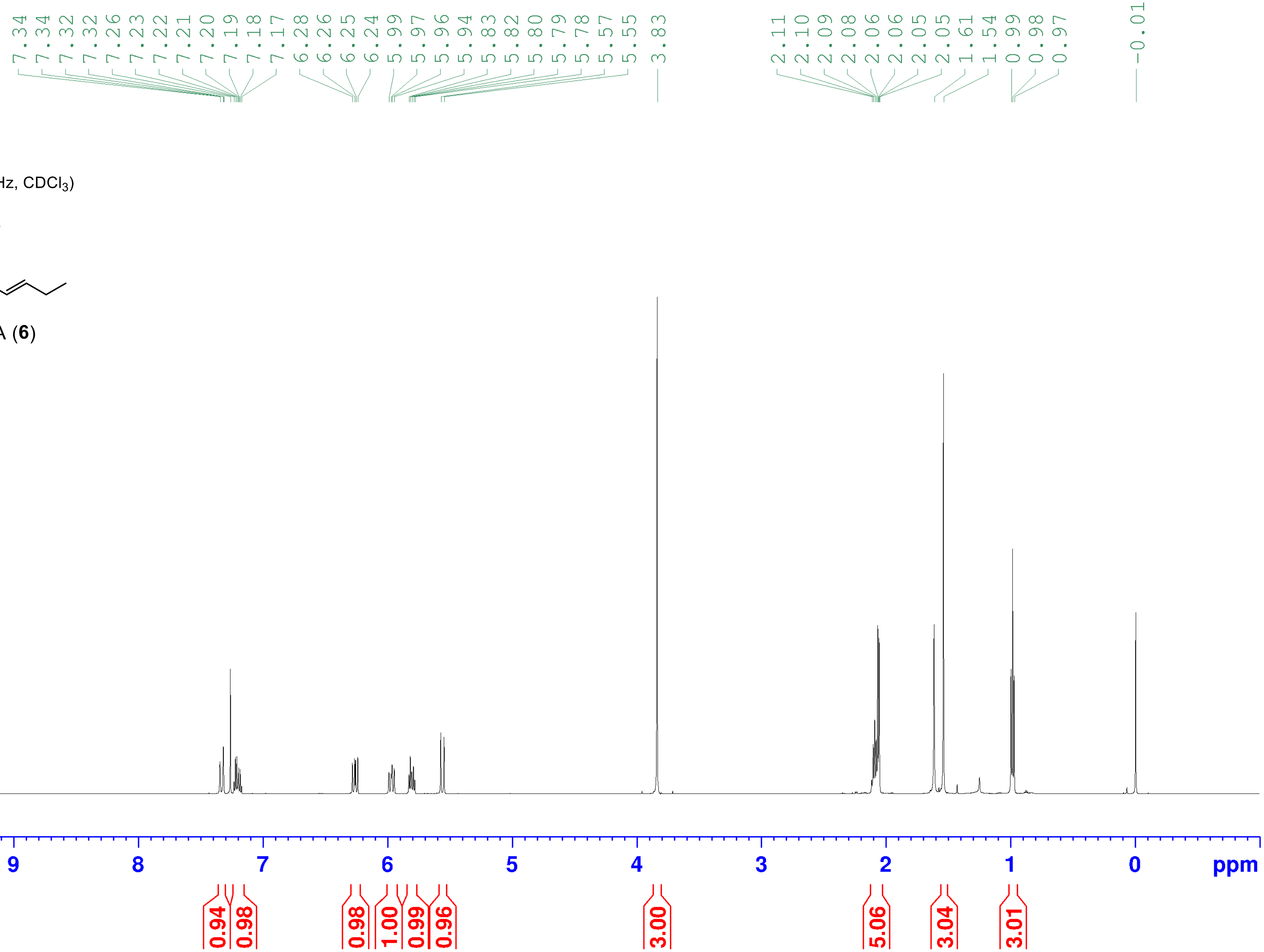

^{13}C NMR (150 MHz, $CDCl_3$)

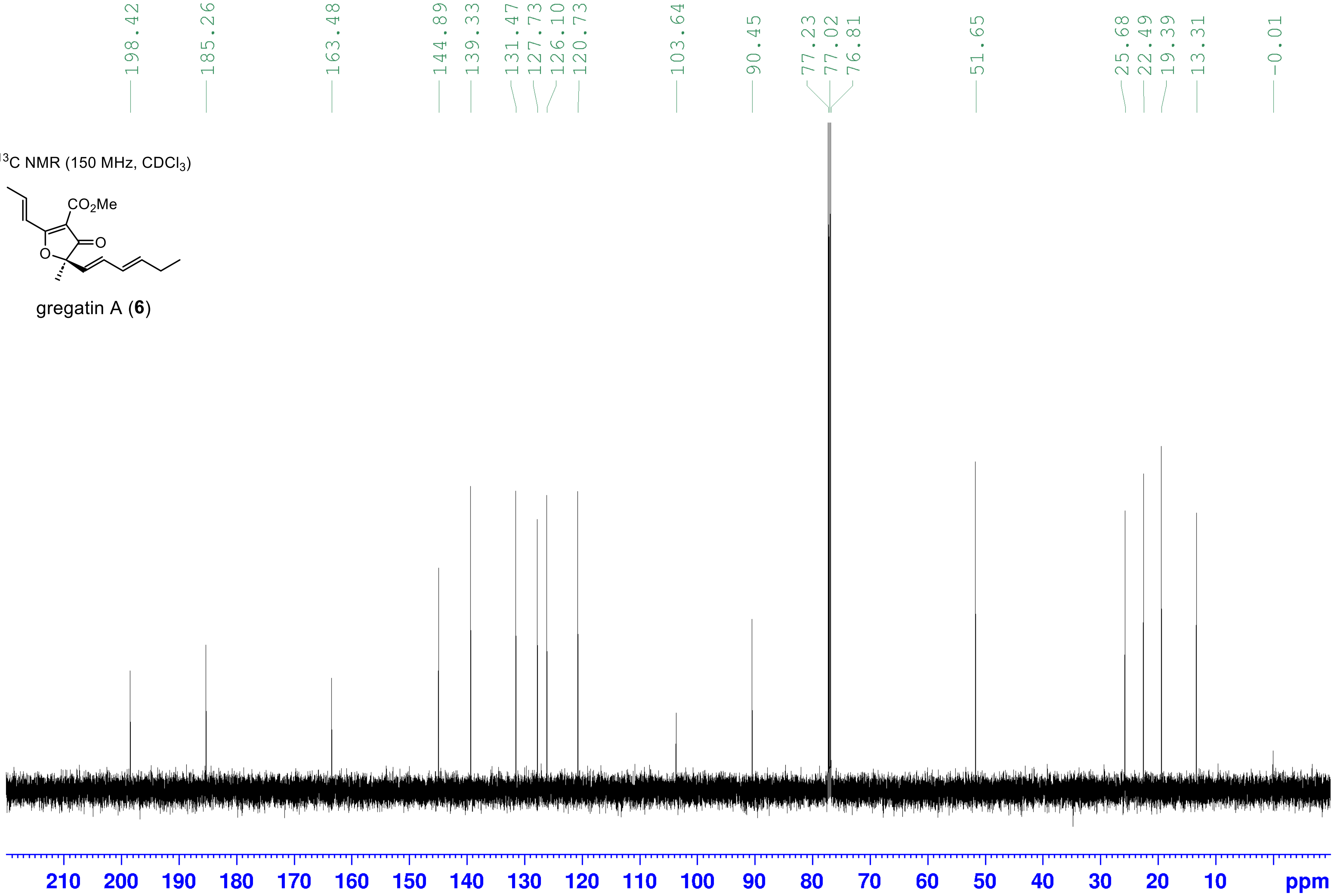

gregatin A (**6**)

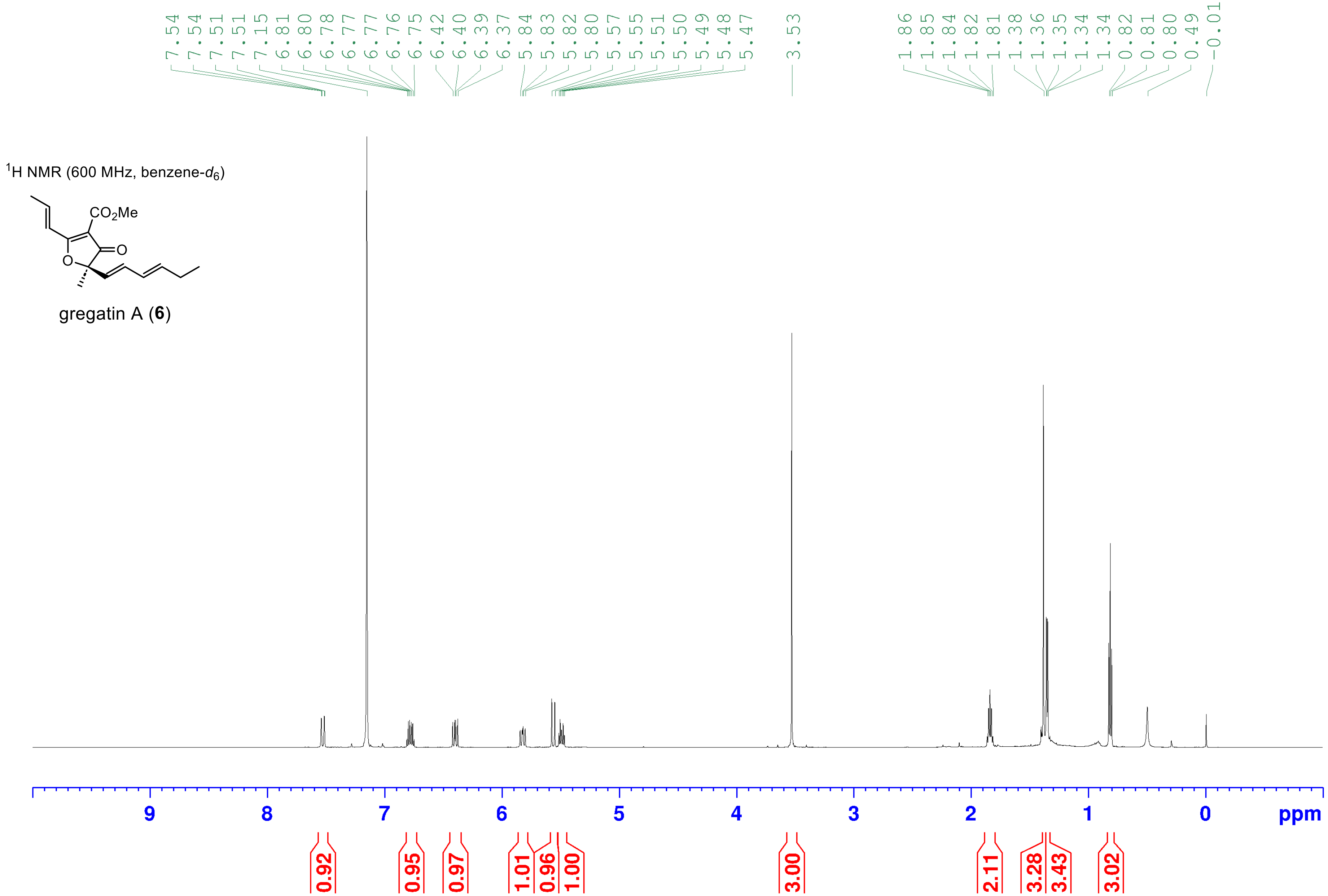
^{1}H NMR (600 MHz, benzene-d_6)
CO_2Me
O
gregatin A (**6**)
7.54 7.54 7.51 7.51 7.15 6.81 6.80 6.78 6.77 6.77 6.76 6.75 6.42 6.40 6.39 6.37 5.84 5.83 5.82 5.80 5.57 5.55 5.51 5.50 5.49 5.48 5.47
3.53
1.86 1.85 1.84 1.82 1.81 1.38 1.36 1.35 1.34 1.34 0.82 0.81 0.80 0.49 -0.01
9 8 7 6 5 4 3 2 1 0 ppm
0.92 0.95 0.97 1.01 0.96 1.00 3.00 2.11 3.28 3.43 3.02

^{13}C NMR (150 MHz, benzene-d_6)

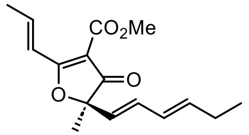

gregatin A (**6**)

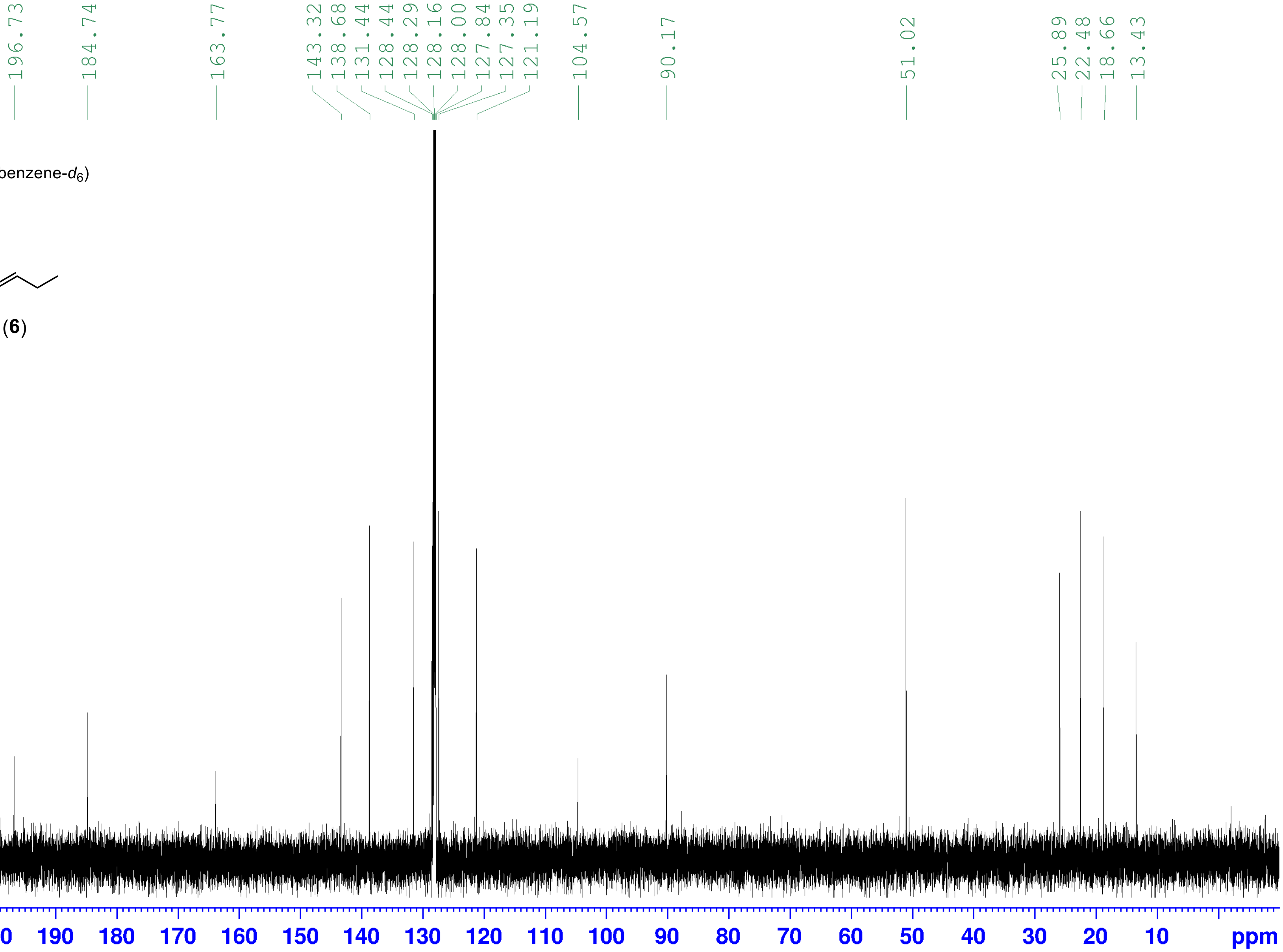

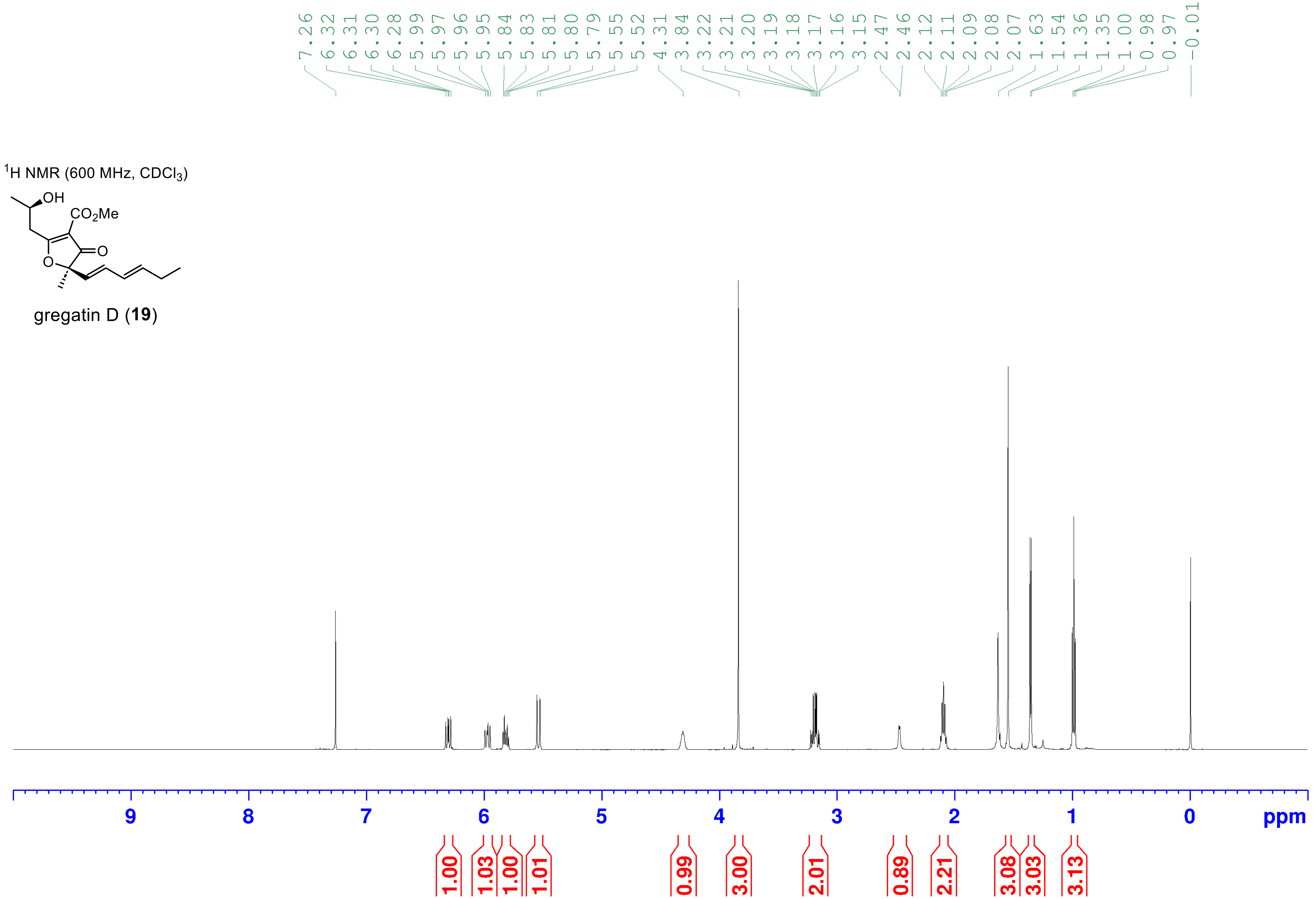
1H NMR (600 MHz, CDCl3)
OH
CO2Me
O
O
gregatin D (19)
7.26
6.32
6.31
6.30
6.28
5.99
5.97
5.96
5.95
5.84
5.83
5.81
5.80
5.79
5.55
5.52
4.31
3.84
3.22
3.21
3.20
3.19
3.18
3.17
3.16
3.15
2.47
2.46
2.12
2.11
2.09
2.08
2.07
1.63
1.54
1.36
1.35
1.00
0.98
0.97
-0.01
9
8
7
6
5
4
3
2
1
0
ppm
1.00
1.03
1.00
1.01
0.99
3.00
2.01
0.89
2.21
3.08
3.03
3.13

^{13}C NMR (150 MHz, $CDCl_3$)

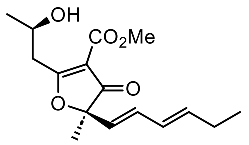

gregatin D (**19**)

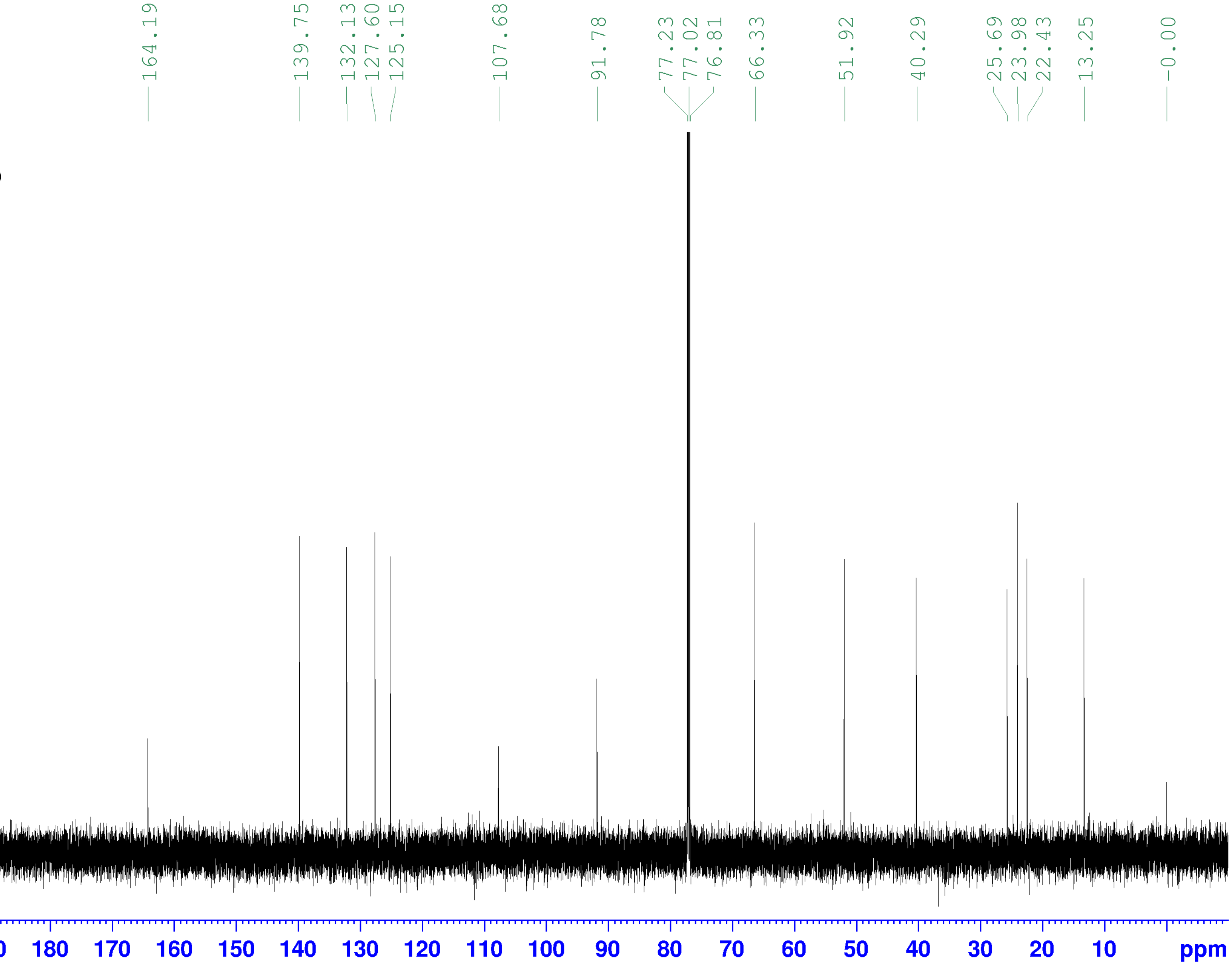

^1H NMR (600 MHz, $CDCl_3$)

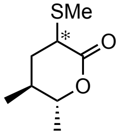

SI-4a (major diasteromer)

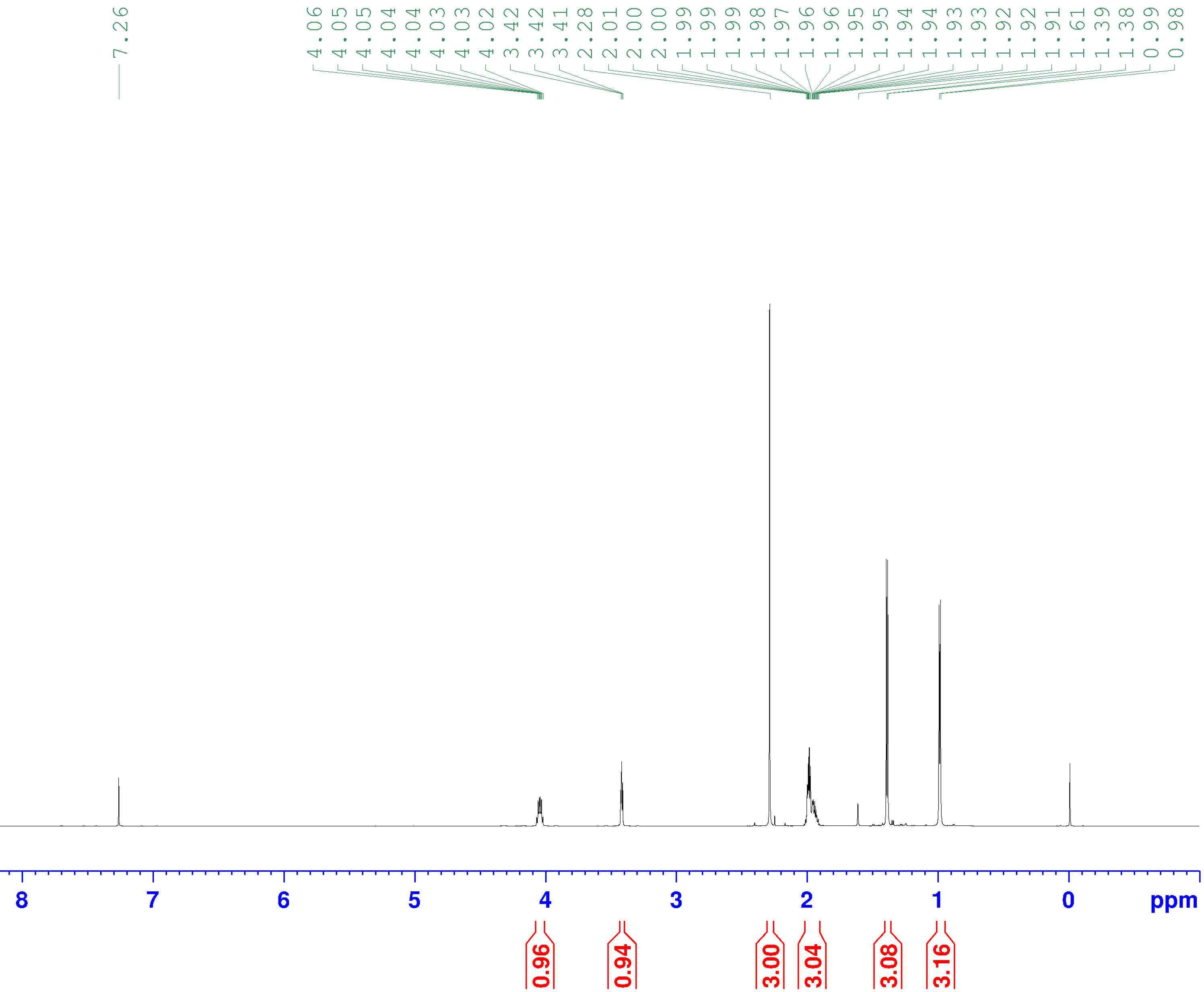

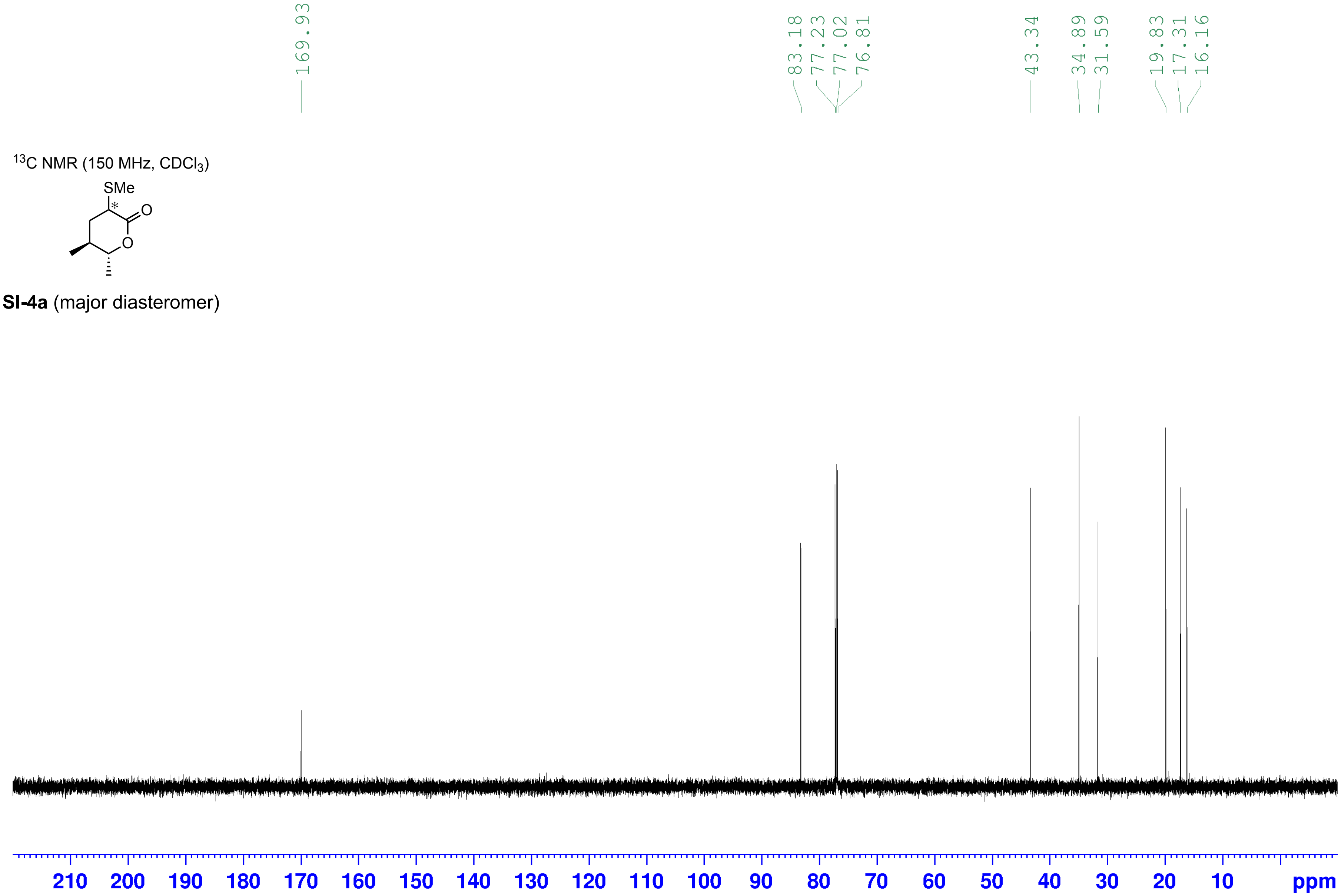

SI-4a (major diasteromer)

^{1}H NMR (600 MHz, $CDCl_3$)

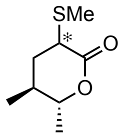

SI-4b (minor diasterome)

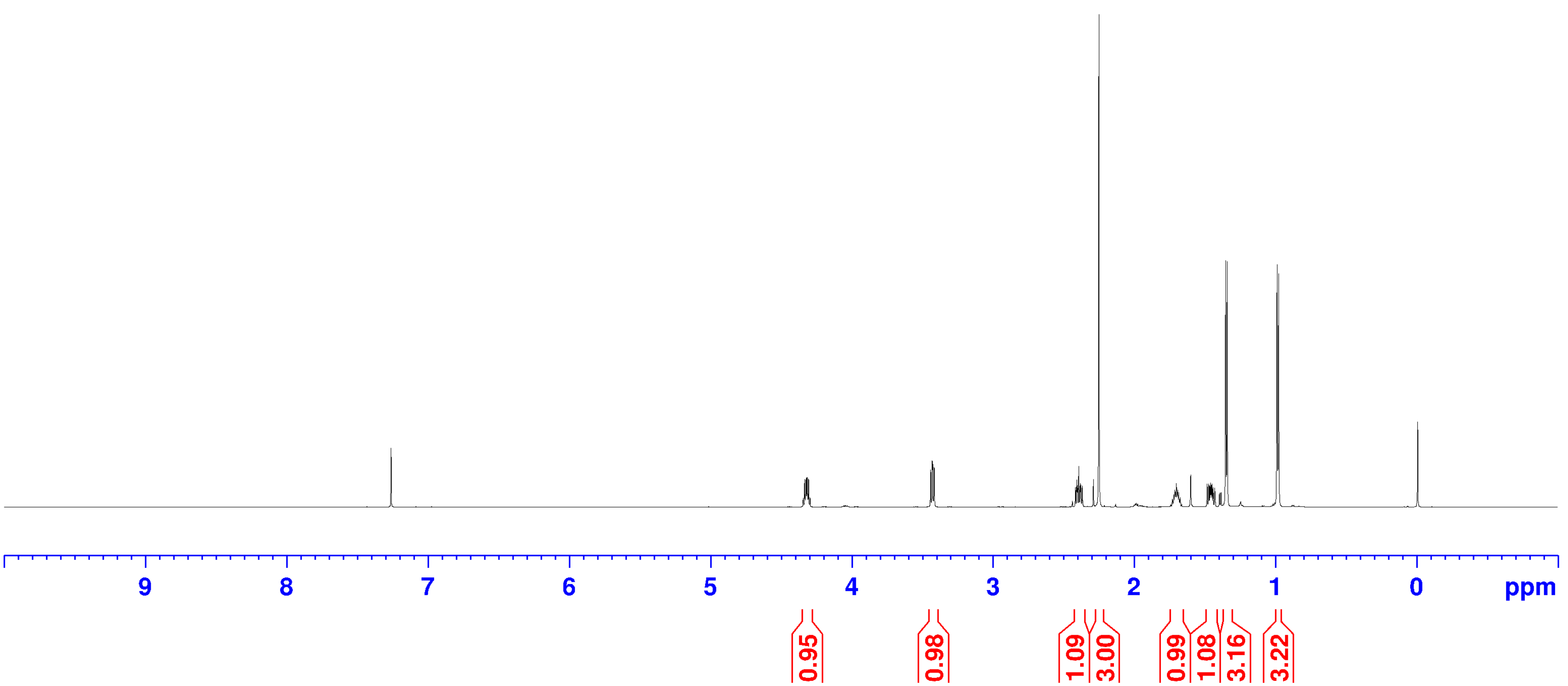

^{13}C NMR (150 MHz, $CDCl_3$)

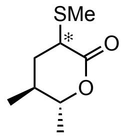

SI-4b (minor diasteromer)

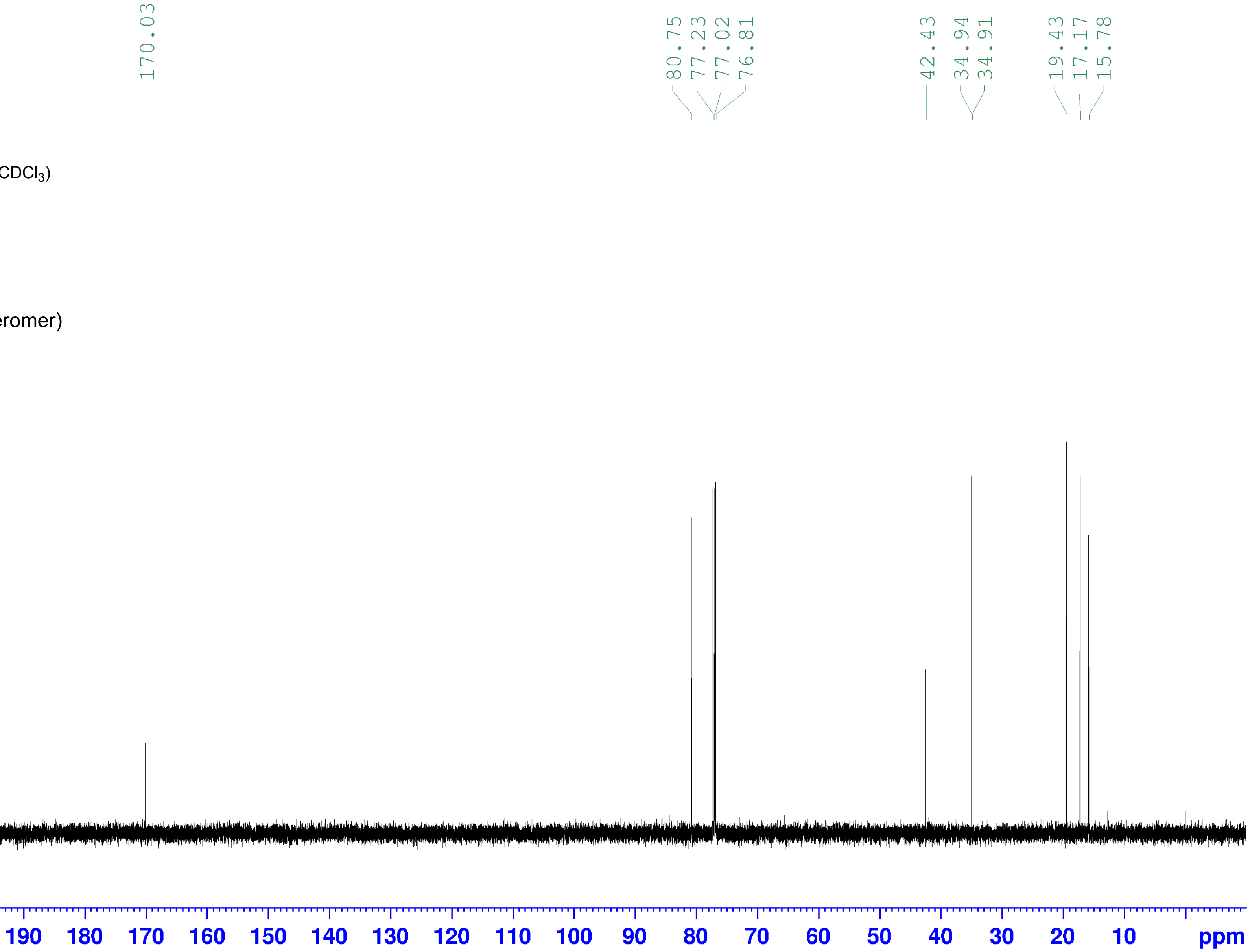

^{1}H NMR (600 MHz, $CDCl_3$)

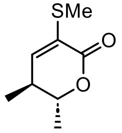

20

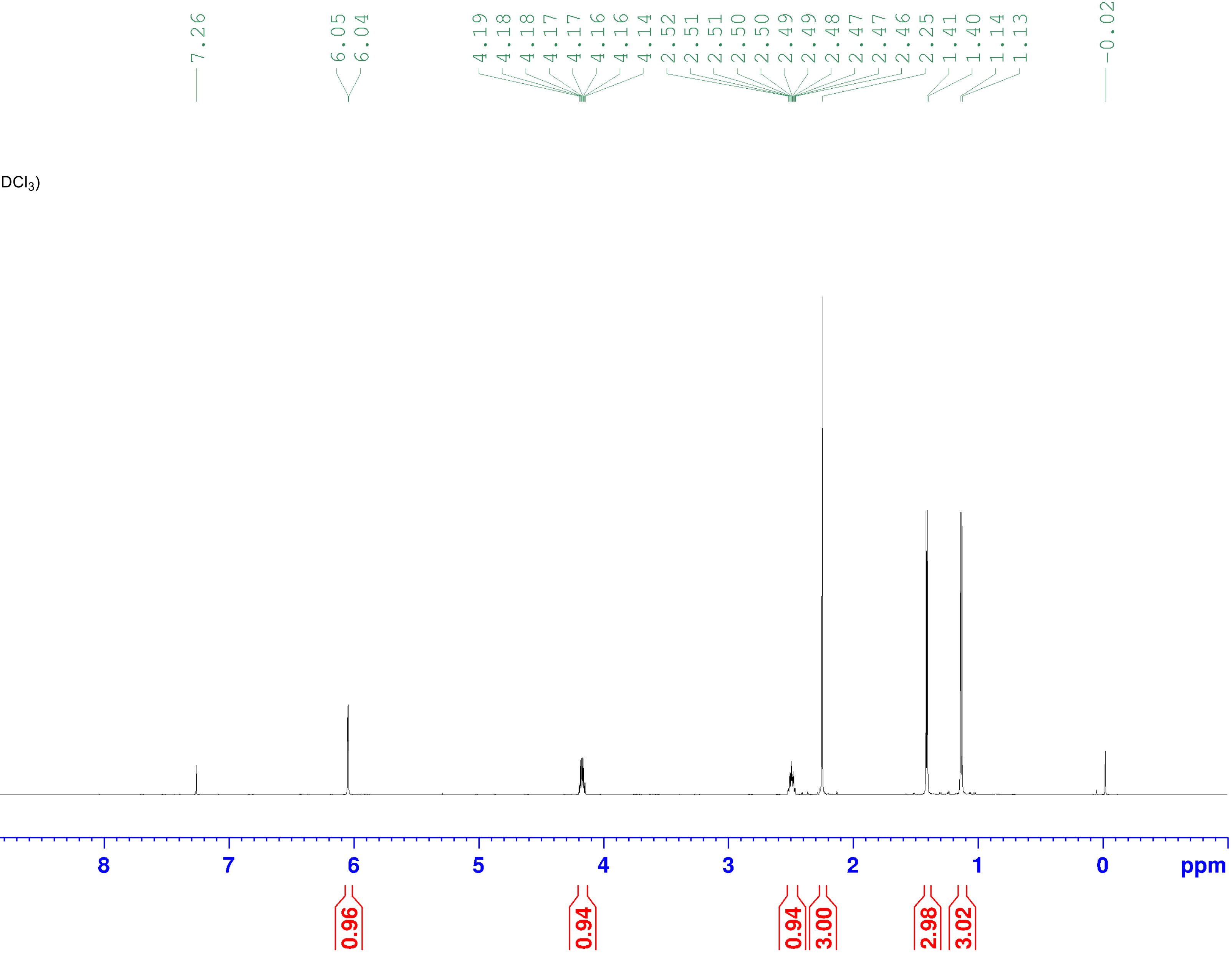

^{13}C NMR (150 MHz, $CDCl_3$)

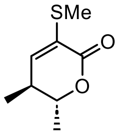

20

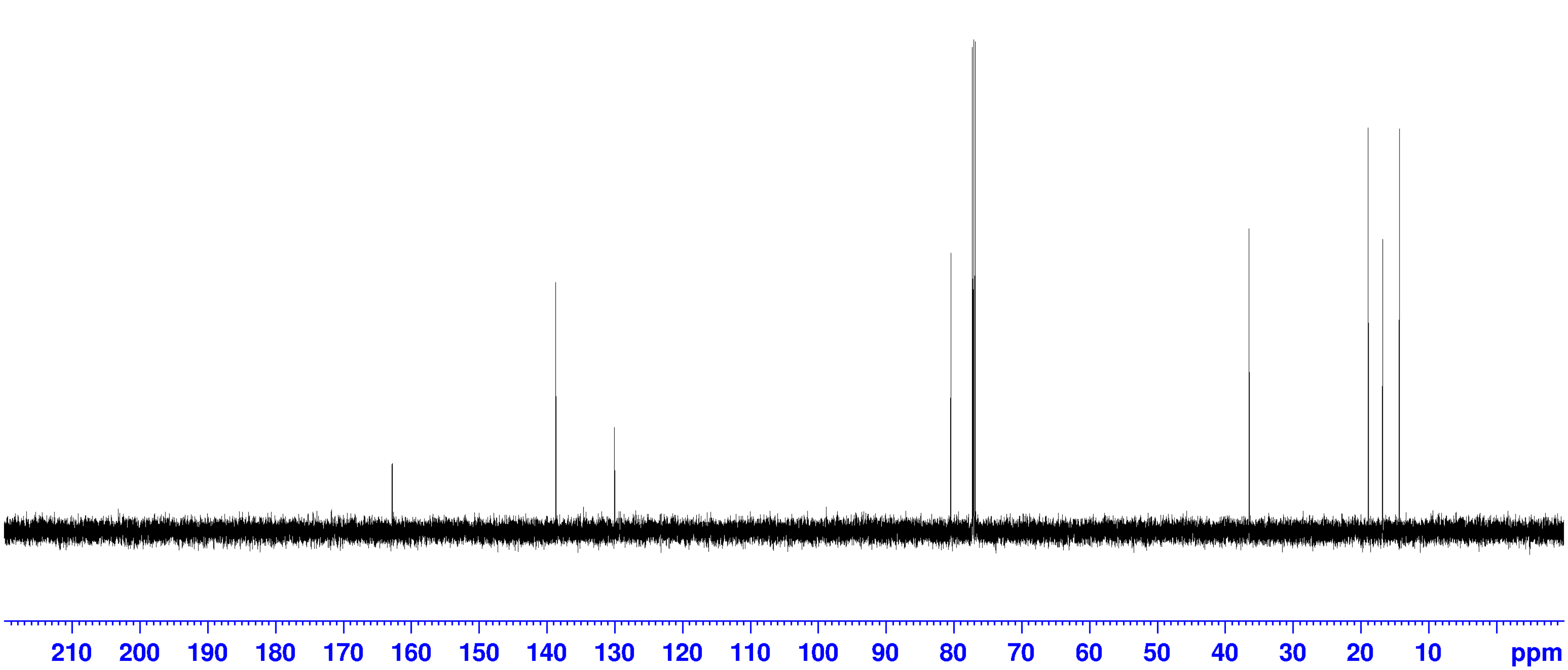

^{1}H NMR (600 MHz, CD_2Cl_2)

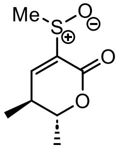

21 (*dr* 11:1)

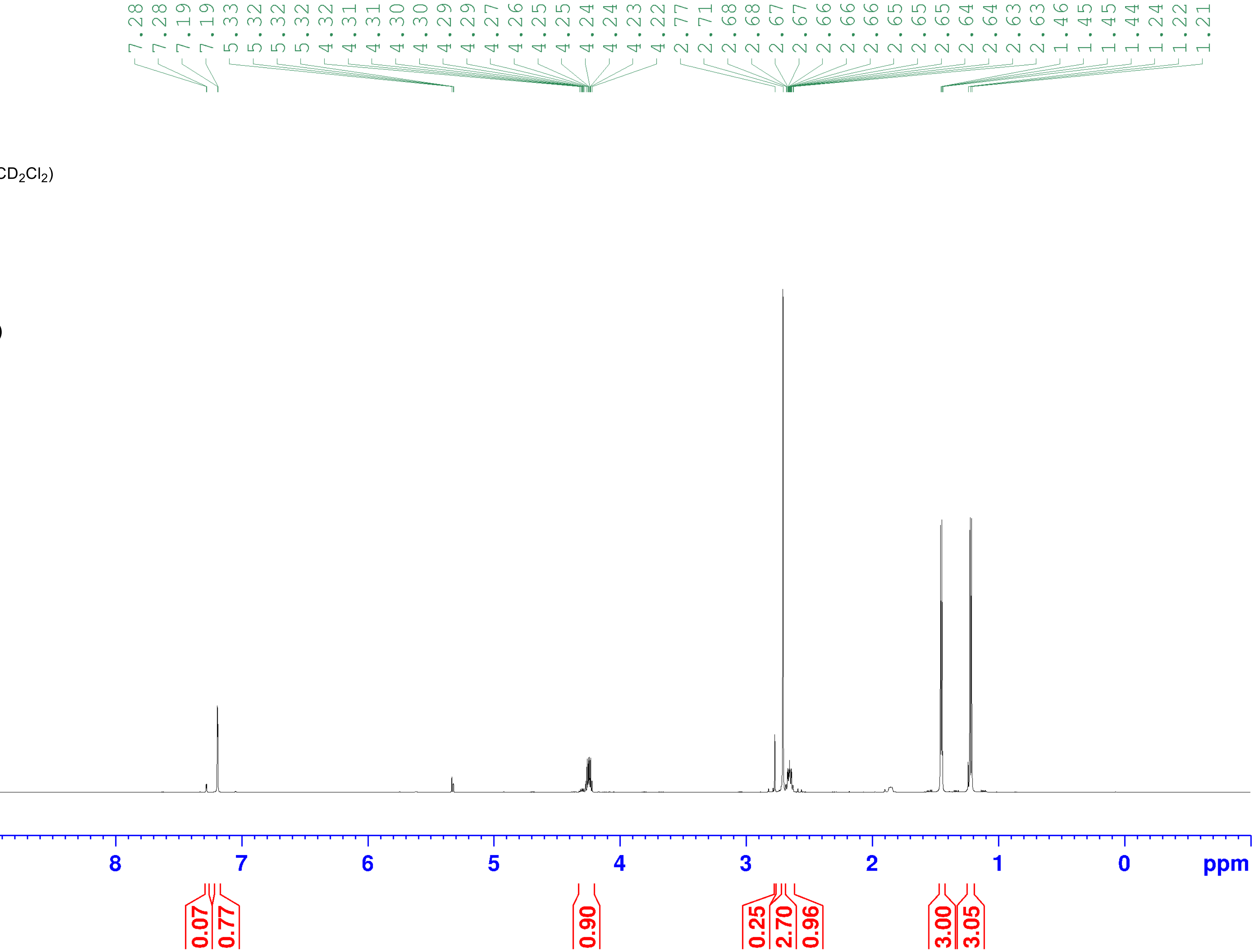

^{13}C NMR (150 MHz, CD_2Cl_2)

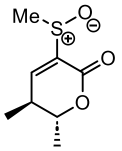

21 (*dr* 11:1)

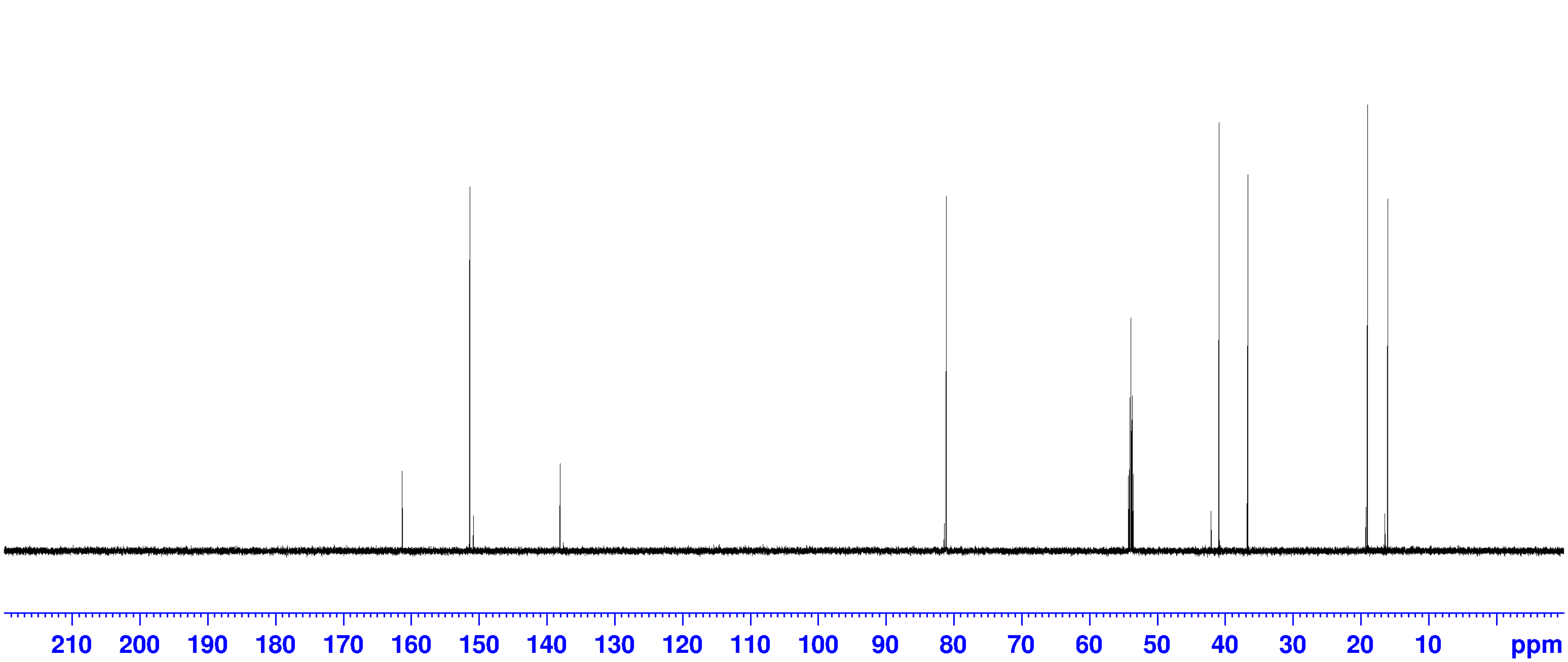

^{1}H NMR (600 MHz, $CDCl_3$)

22

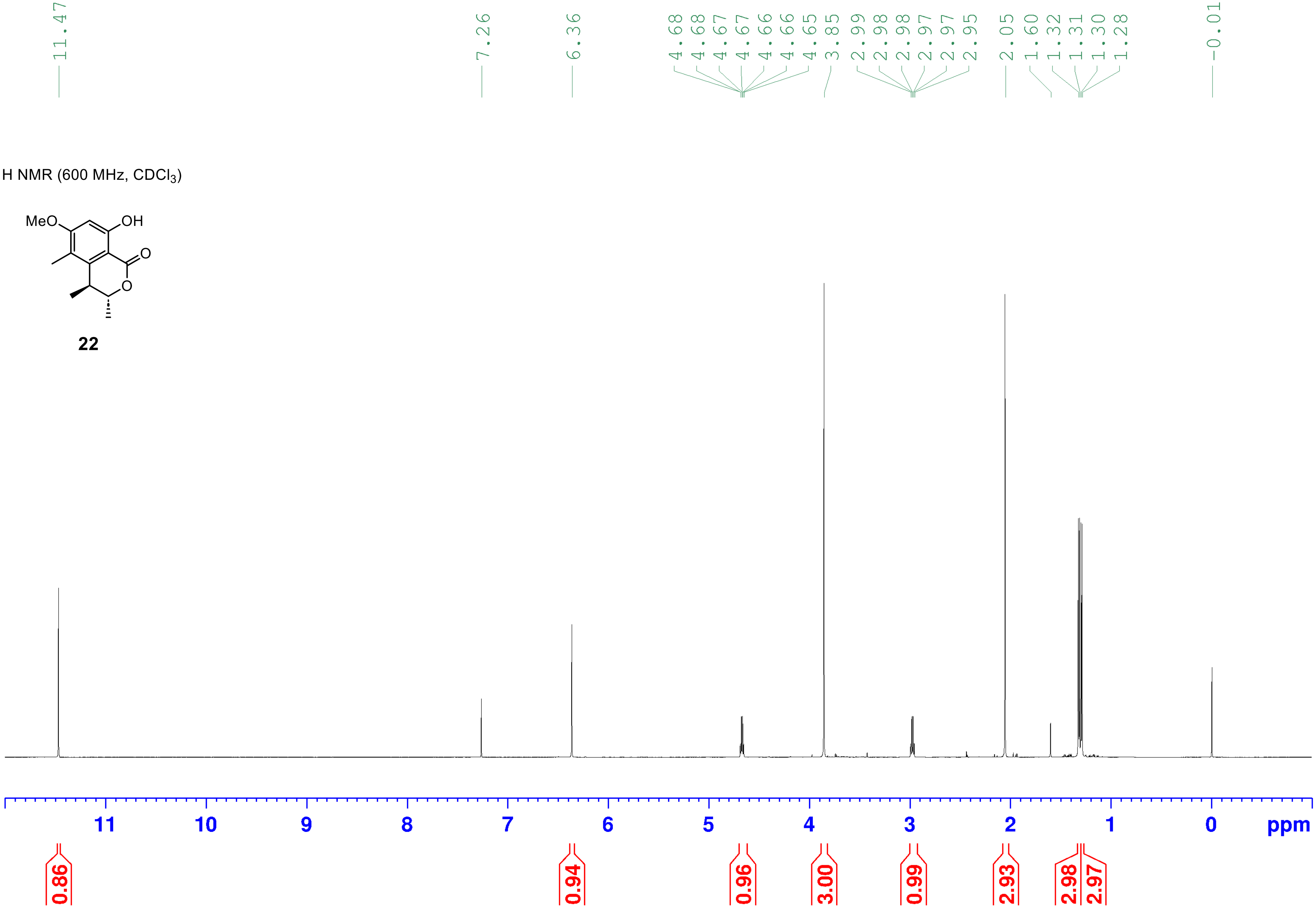

^{13}C NMR (150 MHz, CDCl$_3$)

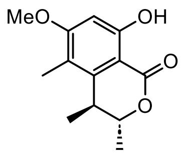

22

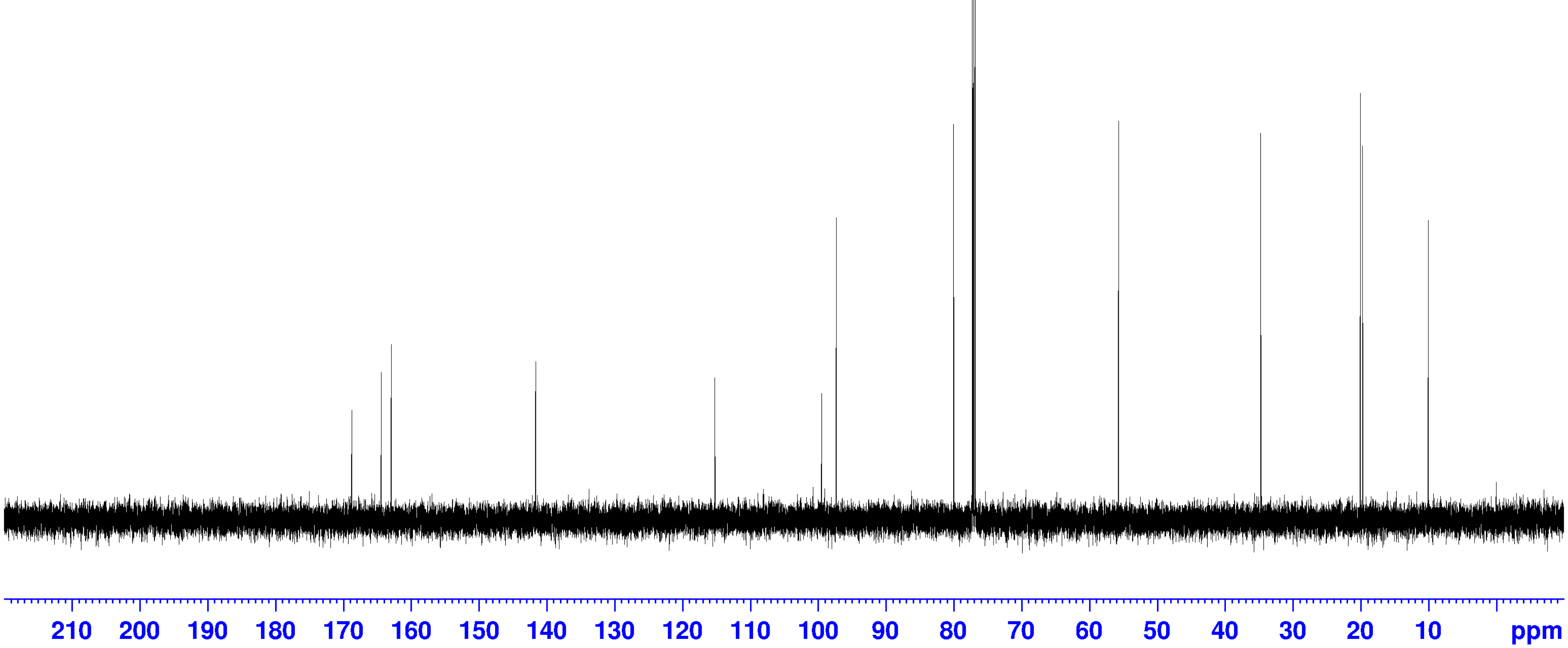

^{1}H NMR (600 MHz, $CDCl_3$)

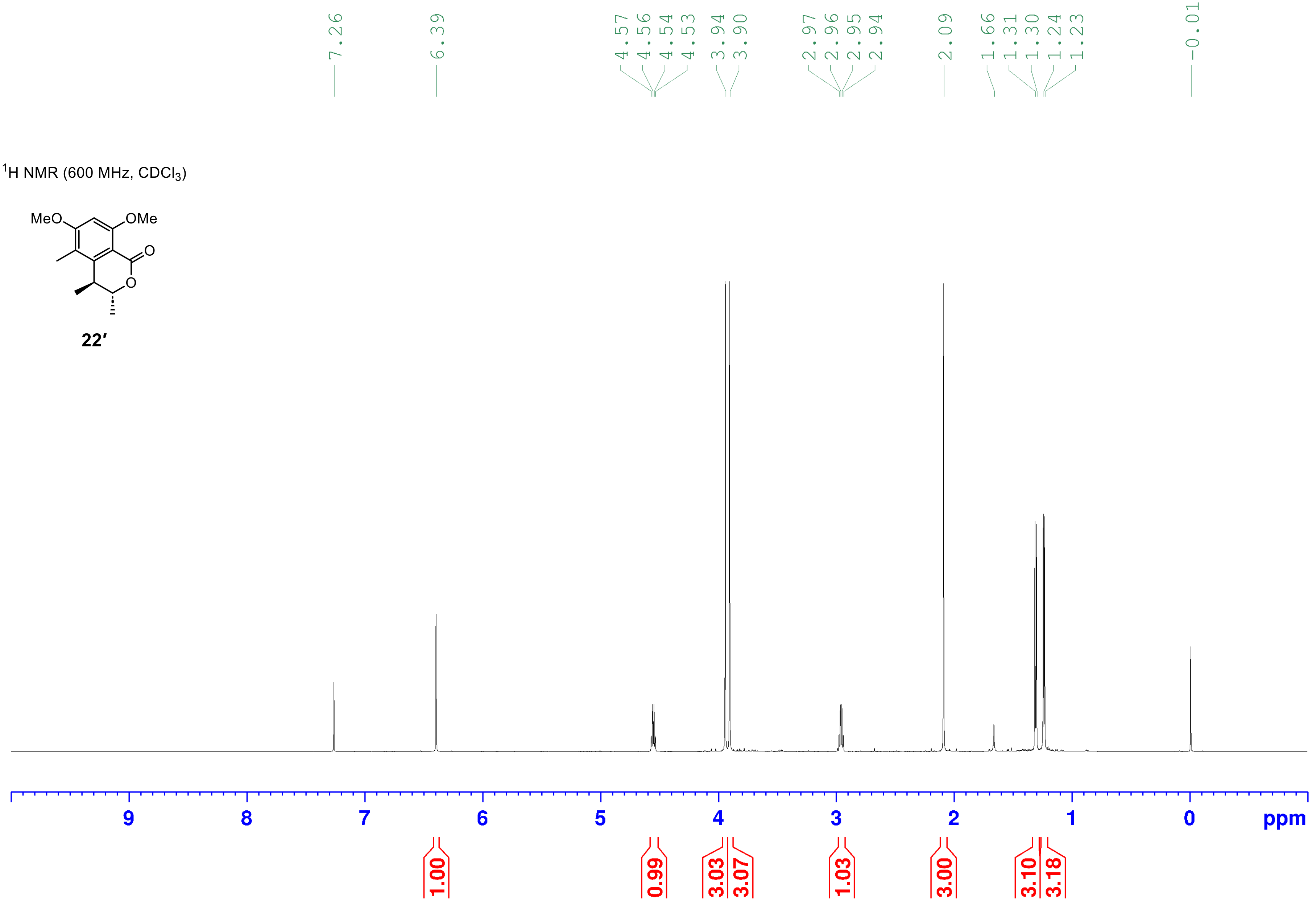

22′

^{13}C NMR (150 MHz, $CDCl_3$)

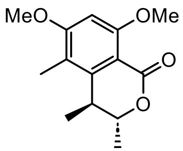

22′

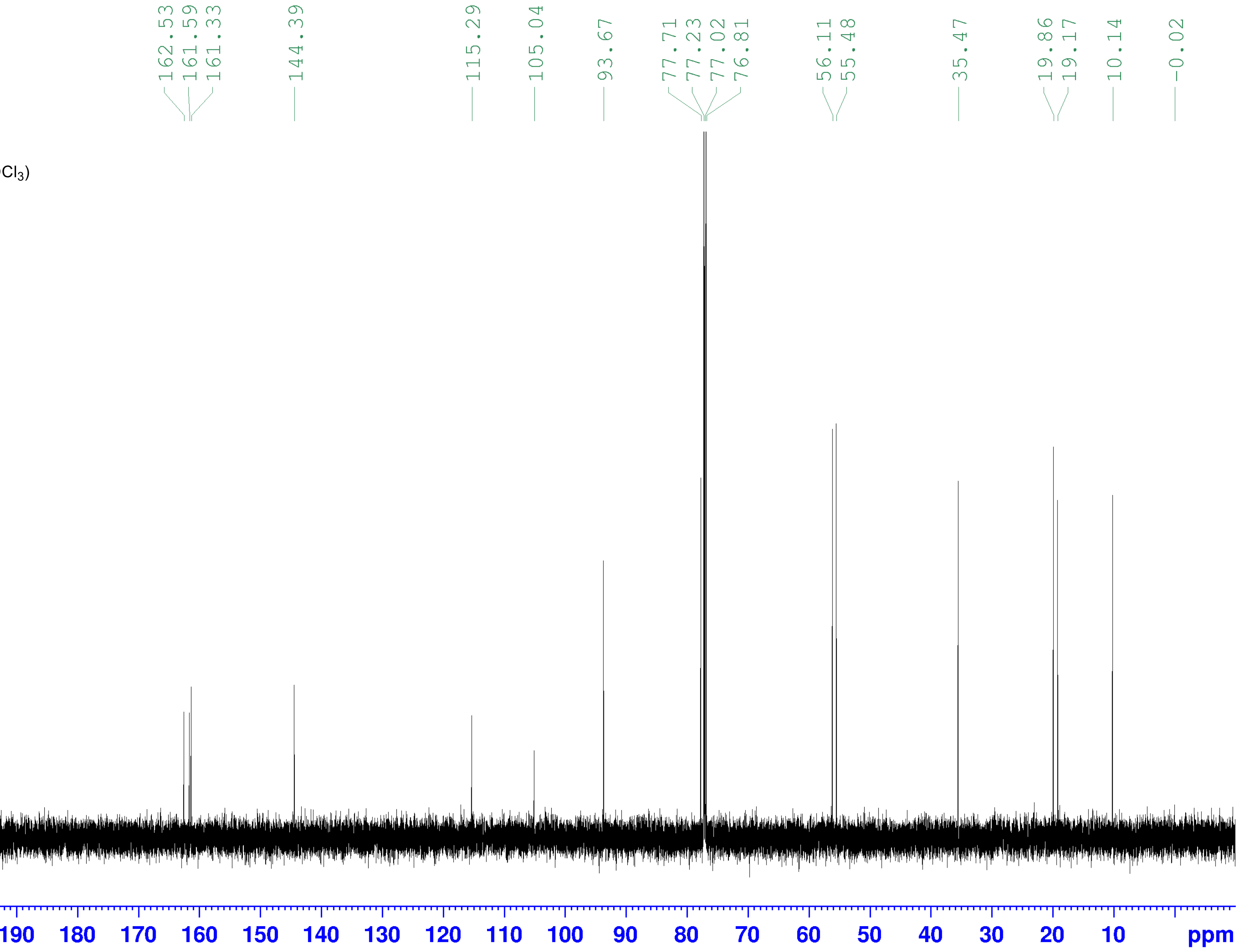

^{1}H NMR (600 MHz, $CDCl_3$)

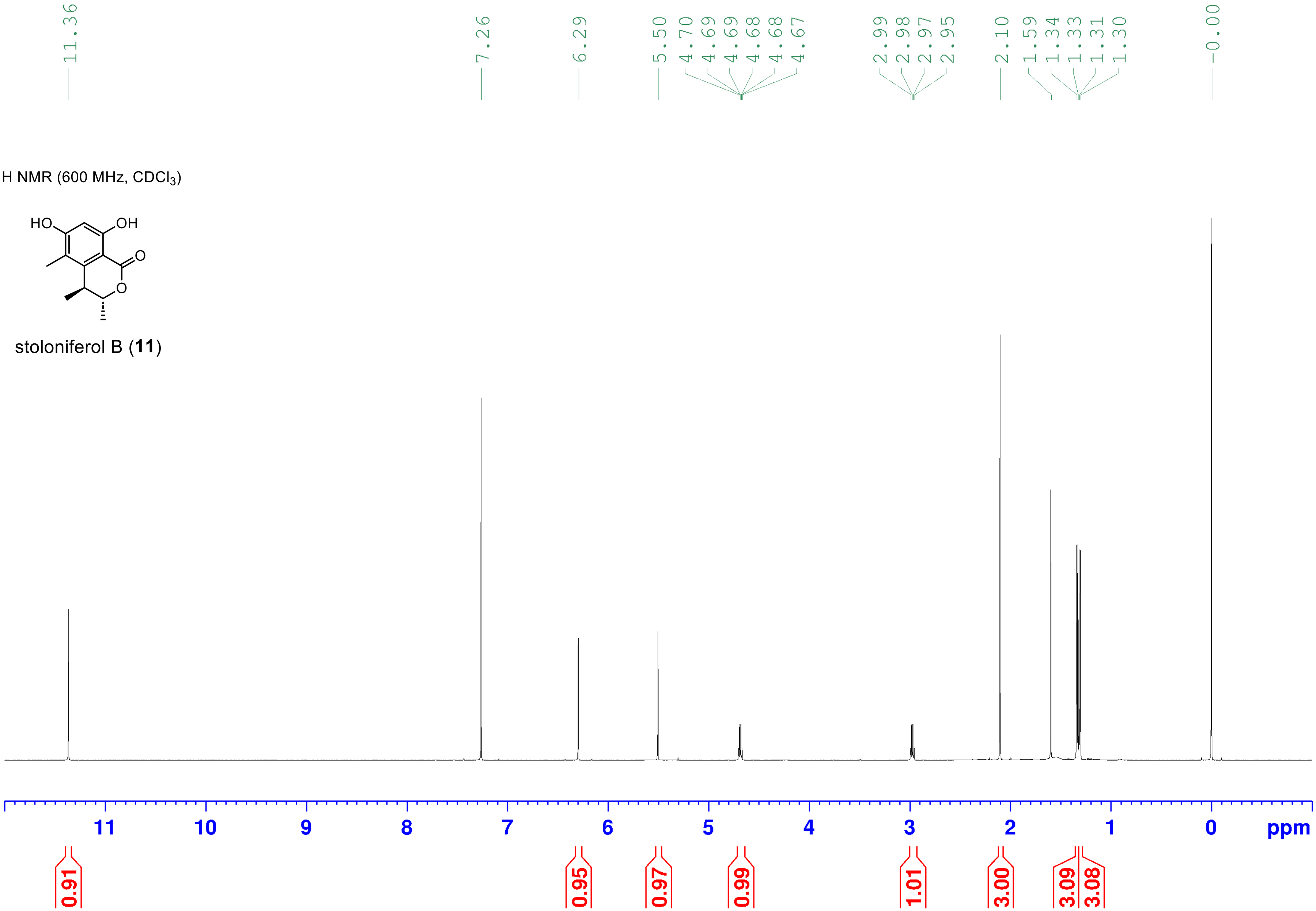

^{13}C NMR (150 MHz, $CDCl_3$)

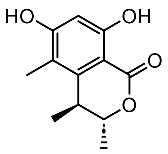

stoloniferol B (**11**)

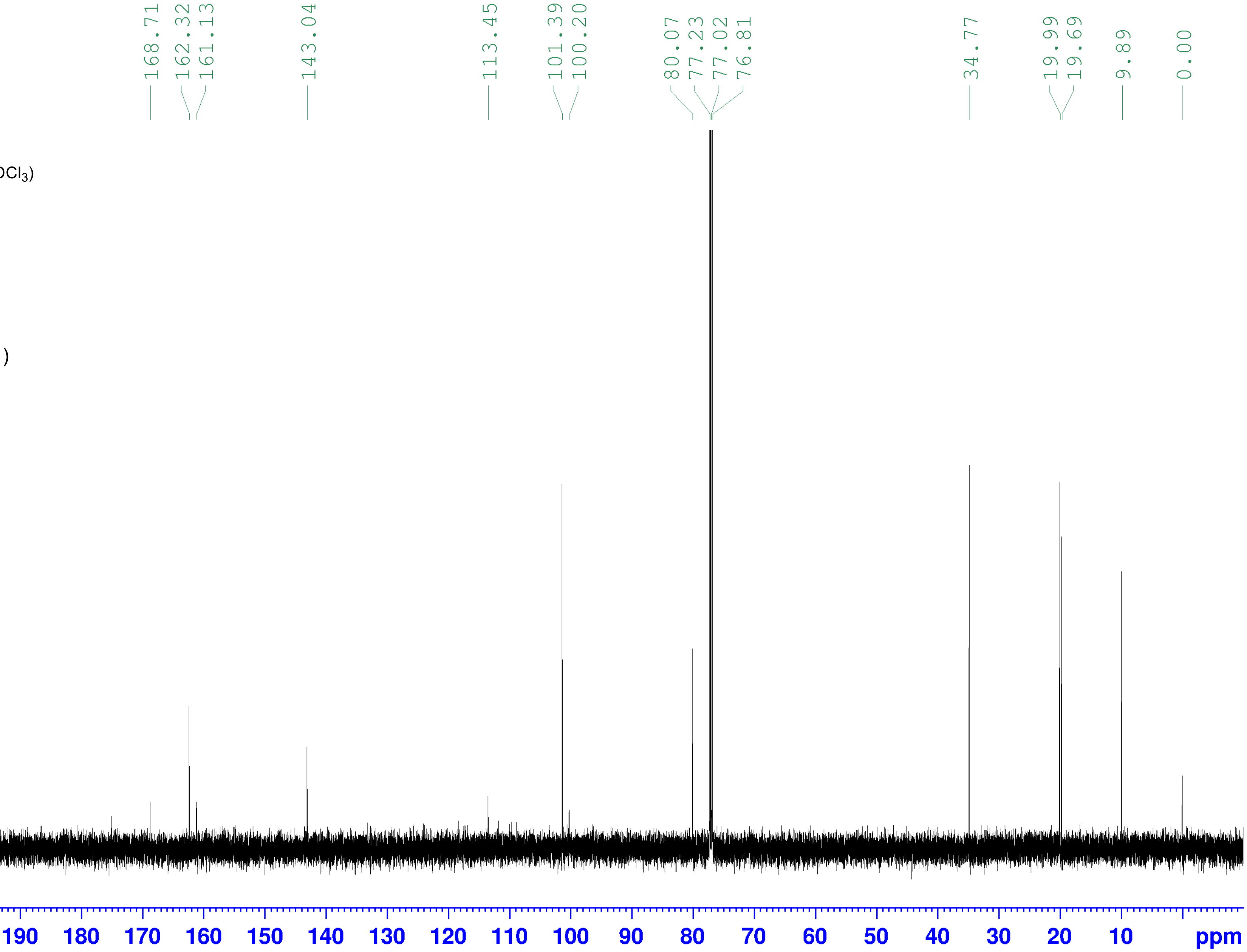

^{1}H NMR (600 MHz, $CDCl_3$)

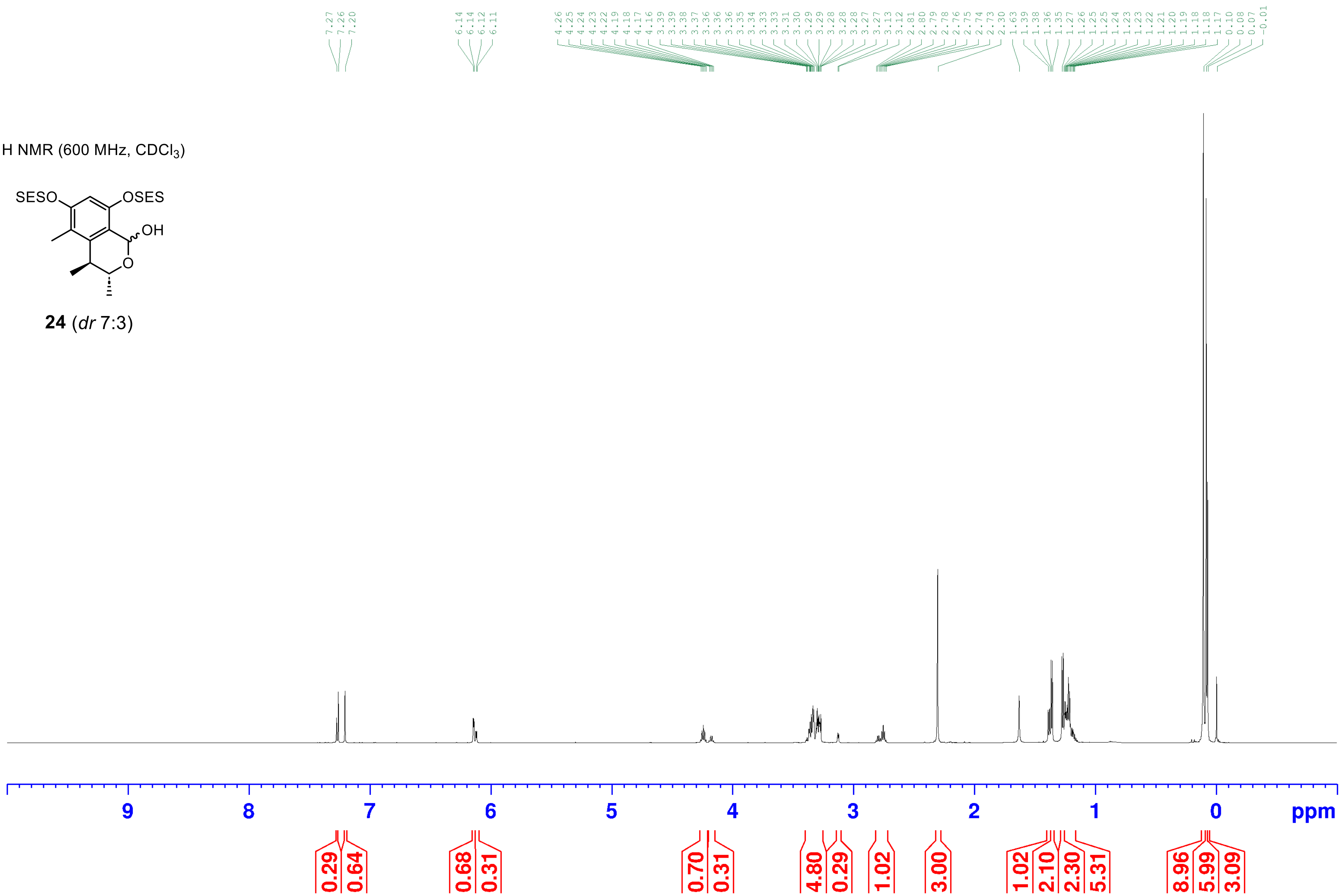

24 (*dr* 7:3)

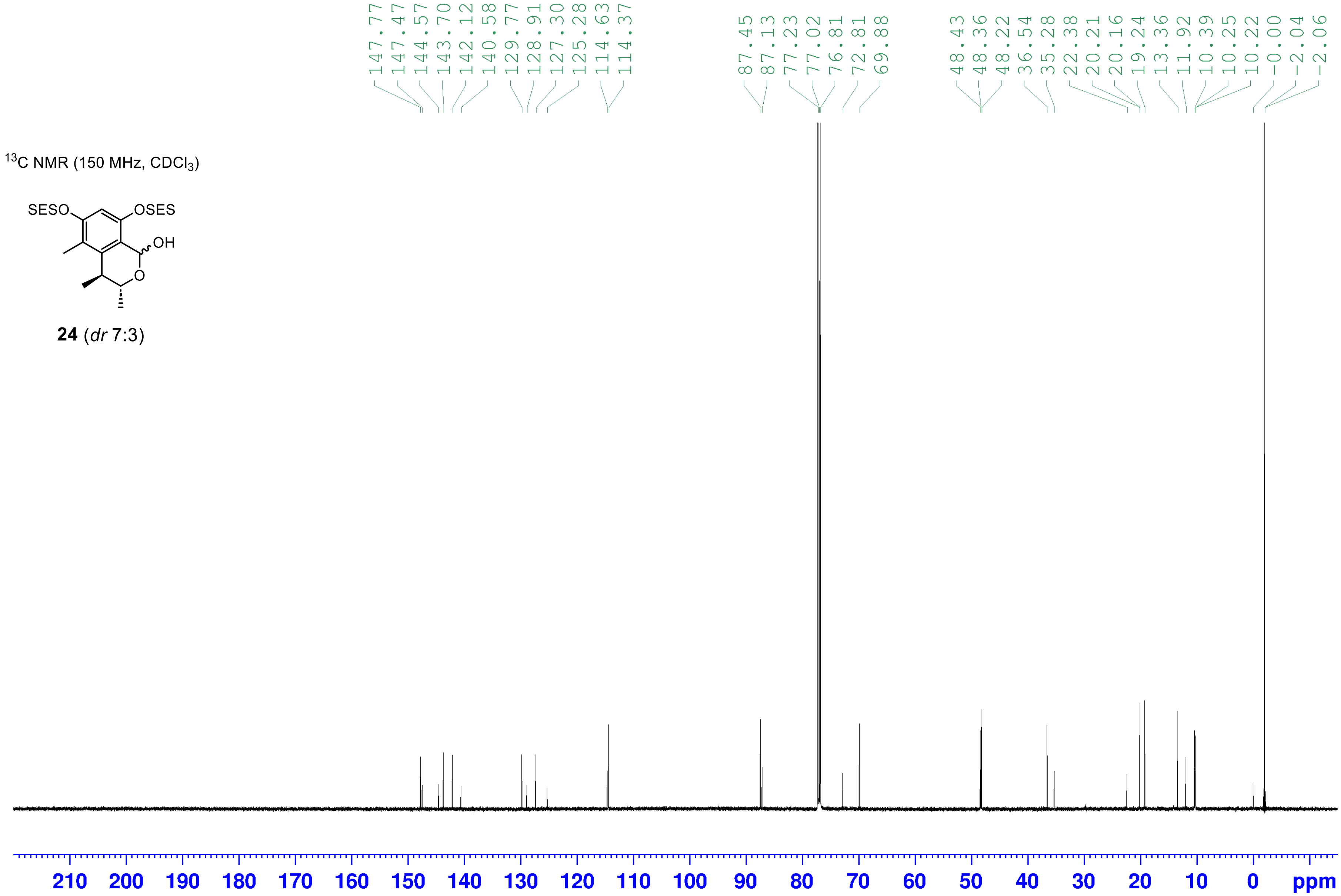

147.77
147.47
144.57
143.70
142.12
140.58
129.77
128.91
127.30
125.28
114.63
114.37
87.45
87.13
77.23
77.02
76.81
72.81
69.88
48.43
48.36
48.22
36.54
35.28
22.38
20.21
20.16
19.24
13.36
11.92
10.39
10.25
10.22
-0.00
-2.04
-2.06
13C NMR (150 MHz, CDCl3)
SESO
OSES
OH
O
210 200 190 180 170 160 150 140 130 120 110 100 90 80 70 60 50 40 30 20 10 0 ppm

24 (*dr* 7:3)

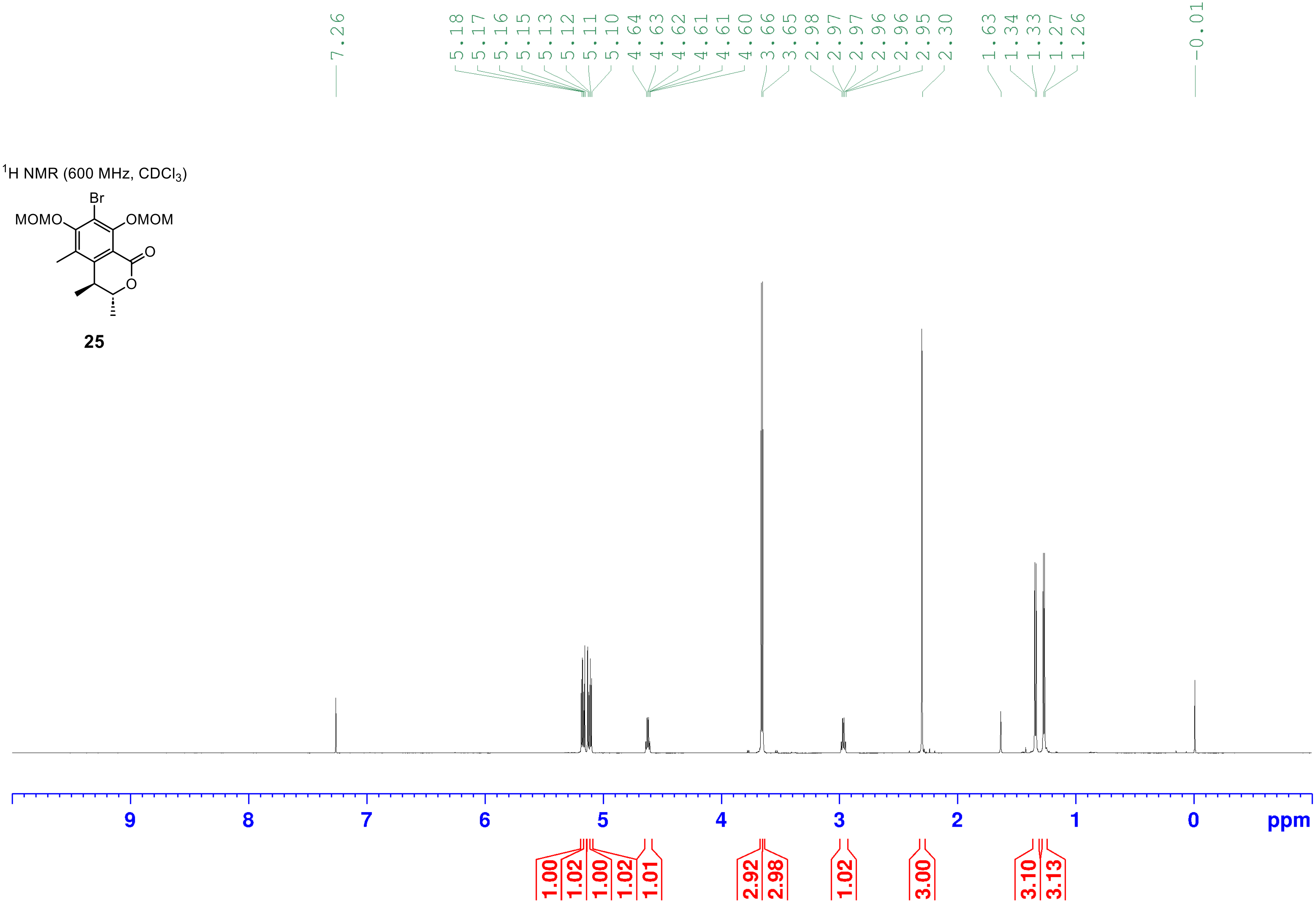
7.26
5.18
5.17
5.16
5.15
5.13
5.12
5.11
5.10
4.64
4.63
4.62
4.61
4.61
4.60
3.66
3.65
2.98
2.97
2.97
2.96
2.96
2.95
2.30
1.63
1.34
1.33
1.27
1.26
-0.01
¹H NMR (600 MHz, CDCl₃)
9
8
7
6
5
4
3
2
1
0
ppm
1.00
1.02
1.00
1.02
1.01
2.92
2.98
1.02
3.00
3.10
3.13
Br
MOMO
OMOM
O
O

25

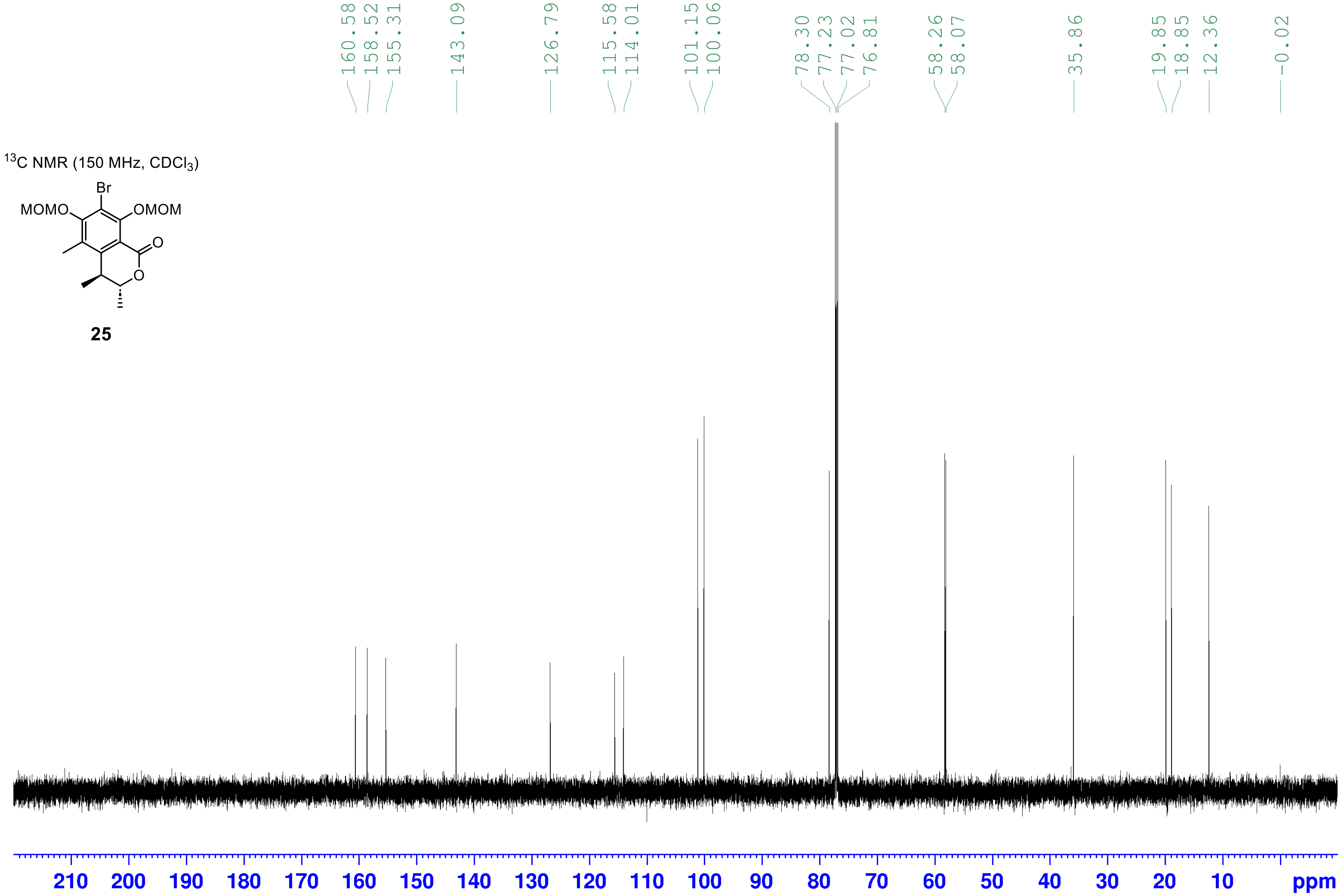
^{13}C NMR (150 MHz, CDCl$_3$)
Br
MOMO
OMOM
O
O
25
160.58
158.52
155.31
143.09
126.79
115.58
114.01
101.15
100.06
78.30
77.23
77.02
76.81
58.26
58.07
35.86
19.85
18.85
12.36
-0.02
210
200
190
180
170
160
150
140
130
120
110
100
90
80
70
60
50
40
30
20
10
ppm

^1H NMR (600 MHz, $CDCl_3$)

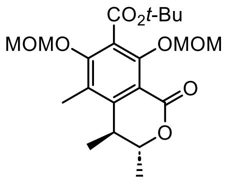

26

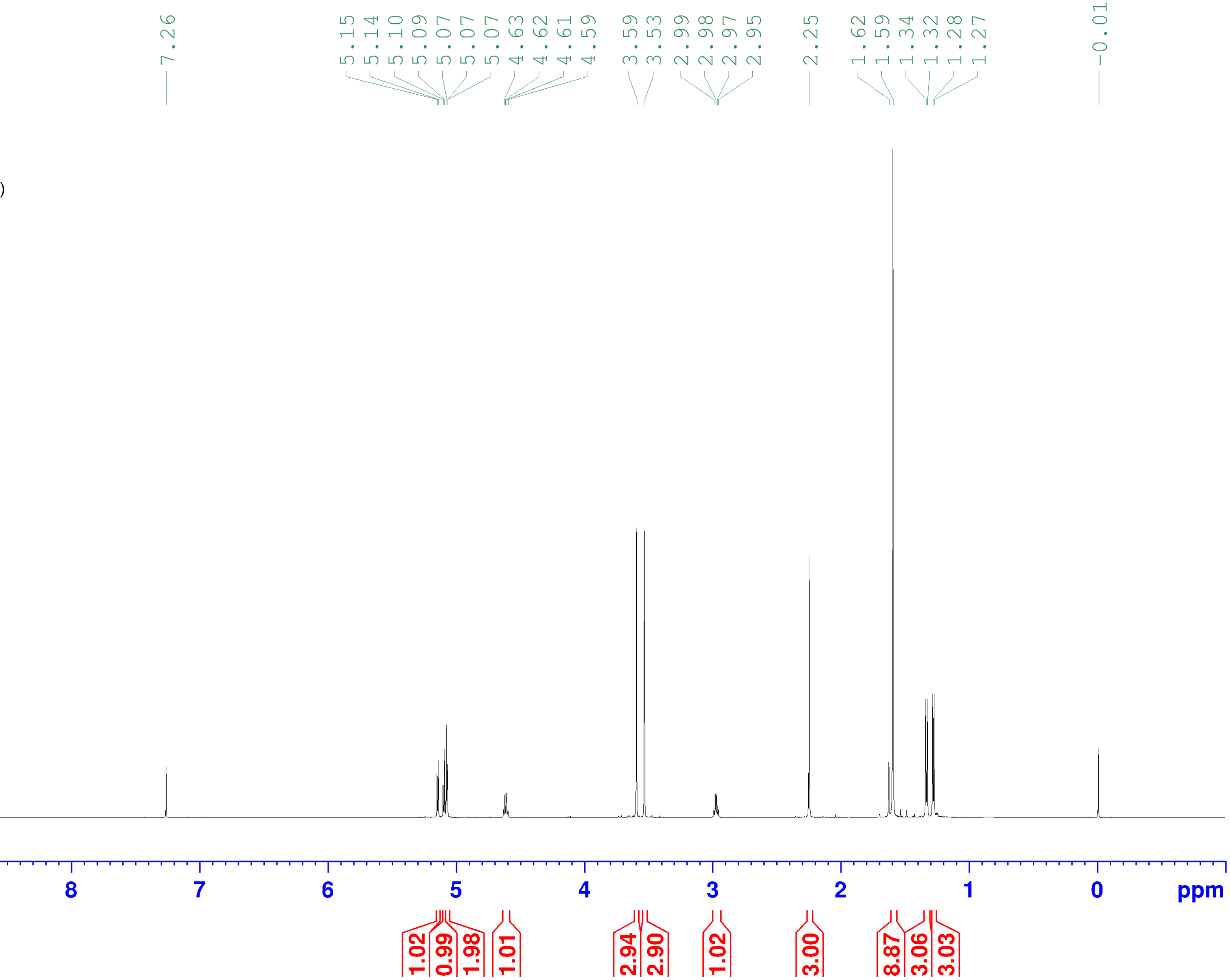

^{13}C NMR (150 MHz, $CDCl_3$)

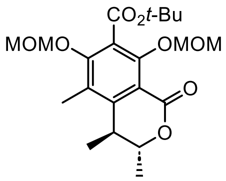

26

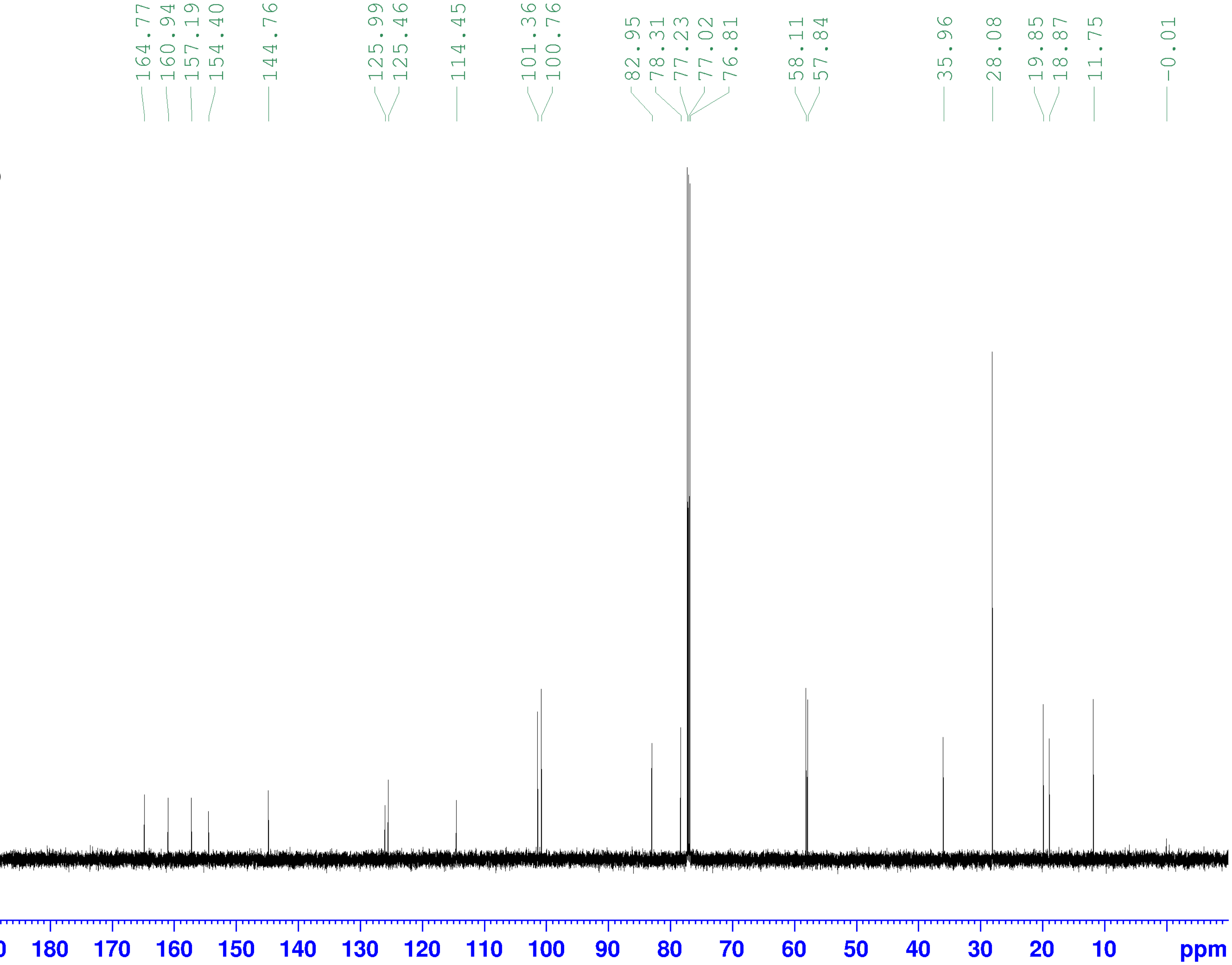

^1H NMR (600 MHz, $CDCl_3$)

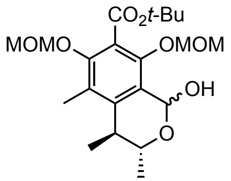

SI-5 (*dr* 4:1)

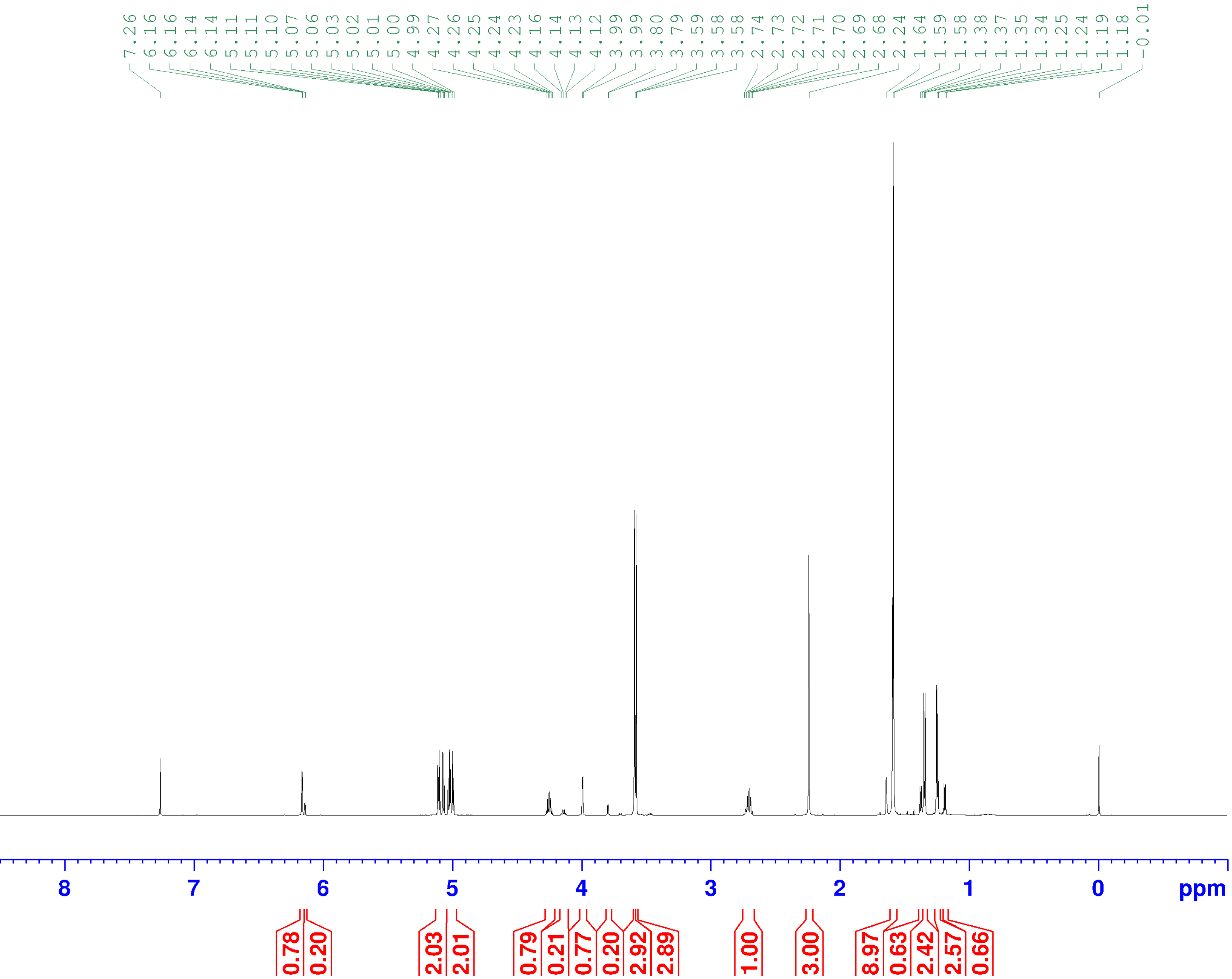

^{13}C NMR (150 MHz, $CDCl_3$)

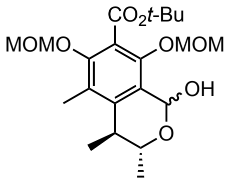

SI-5 (*dr* 4:1)

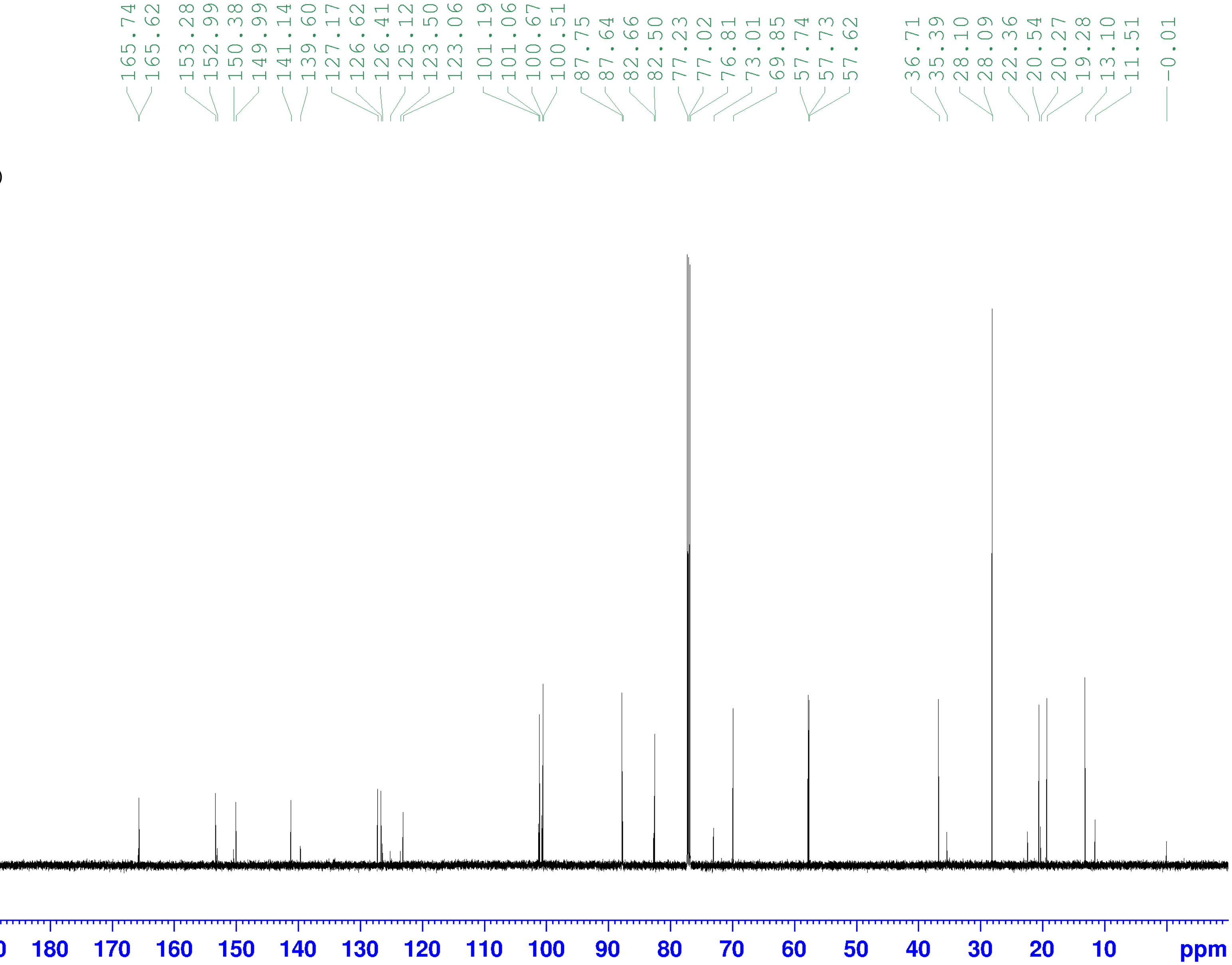

^{1}H NMR (600 MHz, $CDCl_3$)

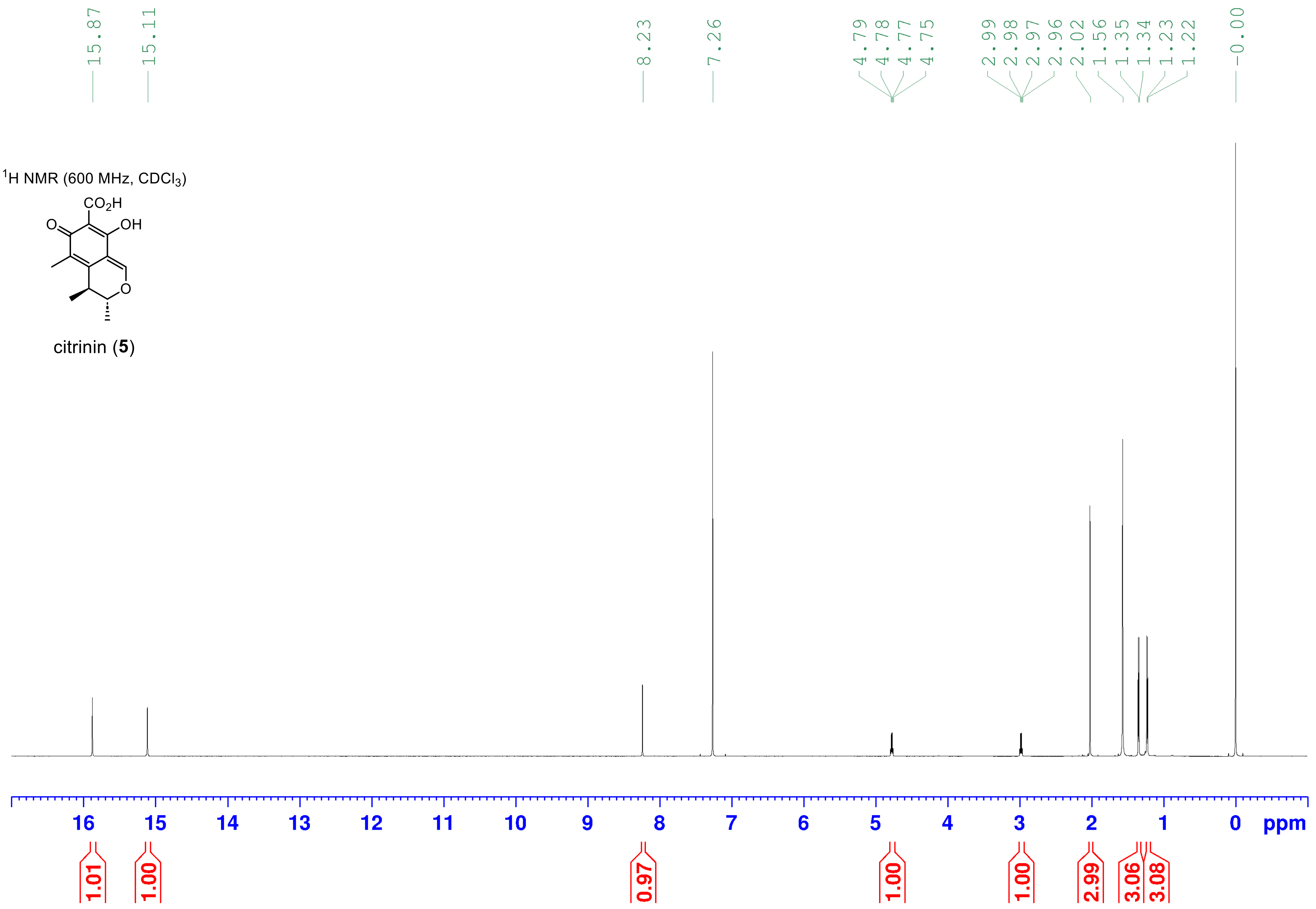

citrinin (**5**)

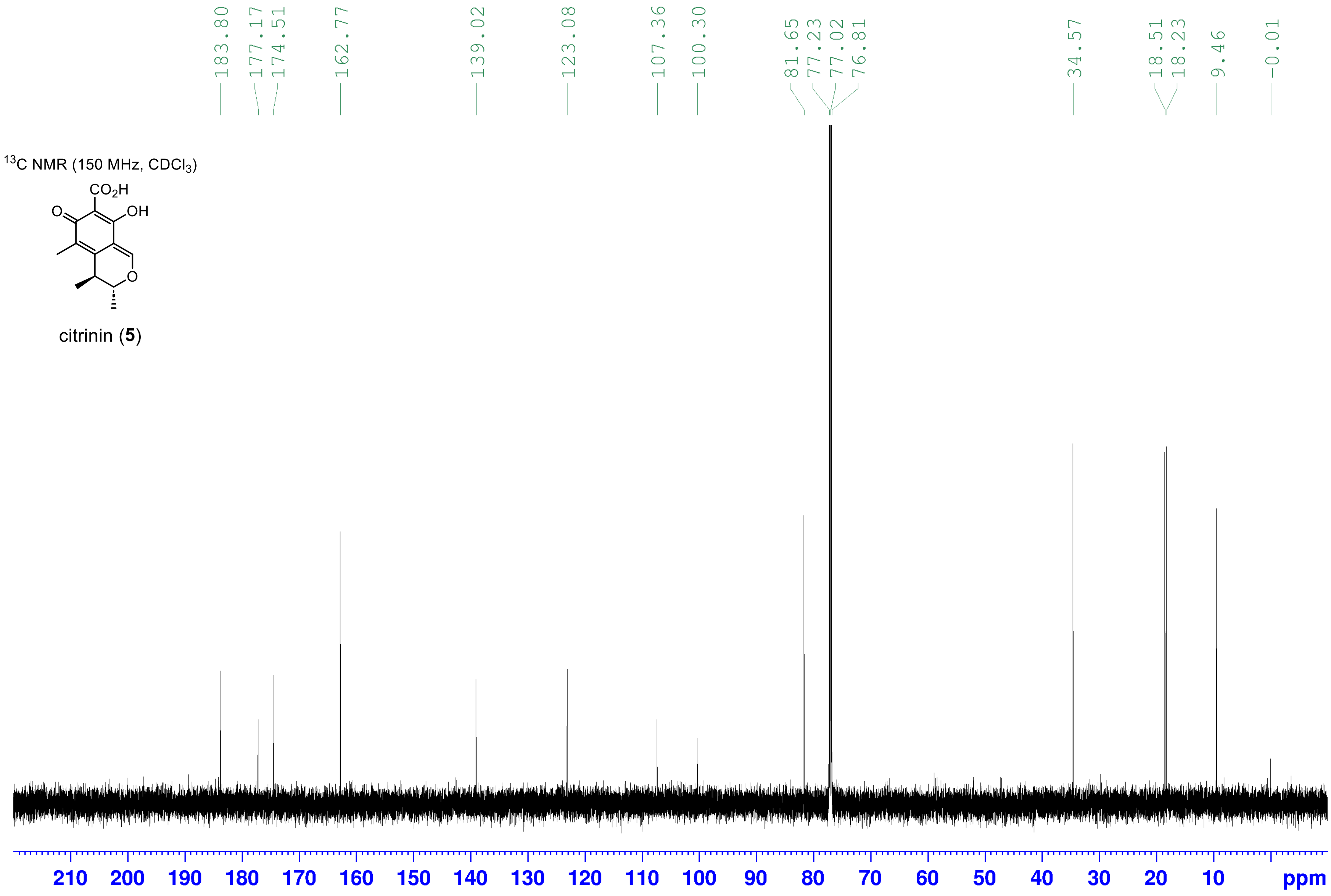
183.80
177.17
174.51
162.77
139.02
123.08
107.36
100.30
81.65
77.23
77.02
76.81
34.57
18.51
18.23
9.46
-0.01
^{13}C NMR (150 MHz, $CDCl_3$)
CO_2H
O
OH
O
citrinin (**5**)
210 200 190 180 170 160 150 140 130 120 110 100 90 80 70 60 50 40 30 20 10 ppm

^1H NMR (600 MHz, $CDCl_3$)

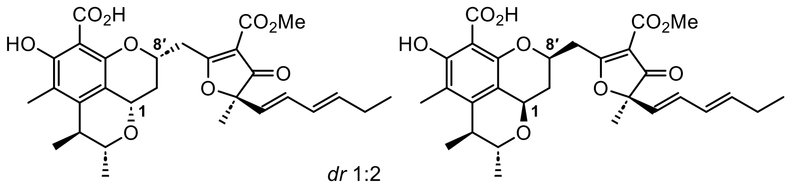

27 *desired* (*minor*) 1,8′-bis-*epi*-**27** *undesired* (*major*)

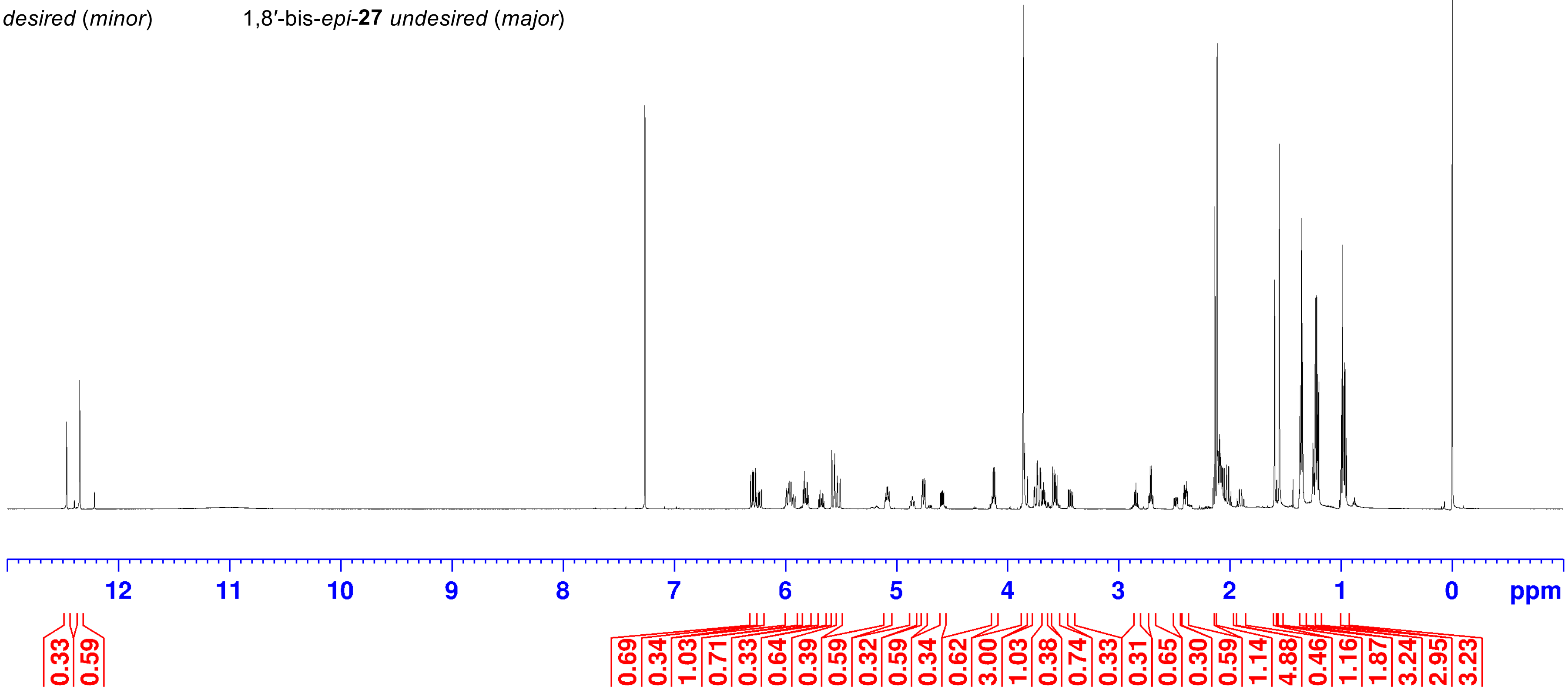

1D NOESY spectrum of 27

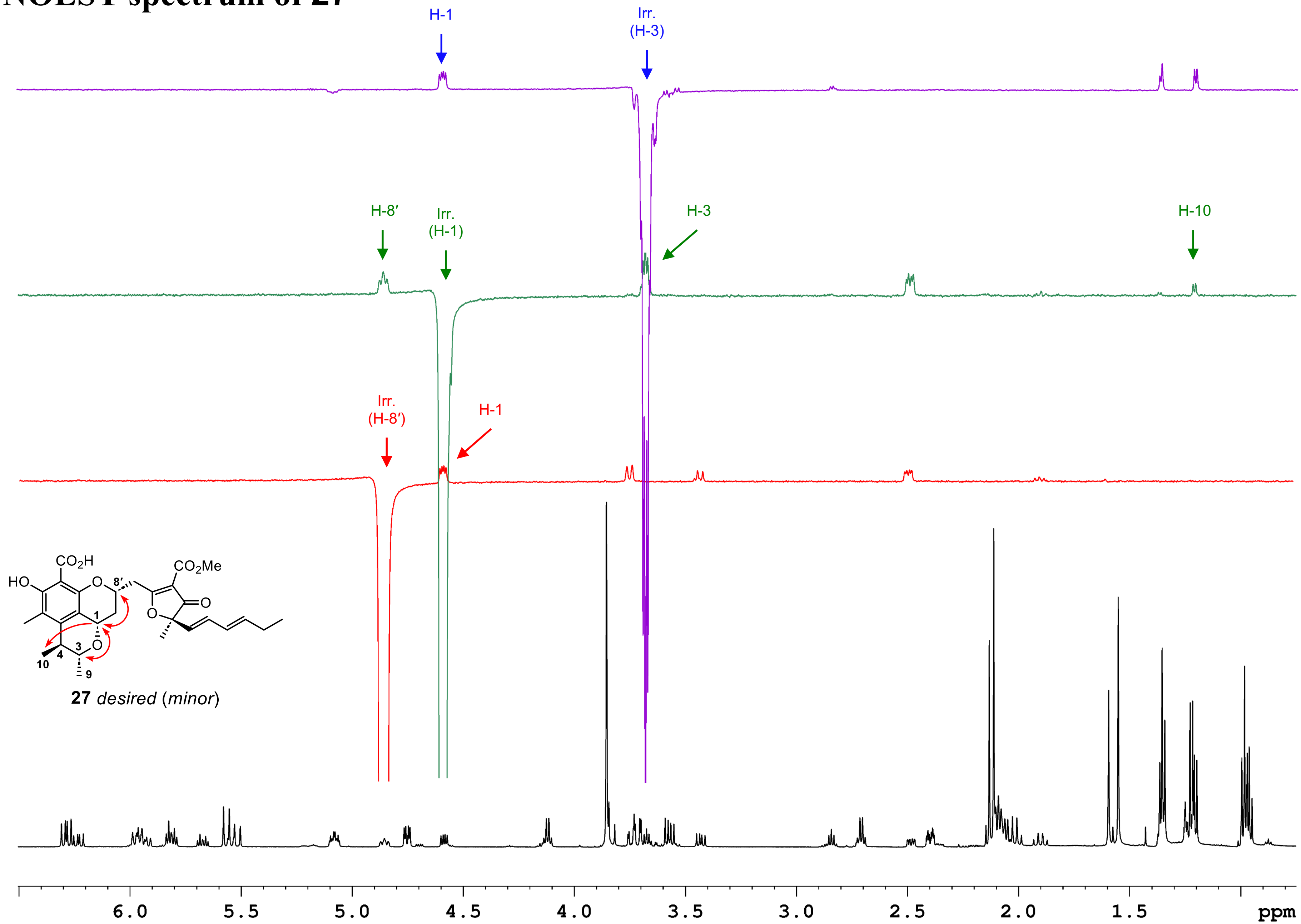

1D NOESY spectrum of 1,8-bis-*epi*-**27**.

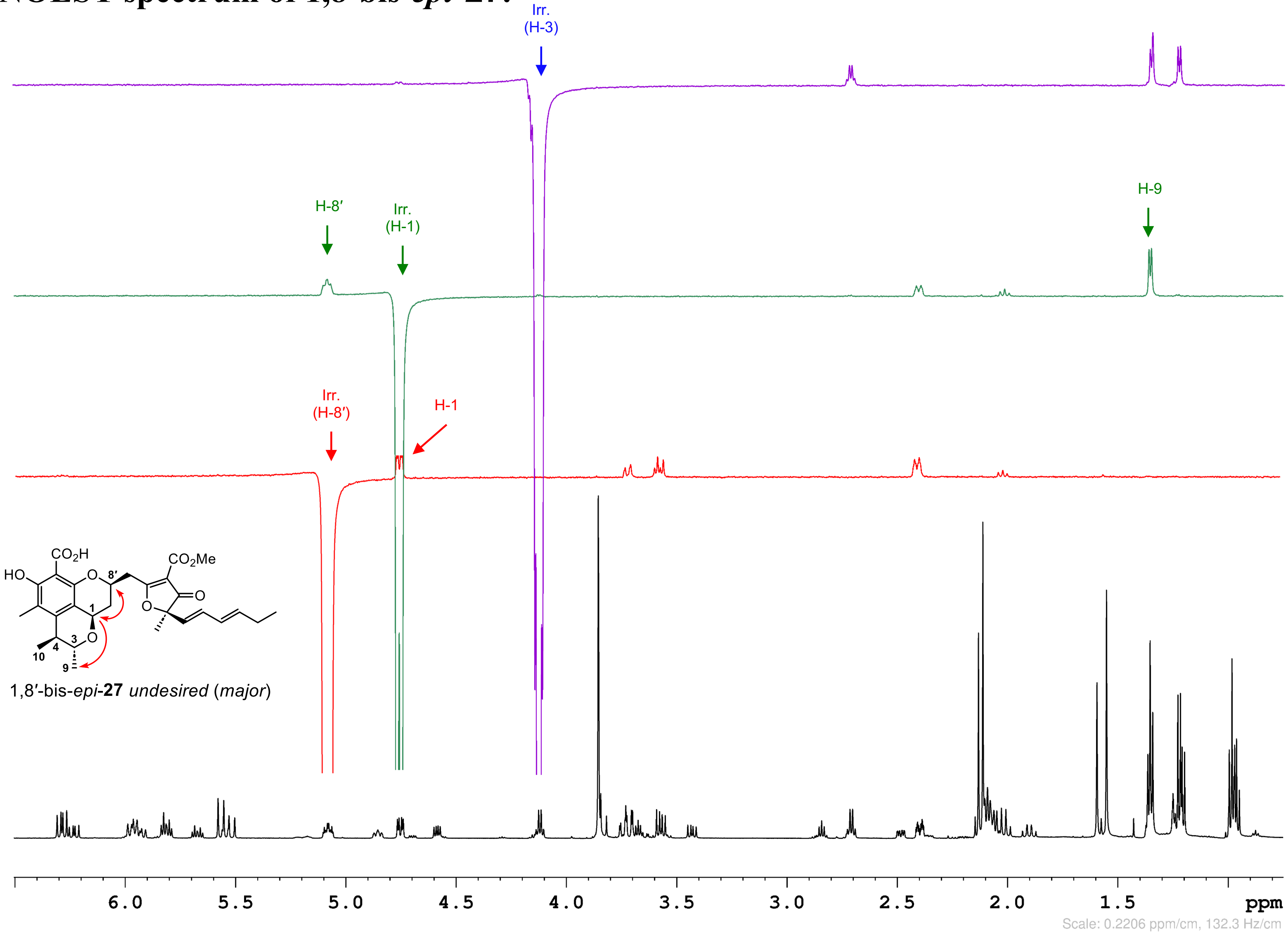

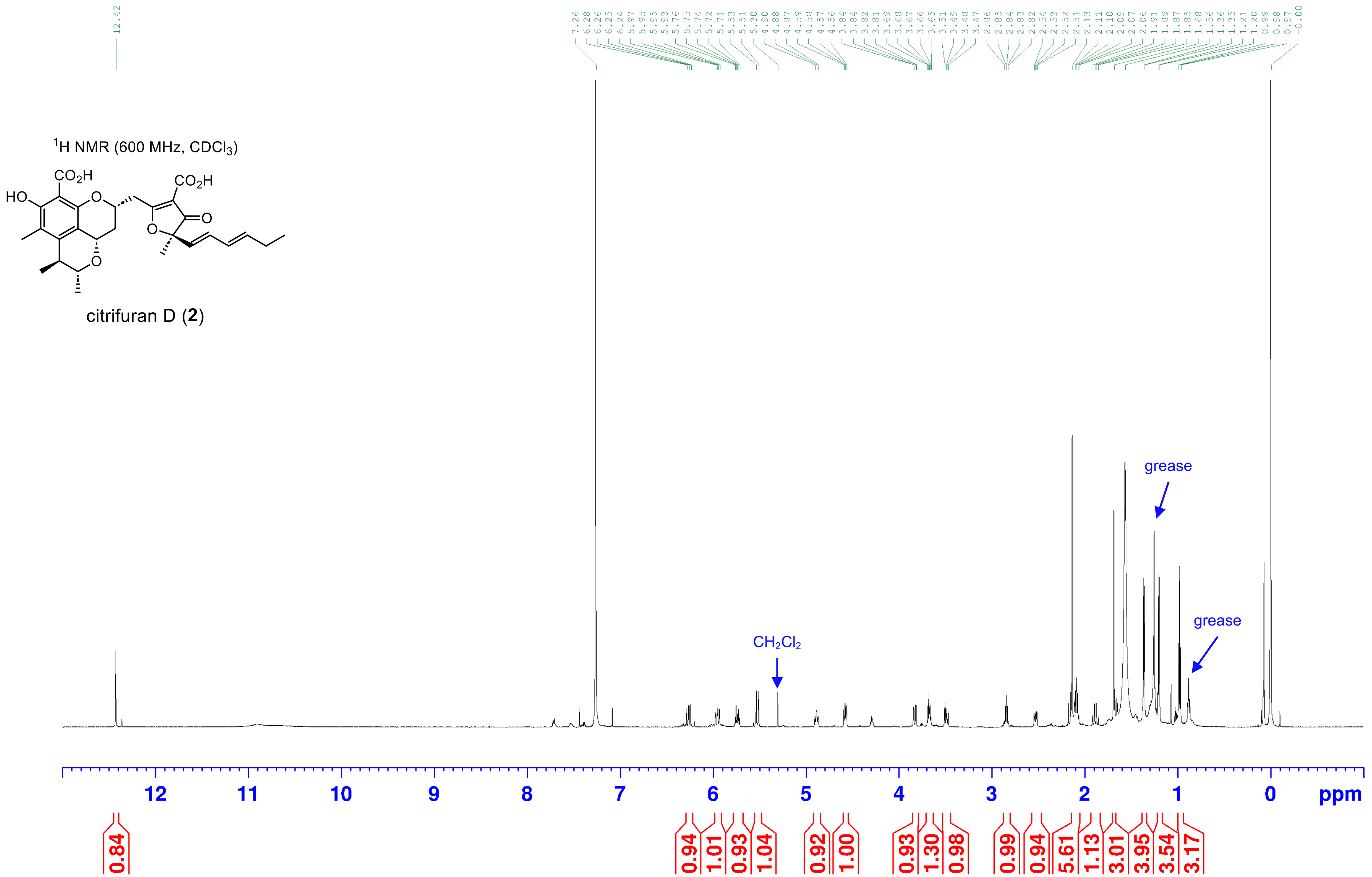

^{1}H NMR (600 MHz, $CDCl_3$)
citrifuran D (2)
grease
grease
CH_2Cl_2
ppm

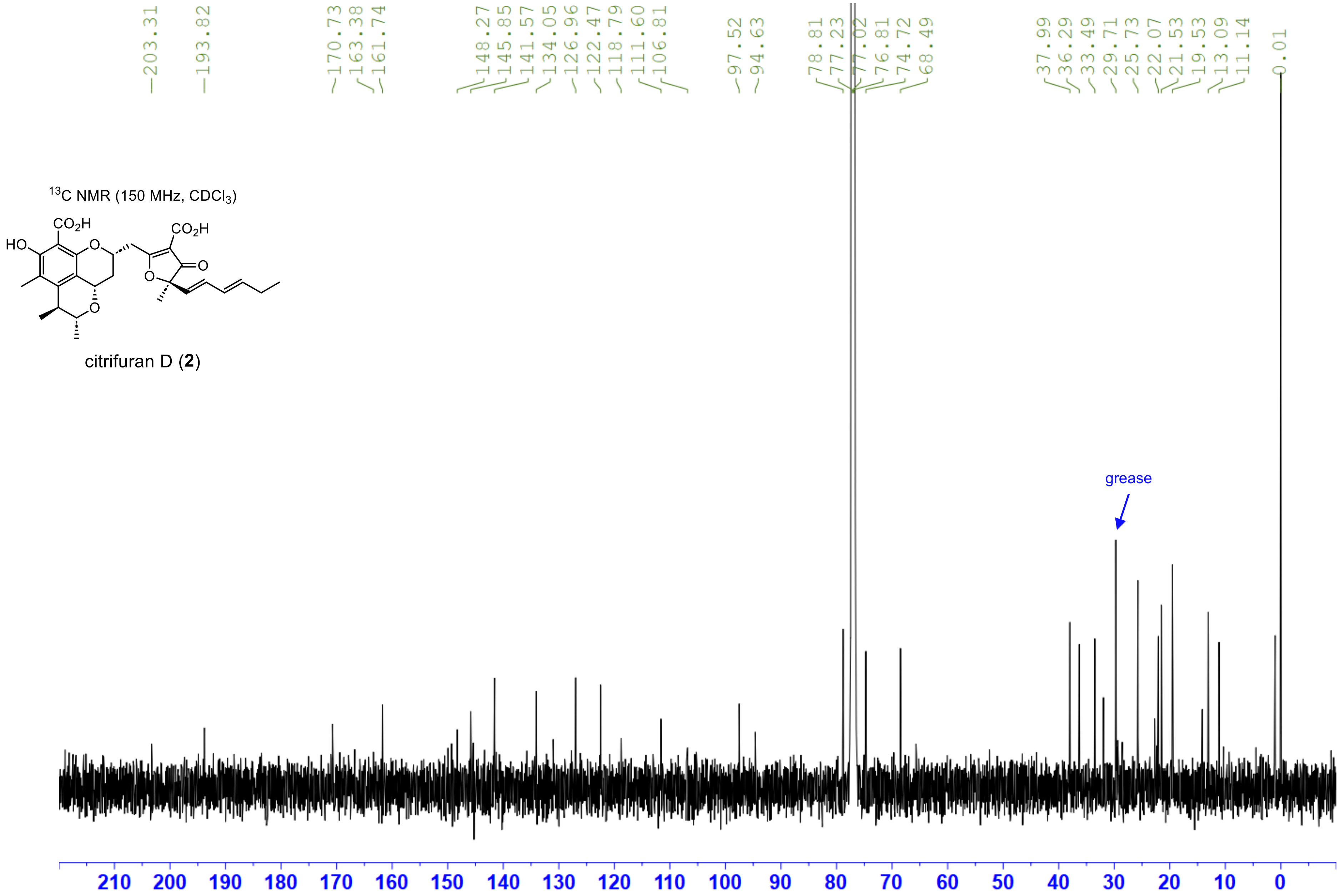
203.31
193.82
170.73
163.38
161.74
148.27
145.85
141.57
134.05
126.96
122.47
118.79
111.60
106.81
97.52
94.63
78.81
77.23
77.02
76.81
74.72
68.49
37.99
36.29
33.49
29.71
25.73
22.07
21.53
19.53
13.09
11.14
0.01
^{13}C NMR (150 MHz, $CDCl_3$)
CO_2H
HO
O
CO_2H
O
O
O
citrifuran D (**2**)
grease
210 200 190 180 170 160 150 140 130 120 110 100 90 80 70 60 50 40 30 20 10 0

^{1}H NMR (600 MHz, CDCl$_3$)

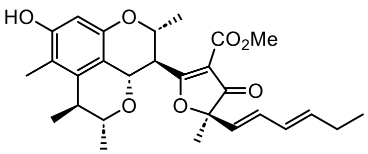

28

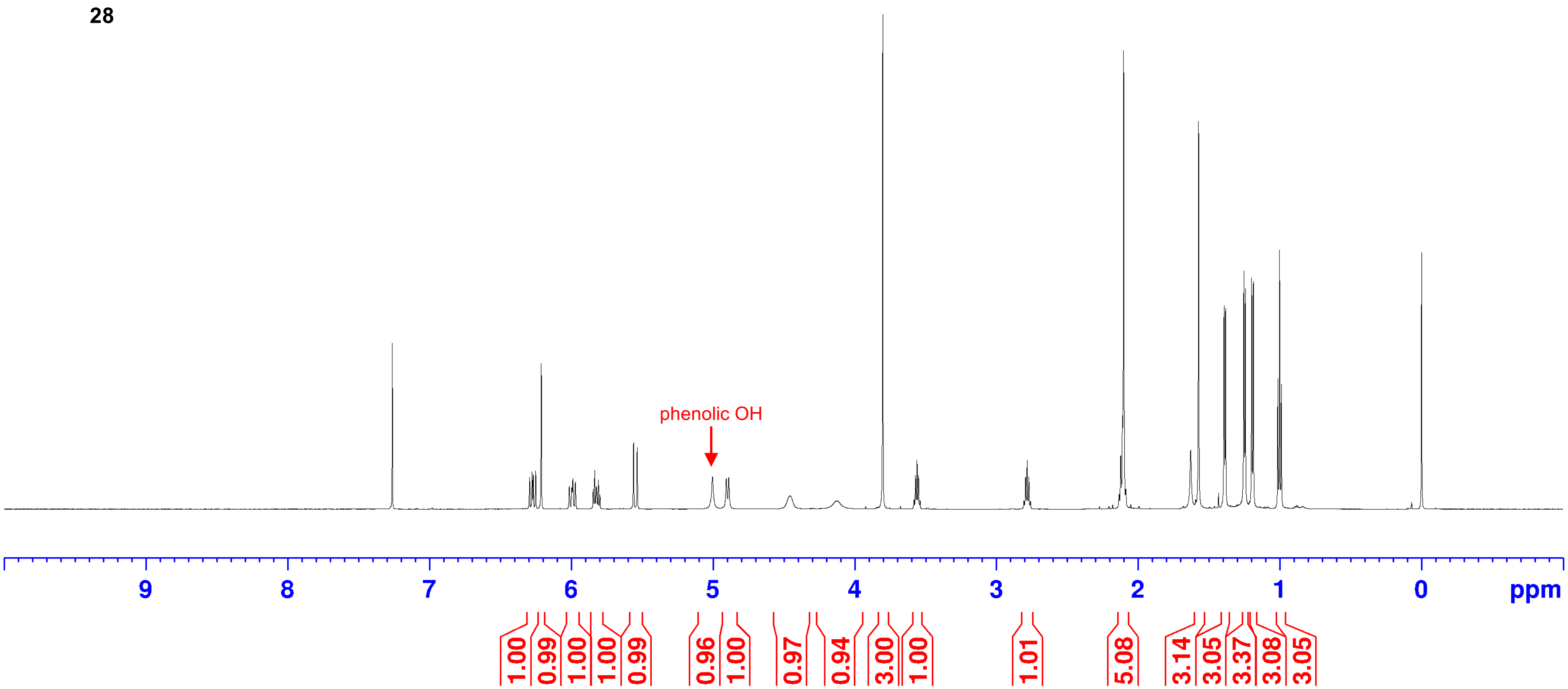

^{13}C NMR (150 MHz, $CDCl_3$)

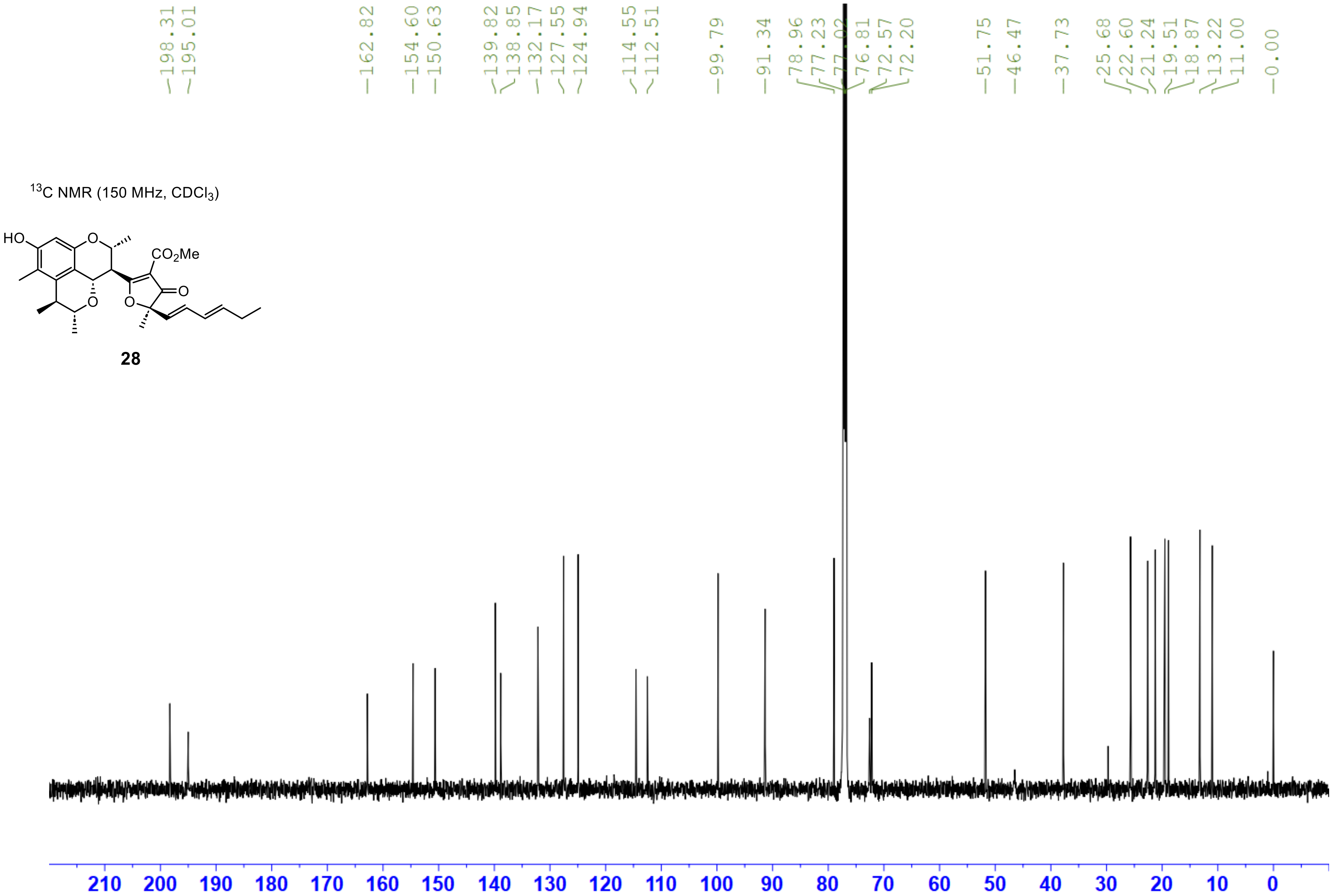

28

HSQC spectrum of 28

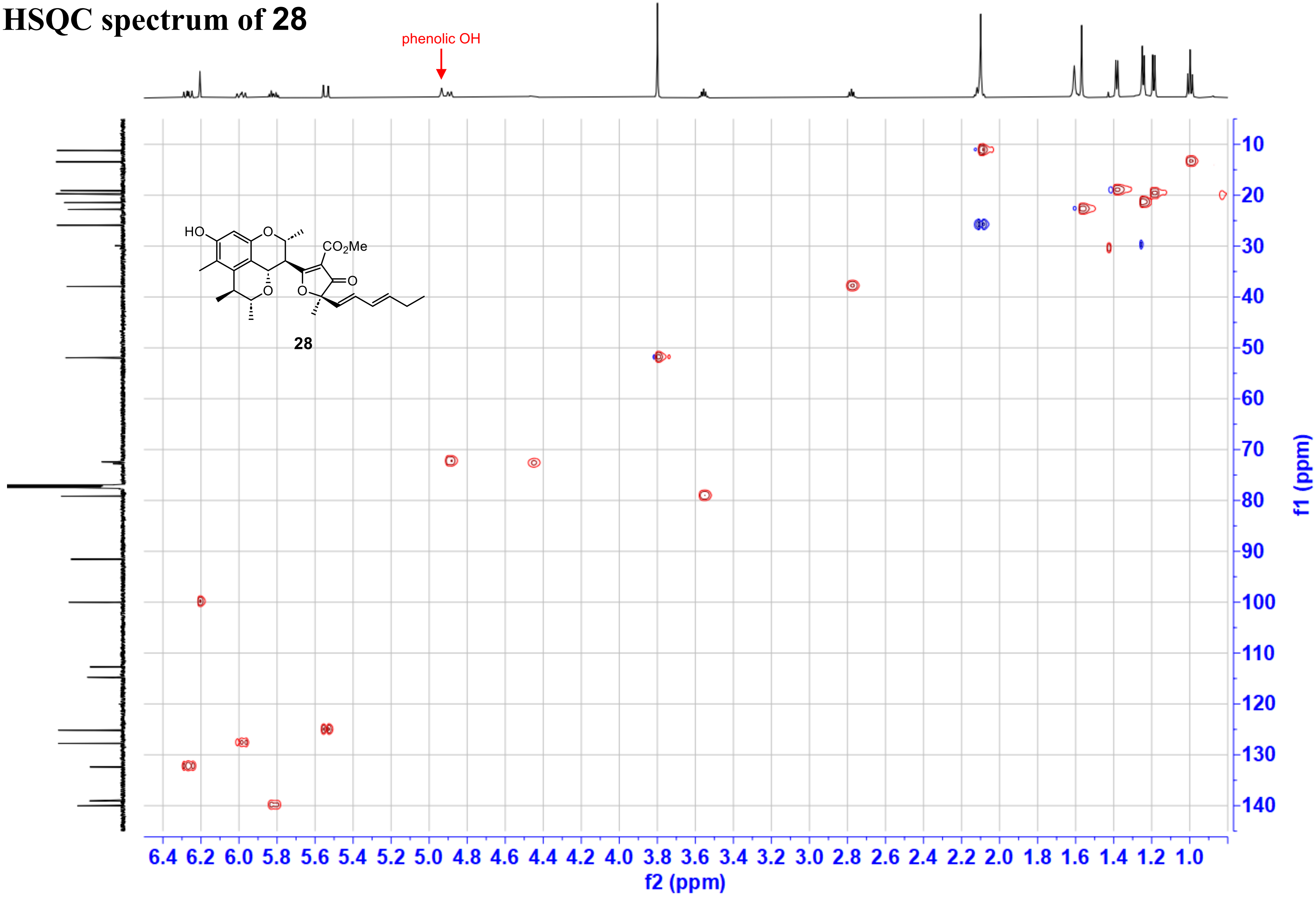

HMBC spectrum of 28

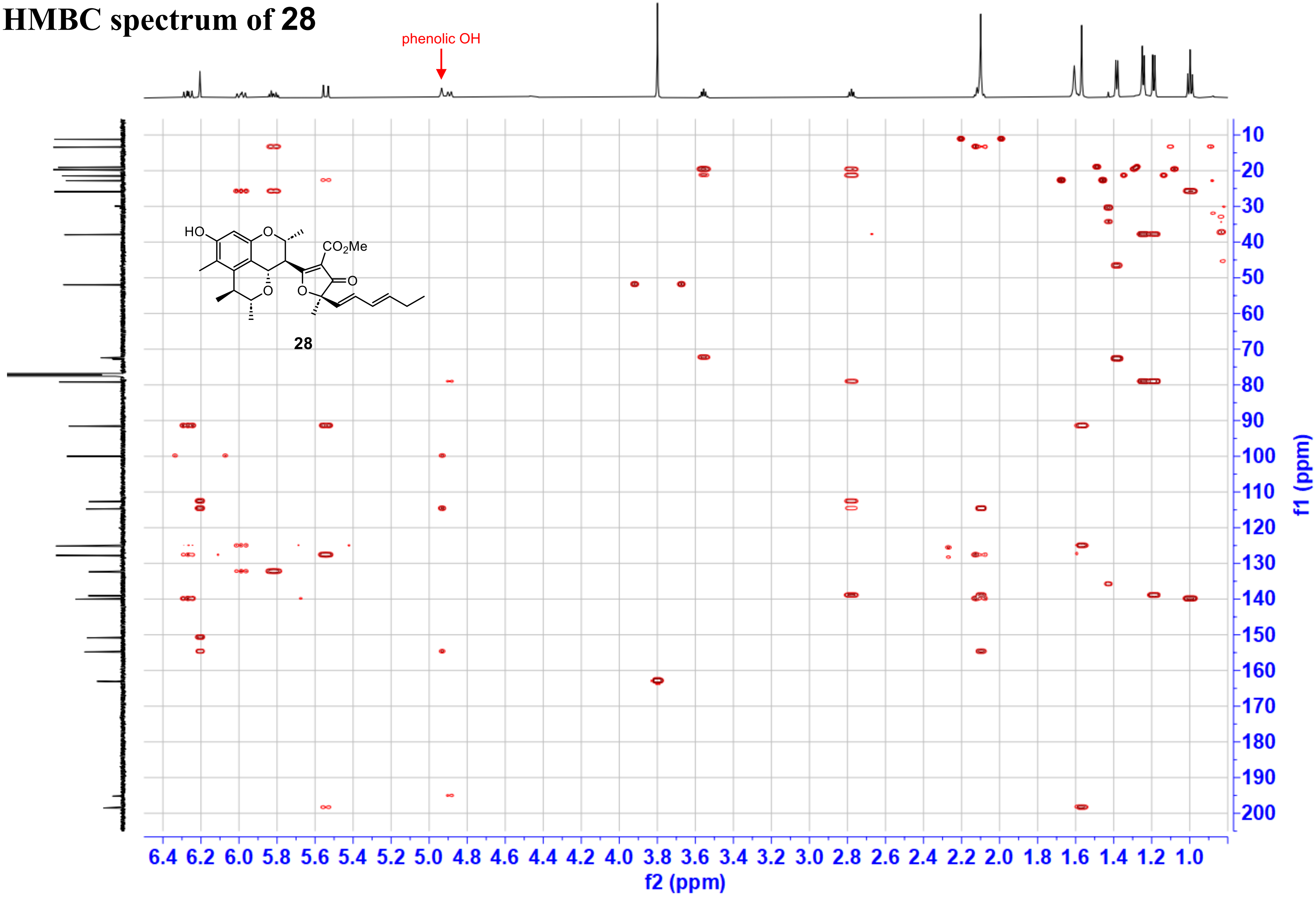

^{1}H NMR (600 MHz, CDCl$_6$)

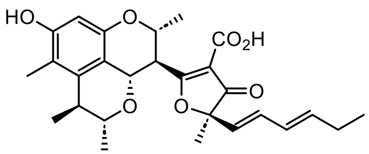

citrifuran A (**1**)

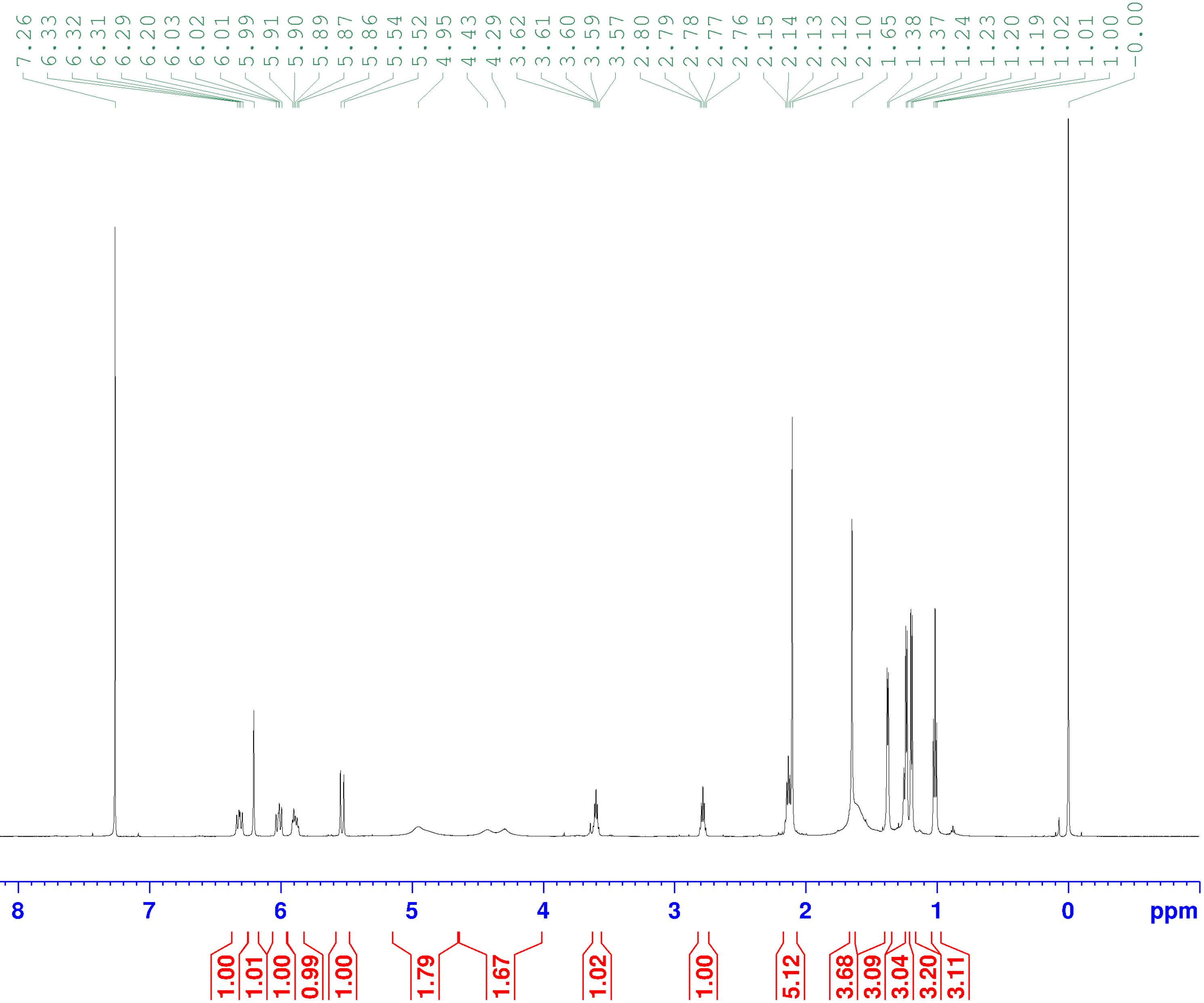

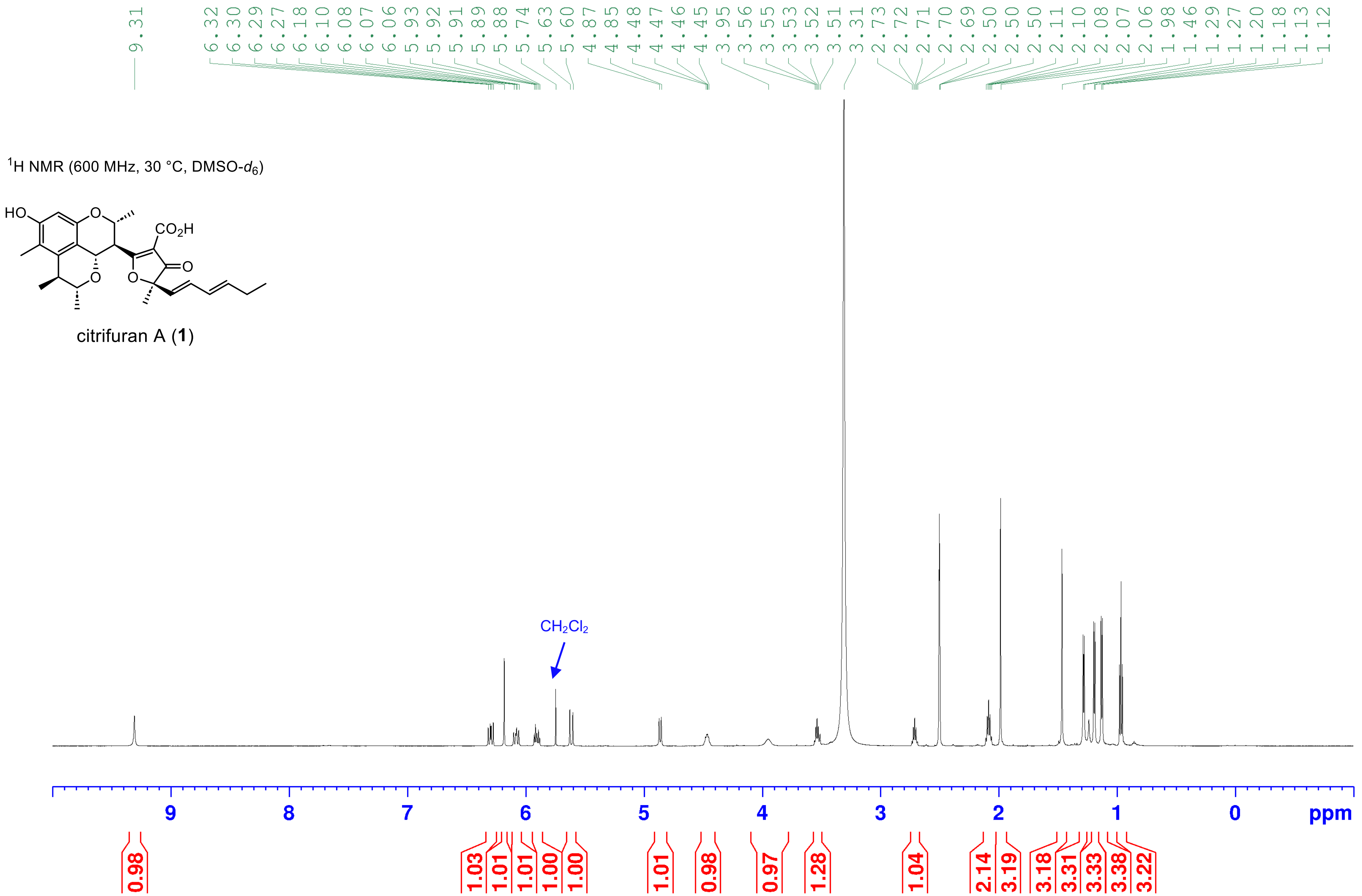

9.31
6.32 6.30 6.29 6.27 6.18 6.10 6.08 6.07 6.06 5.93 5.92 5.91 5.89 5.88 5.74 5.63 5.60 4.87 4.85 4.48 4.47 4.46 4.45 3.95 3.56 3.55 3.53 3.52 3.51 3.31 2.73 2.72 2.71 2.70 2.69 2.50 2.50 2.50 2.11 2.10 2.08 2.07 2.06 1.98 1.46 1.29 1.27 1.20 1.18 1.13 1.12
^{1}H NMR (600 MHz, 30 °C, DMSO-d_6)
HO
O
CO_2H
O
O
O
citrifuran A (**1**)
CH_2Cl_2
9
8
7
6
5
4
3
2
1
0
ppm
0.98
1.03 1.01 1.01 1.00 1.00
1.01
0.98
0.97
1.28
1.04
2.14 3.19
3.18 3.31 3.33 3.38 3.22

^{13}C NMR (150 MHz, 30 °C, DMSO-d_6)

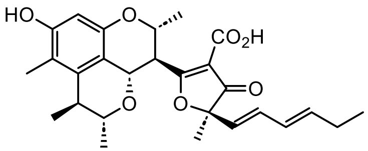

citrifuran A (**1**)

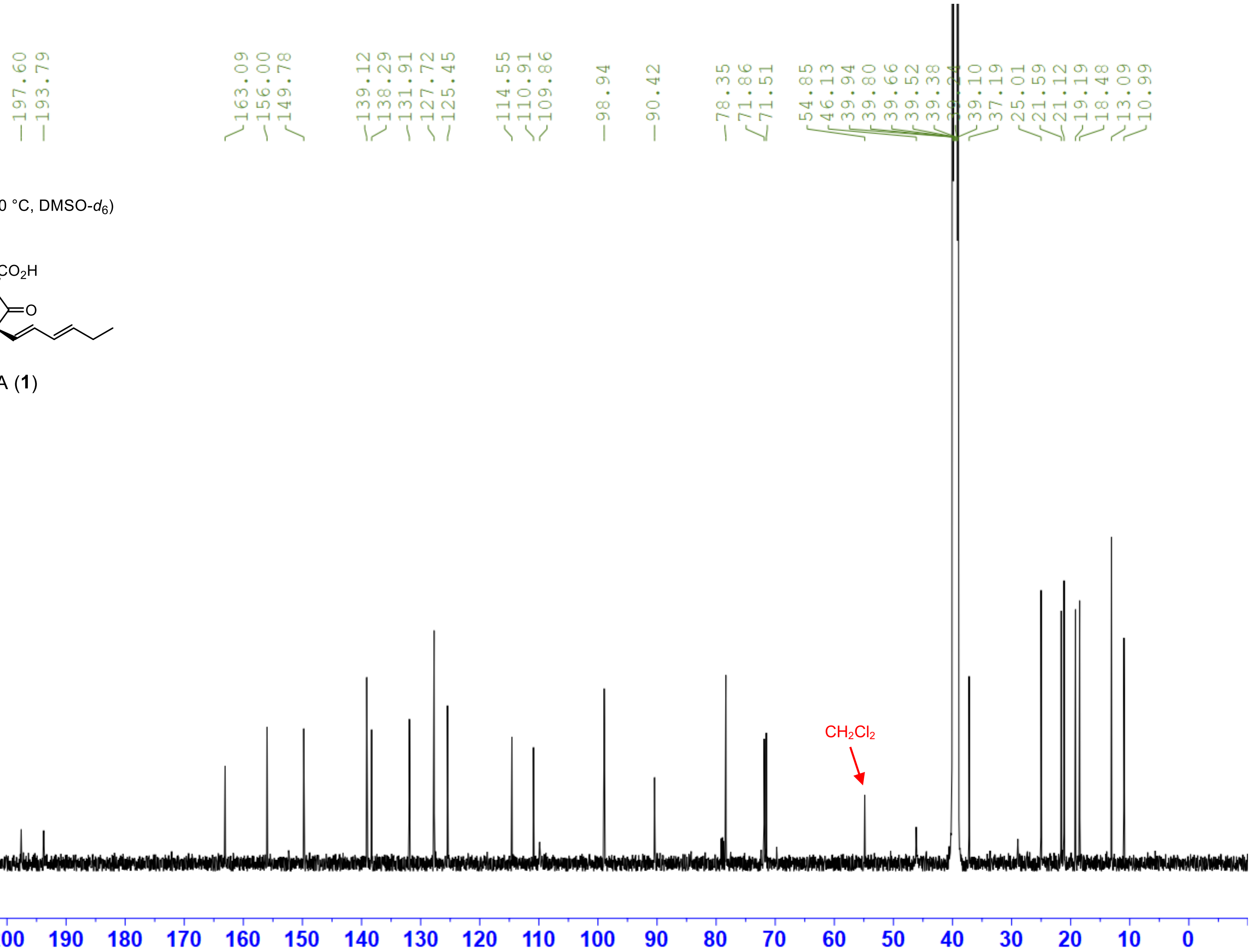

^{1}H NMR (600 MHz, $CDCl_3$)

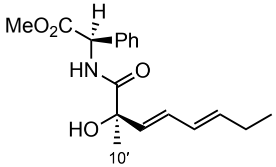

(*R*)-PGME amide of **10**

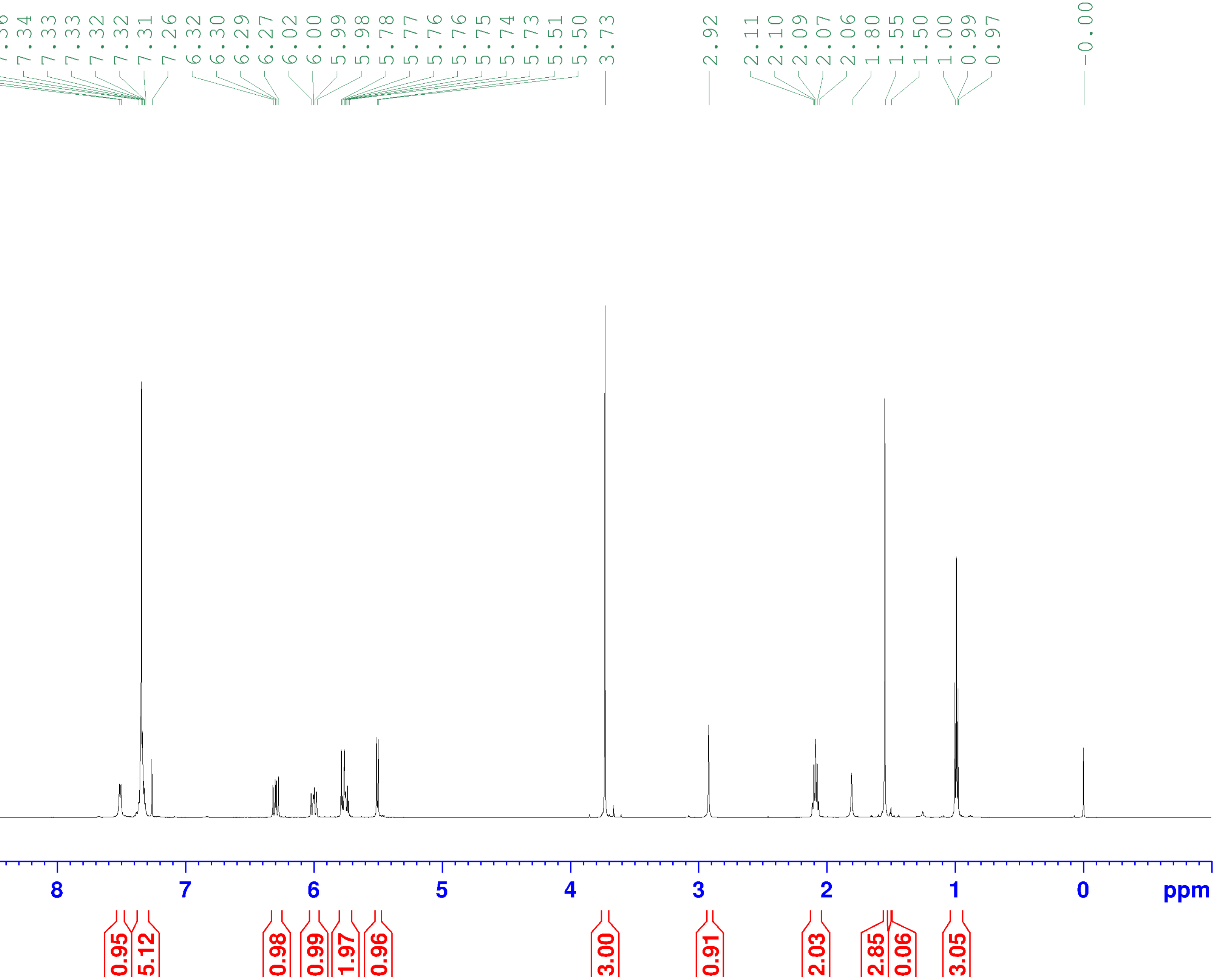

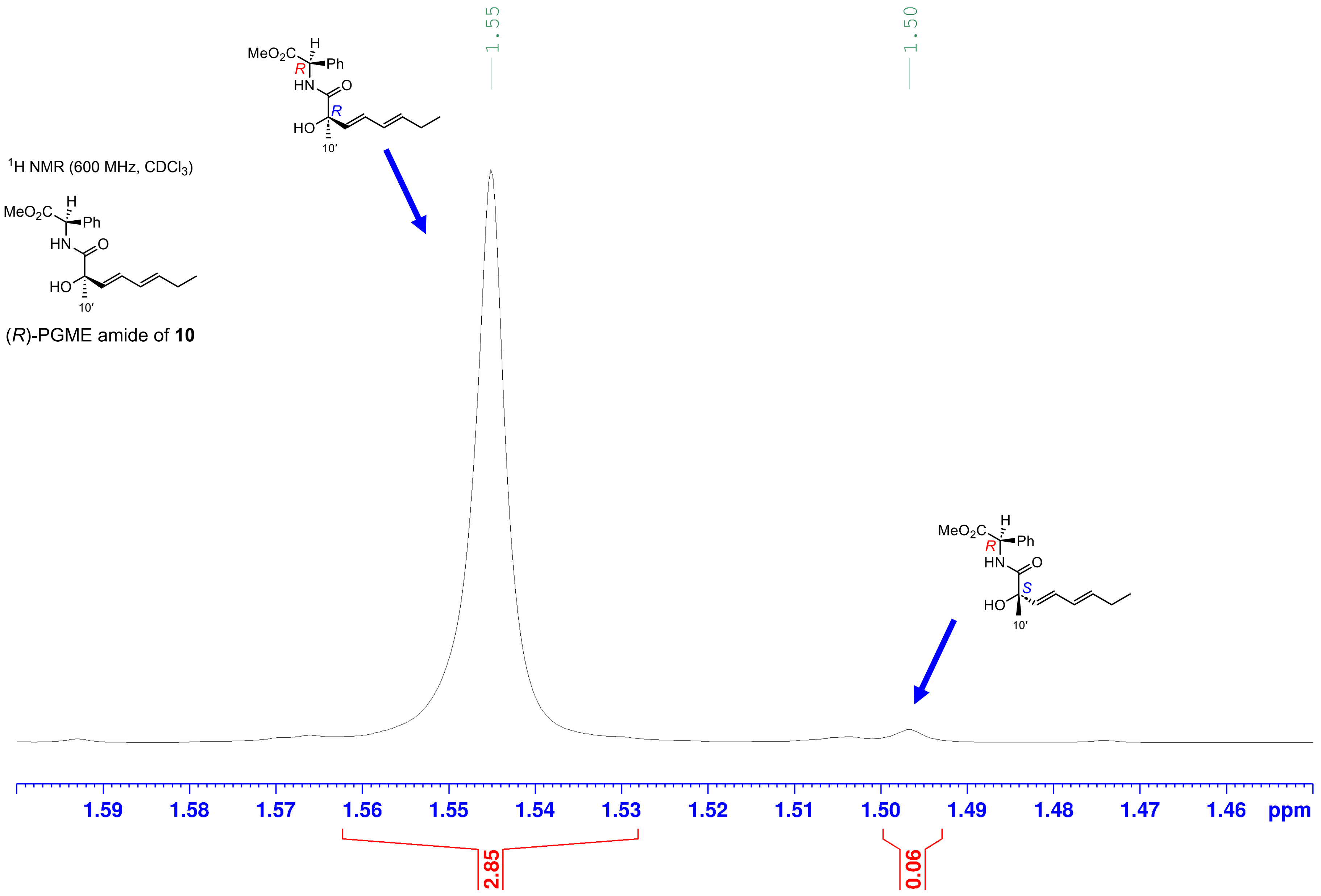

1.55
1.50
MeO2C
H
Ph
R
HN
O
R
HO
10′
1H NMR (600 MHz, CDCl3)
MeO2C
H
Ph
HN
O
HO
10′
(R)-PGME amide of 10
MeO2C
H
Ph
R
HN
O
S
HO
10′
1.59
1.58
1.57
1.56
1.55
1.54
1.53
1.52
1.51
1.50
1.49
1.48
1.47
1.46
ppm
2.85
0.06

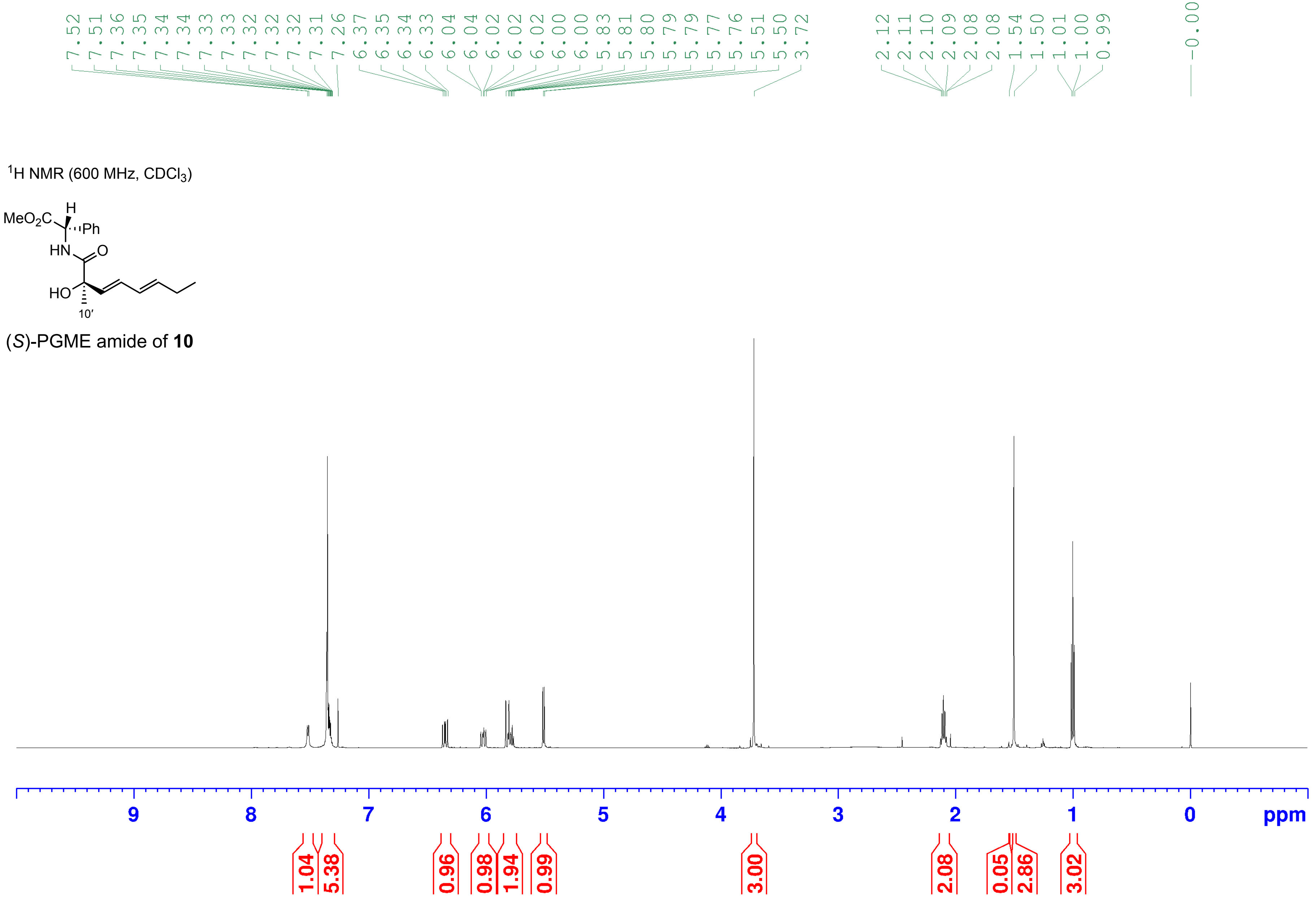

7.52 7.51 7.36 7.35 7.34 7.34 7.33 7.33 7.32 7.32 7.32 7.31 7.26 6.37 6.35 6.34 6.33 6.04 6.04 6.02 6.02 6.02 6.00 6.00 5.83 5.81 5.80 5.79 5.79 5.77 5.76 5.51 5.50 3.72 2.12 2.11 2.10 2.09 2.08 2.08 1.54 1.50 1.01 1.00 0.99 -0.00
1H NMR (600 MHz, CDCl3)
MeO2C
H
Ph
HN
O
HO
10′
(S)-PGME amide of 10
9 8 7 6 5 4 3 2 1 0 ppm
1.04 5.38 0.96 0.98 1.94 0.99 3.00 2.08 0.05 2.86 3.02

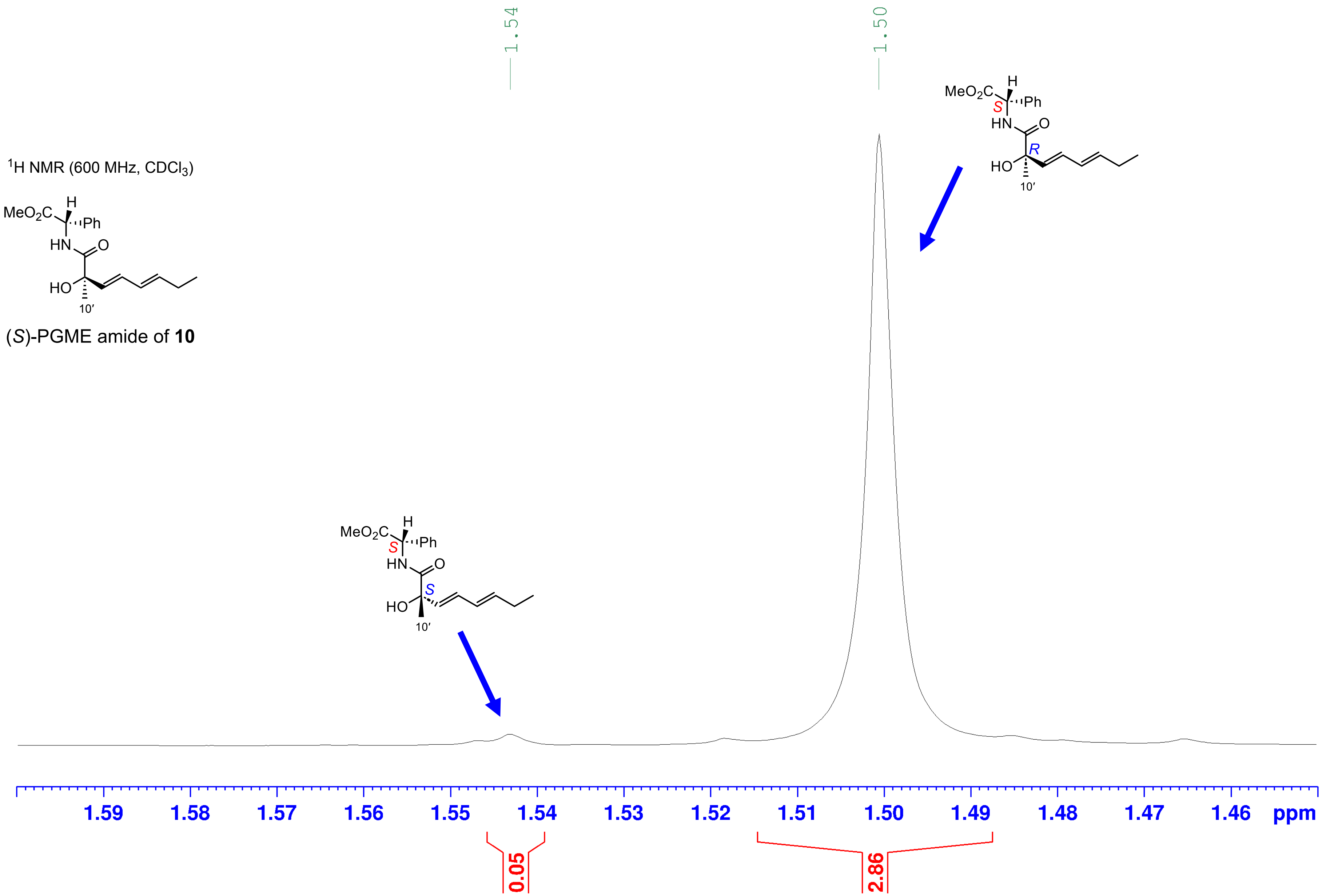
1H NMR (600 MHz, CDCl3)
(S)-PGME amide of 10
MeO2C
H
Ph
HN
O
HO
10′
S
R
1.54
1.50
1.59
1.58
1.57
1.56
1.55
1.54
1.53
1.52
1.51
1.50
1.49
1.48
1.47
1.46
ppm
0.05
2.86
